# APOC1 exacerbates renal fibrosis through the activation of the NF-κB signaling pathway in IgAN

**DOI:** 10.3389/fphar.2023.1181435

**Published:** 2023-05-25

**Authors:** Kuipeng Yu, Lin Ding, Xin An, Yanjiang Yang, Xiaoning Zhang, Luyao Li, Chunjie Wang, Fang Bai, Xiangdong Yang

**Affiliations:** ^1^ Department of Nephrology, Qilu Hospital of Shandong University, Jinan, Shandong, China; ^2^ Department of Blood Purification, Qilu Hospital of Shandong University, Jinan, Shandong, China; ^3^ Laboratory of Basic Medical Sciences, Qilu Hospital of Shandong University, Jinan, Shandong, China; ^4^ Department of Nephrology, Shengli Oilfield Central Hospital, Dongying, Shandong, China

**Keywords:** APOC1, IgAN, biomarker, NK-κB, machine learning algorithms

## Abstract

**Introduction: **IgA nephropathy (IgAN) is the most common disease leading to end-stage renal disease, and tubular fibrosis represents an important risk factor for disease progression. However, research on early molecular diagnostic indicators of tubular fibrosis and the mechanisms underlying disease progression is still lacking.

**Methods:** The GSE93798 dataset was downloaded from the GEO database. DEGs were screened and analyzed for GO and KEGG enrichment in IgAN. The least absolute shrinkage and selection operator (LASSO) and support vector machine recursive feature elimination (SVM-RFE) algorithms were applied to screen for hub secretory genes. The expression and diagnostic efficacy of hub genes were confirmed by the GSE35487 dataset. ELISA was applied to detect the expression of APOC1 in serum. The expression and localization of hub genes in IgAN were verified by the expression of IHC and IF in human kidney tissues, and the correlation of expression with clinical data was verified in the Nephroseq database. Finally, cellular experiments clarified the role of hub genes in the signaling pathway.

**Results:** A total of 339 DEGs were identified in IgAN, of which 237 were upregulated and 102 downregulated. The KEGG signaling pathway is enriched in the ECM–receptor interaction and AGE-RAGE signaling pathway. APOC1, ALB, CCL8, CXCL2, SRPX2, and TGFBI identified six hub secretory genes using the LASSO and SVM-RFE algorithms. *In vivo* and *in vitro* experiments demonstrated that APOC1 expression was elevated in IgAN. The serum concentration of APOC1 was 1.232 ± 0.1812 μg/ml in IgAN patients, whereas it was 0.3956 ± 0.1233 μg/ml in healthy individuals. APOC1 exhibited high diagnostic efficacy for IgAN (AUC of 99.091%, specificity of 95.455%, and sensitivity of 99.141%) in the GSE93798 dataset. APOC1 expression negatively correlated with eGFR (*R* ^2^ = 0.2285, *p* = 0.0385) and positively correlated with serum creatinine (*R* ^2^ = 0.41, *p* = 0.000567) in IgAN. APOC1 exacerbated renal fibrosis, possibly in part by activating the NF-κB pathway in IgAN.

**Conclusion:** APOC1 was identified as the core secretory gene of IgAN, which was closely associated with blood creatinine and eGFR and had significant efficacy in the diagnosis of IgAN. Mechanistic studies revealed that the knockdown of APOC1 could improve IgAN renal fibrosis by inhibiting the NF pathway, which may be a potential therapeutic target for improving renal fibrosis in IgAN.

## 1 Introduction

IgA nephropathy (IgAN) is the most common primary glomerulonephritis and is one of the main causes of end-stage renal disease (Jarrick et al., 2019). The pathogenesis of IgAN is complex; for example, the mucosal immune system (Gesualdo et al., 2021), activation of the complement system (Medjeral-Thomas et al., 2021), and immune inflammation (Kant et al., 2022) are closely related. However, the pathogenesis is still not sufficiently clear. The diagnosis of IgA nephropathy depends mainly on kidney tissue puncture biopsy, and there is no effective non-invasive diagnostic biomarker. In particular, bioinformatics has been extensively used for screening biomarkers and key target molecules in kidney diseases, such as diabetic nephropathy (Zhou et al., 2019; Gao et al., 2021), membranous nephropathy (Cai et al., 2022), and IgAN (Al Mehedi Hasan et al., 2022; Zhang et al., 2022a), which provided the potential for the identification of novel target molecules.

In recent years, machine learning algorithms, artificial intelligence, and other technologies have offered new approaches to a comprehensive understanding of the molecular mechanisms of IgAN (Bulow et al., 2021; Lin et al., 2022). Secretory genes are essential for the diagnosis and treatment of kidney diseases (Chebotareva et al., 2021). SVM-RFE is considered a supervised machine learning algorithm extensively applied to sorting characteristics and selecting genetic features (Li et al., 2022a). LASSO has been widely used for biomarker screening through regression analysis (Zhou et al., 2022). The common genes of LASSO and SVM-RFE are underlying hub genes for the diagnosis of IgAN and play an important role in the progression of the disease. This research synthetically used two machine learning algorithms to screen for secretory genes to identify IgA-specific serological diagnostic markers.

## 2 Materials and methods

### 2.1 Transcriptome data download and analysis

Transcriptome information for kidney tissues from control human (*n* = 22) and IgAN human (*n* = 20) kidneys was downloaded via the NCBI GEO database (GSE93798) (Liu et al., 2017). The transcriptome microarray platform information is GPL22945 (Affymetrix Human Genome U133 Plus 2.0). The methodology for screening DEGs and functional enrichment analysis of DEGs were described in a previous study (Yu et al., 2021).

### 2.2 Secreted protein data processing

Secreted protein data were downloaded from the HPA database (https://www.proteinatlas.org/), and the Venn diagram was performed using the Venny 2.1 online tool (https://bioinfogp.cnb.csic.es/tools/venny/index.html).

### 2.3 SVM-RFE and LASSO algorithms

Support vector machine recursive feature elimination (SVM-RFE) and least absolute shrinkage and selection operator (LASSO) machine algorithms were used to identify candidate hub secretory diagnostic genes. “e1071” and “glmnet” packages were applied to SVM-RFE and LASSO algorithms for gene screening research in R software (Al Mehedi Hasan et al., 2022).

### 2.4 PCA

To validate the efficacy of the genes screened by the two machine algorithms for IgAN differentiation, we performed PCA of the hub genes and 3D spatial mapping using the “scatterplot3d” package.

### 2.5 Correlation and validation analysis in the Nephroseq database

We conducted a correlation analysis of core genes with clinical data (e.g., serum creatinine, estimated glomerular filtration rate, and urea nitrogen) using the Nephroseq database (https://www.nephroseq.org/resource/main.html).

### 2.6 ROC analysis

We evaluated the diagnostic value of core genes for IgAN by performing hub gene ROC analysis with the software package “pROC.” AUC > 0.7 was considered diagnostically significant (Zhang et al., 2022a). Furthermore, GSE35487 was selected as an external dataset whose purpose was to validate the diagnostic efficacy of hub genes.

### 2.7 HE staining of human kidney tissue

Human kidney puncture biopsy tissue was fixed with 4% paraformaldehyde and dehydrated followed by paraffin for kidney tissue fixation. Renal tissue was sectioned to 4 μm thickness using a tissue slicer (Leica, Germany). Hematoxylin–eosin (HE) stain kit (Solarbio, Beijing, China) was used for HE staining of kidney tissues, as described in the instructions. The study was approved by the Ethics Committee of Qilu Hospital, Shandong University (Approval No. KYLL-202111-230).

### 2.8 IHC and IF of human kidney tissue

Human kidney tissue was subjected to Masson, IHC, and IF experiments, as described in a previous study (Yang et al., 2019; Li et al., 2022b). Antibodies and dilution concentrations are described as follows: APOC1 (Abcam, United States, 1:200 dilution), DyLight 488 (Abbkine, United States, 1:1,000, dilution), and DyLight 594 (Abbkine, United States, 1:1,000, dilution). Histochemical sections and Masson-stained kidney tissues were scanned in whole sections.

### 2.9 Cell culture and cell transfection

HK-2 cells were cultivated in RPMI-1640 (Gibco, Waltham, United States) containing 10% fetal bovine serum (FBS, Gibco) and 1% penicillin/streptomycin (Biotop, Beijing, China). HK-2 cells were stimulated using healthy and IgAN patient serum (500 μM). Small inference RNA was designed by Boshang Biotechnology Company to knock down APOC1. Additionally, plasmid-carrying APOC1 was designed by Boshang Biotechnology Company, which was transfected to overexpress APOC1. jetPRIME transfection reagent (Polyplus, France) was applied to conduct the transfection of HK-2 cells. Sequences of small inference RNA and plasmids are in the attached material.

### 2.10 Rt-PCR

The RNAfast200 kit (Fastagen, Shanghai, China) was applied to HK-2 cell mRNA extraction. SureScript™ First-Strand cDNA Synthesis Kit (GeneCopoeia, United States) was used for reversely transcribing into cDNAs. Real-time PCR experiments were performed using the SsoFast™ EvaGreen Supermix Kit (Bio-Rad, CA, United States) and Light-Cycler LC480 real-time PCR system (Roche, Basel, Switzerland). The RT-PCR reaction temperature was as previously described (Li et al., 2022b). The sequence is as follows: homo GAPDH: Forward 5′-GCA​CCG​TCA​AGG​CTG​AGA​AC -3′, Reverse 5′-TGG​TGA​AGA​CGC​CAG​TGG​A -3’; homo APOC1: forward 5′- CAC​ACT​GGA​GGA​CAA​GGC​TC-3′, Reverse 5′- AAA​CCA​CTC​CCG​CAT​CTT​GG-3’.

### 2.11 Western blot

HK-2 cells were cultured in cellular six-well plates. Cellular proteins from different subgroups were extracted for WB electrophoresis as described in the previous research (Liu et al., 2022). The antibodies applied in this research are listed as follows: APOC1(Abcam, United States, 1:1,000 dilution), GAPDH (Proteintech Group, China, 1:4,000 dilution), a-SMA, vimentin, N-cadherin and E-cadherin (Proteintech Group, China, 1:1,000 dilution), NF-κB, and p-NF-κB (CST, USA, 1:1,000 dilution).

### 2.12 ELISA experiment

Sera from IgAN patients and healthy individuals were collected for the ELISA experiment. ELISA test kit was purchased from Abcam Company (United States, No. Ab108808). Experimental procedures are performed in the installation instructions.

### 2.13 Statistical analysis

Statistical analysis and graphics are based on R software (R 4.2.1). Student’s *t*-test was used to compare the two groups. *p* < 0.05 was defined as statistically significant, *p < 0.05, **p < 0.01, and ***p < 0.001.

## 3 Results

### 3.1 DEG screening and functional enrichment analysis

**FIGURE 1:**
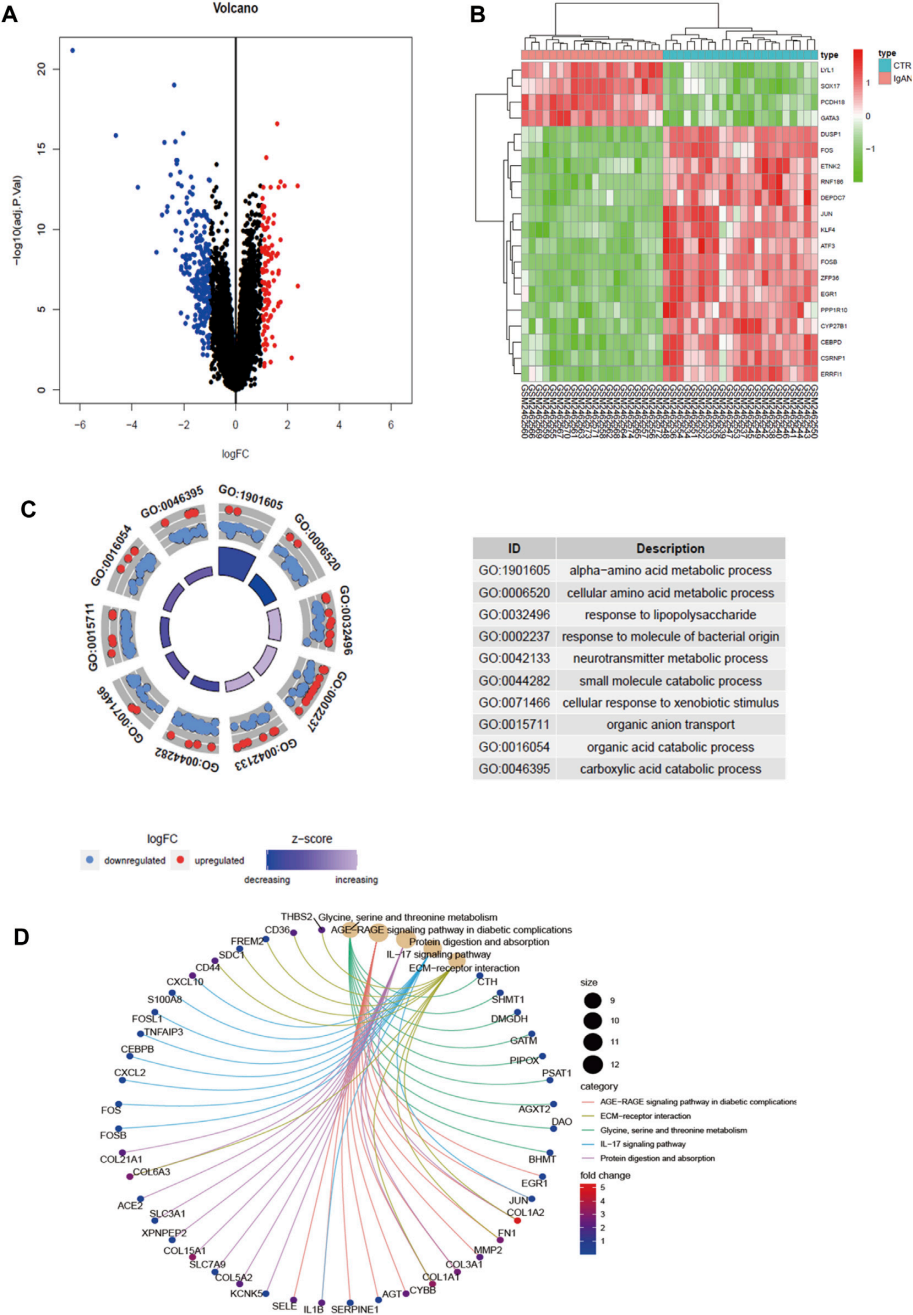
Differential gene screening and functional analysis in GSE93798. **(A)** Volcano exhibits between Ctrl and IgAN. **(B)** Heatmap demonstrating the top 20 DEGs. **(C)** GO enrichment analysis of DEGs. **(D)** KEGG enrichment analysis of DEGs. DEGs, differentially expressed genes; Ctrl (*n* = 22), control human kidney transcriptomic; IgAN (*n* = 20), IgAN human kidney transcriptomic.

A total of 22 healthy controls and 20 IgAN patients were enrolled in this research. We identified 339 DEGs, which contained 237 upregulated genes and 102 downregulated genes in IgAN (Table 1; Supplementary Tables S1, S2). The results are demonstrated by the volcano and heatmaps (Figures 1A, B). Furthermore, GO functional analysis of DEGs was conducted, and our research revealed significant correlations with the alpha-amino acid metabolic process, cellular amino acid metabolic process, and response to lipopolysaccharide (Figure 1C). The KEGG signaling pathway is enriched in the ECM–receptor interaction, AGE-RAGE signaling pathway in diabetic complications, and IL-17 signaling pathway (Figure 1D). The outcomes are summarized in Supplementary Tables S3, S4.

**TABLE 1:** Top 10 upregulated and downregulated genes identified in the gene expression microarray dataset (22 healthy controls vs. 20 IgAN patients).

Gene	LogFC	*p*-value	Adjusted *p*-value
*FOSB*	6.289,355	4.05E-26	7.31E-22
*FOS*	4.622,179	3.75E-20	1.35E-16
*APOLD1*	3.769,007	3.05E-16	2.29E-13
*CXCL2*	3.052188	2.93E-11	2.54E-09
*CD69*	2.840,102	5.38E-14	1.31E-11
*EGR1*	2.745,972	1.49E-19	3.83E-16
*BRE-AS1*	2.64709	2.41E-14	7.61E-12
*RASD1*	2.630,472	1.13E-14	4.01E-12
*DEPDC7*	2.506,611	3.03E-17	3.90E-14
*NR4A2*	2.442,257	1.99E-15	9.70E-13
*LUM*	−2.40223	1.26E-08	3.36E-07
*COL1A2*	−2.39422	2.60E-16	2.04E-13
*HLA-DQA1*	−2.16467	0.00298	0.01048
*GATA3*	−1.88637	2.58E-16	2.04E-13
*SOX17*	−1.73882	1.11E-16	1.11E-13
*HTR2B*	−1.73663	2.00E-07	3.50E-06
*ECM1*	−1.73384	3.45E-12	4.41E-10
*COL1A1*	−1.66998	8.59E-10	3.87E-08
*TAC1*	−1.65975	2.29E-07	3.96E-06
*C8orf4*	−1.65763	3.30E-16	2.29E-13

### 3.2 SVM-RFE and LASSO algorithm screening for hub secretory genes

**FIGURE 2:**
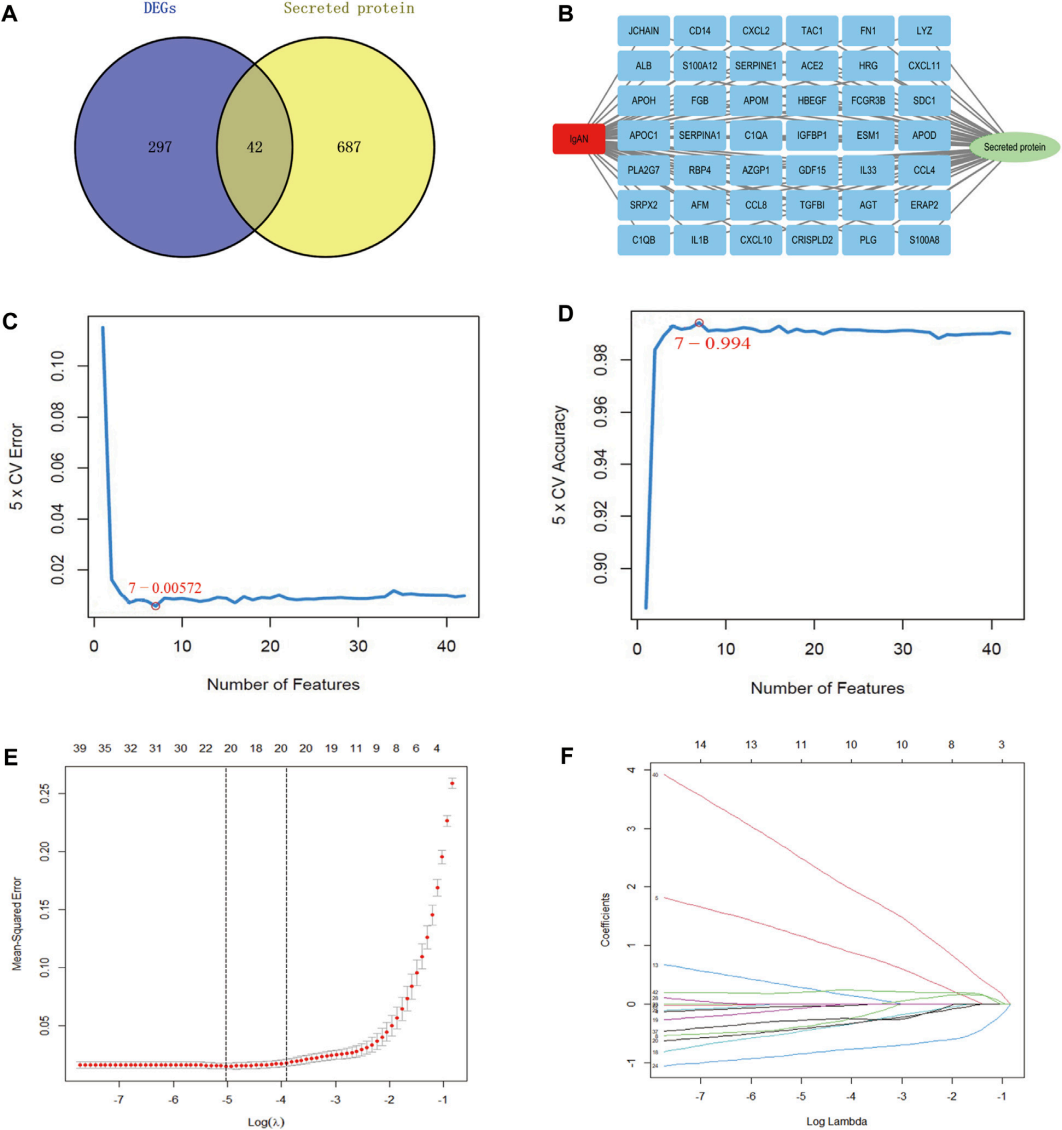
Screening of hub secretory genes using the SVM-RFE and LASSO algorithms. **(A)** Venn diagram demonstrating secreted genes in DEGs. **(B)** Exhibition of 42 hub secretory genes in IgAN. **(C, D)** LASSO analysis screens for IgAN diagnostic biomarkers in core secretory genes. **(E, F)** SVM-RFE machine learning screening for secretory IgAN diagnostic biomarkers.

The proteins secreted by human tissues are essential to understanding human biology and identifying potential targets for future diagnostics and therapy. Therefore, we detected 42 secreted genes among 339 DEGs (Figure 2A), and the 42 genes are illustrated in Figure 2B. SVM-RFE and LASSO algorithms have been applied to the screening of genes. In the SVM-RFE model, seven feature genes were identified: CXCL2, SRPX2, CCL8, APOC1, ALB, TGFBI, and HBEGF (Figures 2C, D). Within LASSO models, tenfold cross-validation was performed with 11 characteristic genes: ALB, APOC1, APOM, CCL8, CXCL2, ERAP2, ESM1, GDF15, SDC1, SRPX2, and TGFBI (Figures 2E, F). The results are summarized in Supplementary Tables S5–S7.

### 3.3 Validation of hub secretory genes

**FIGURE 3:**
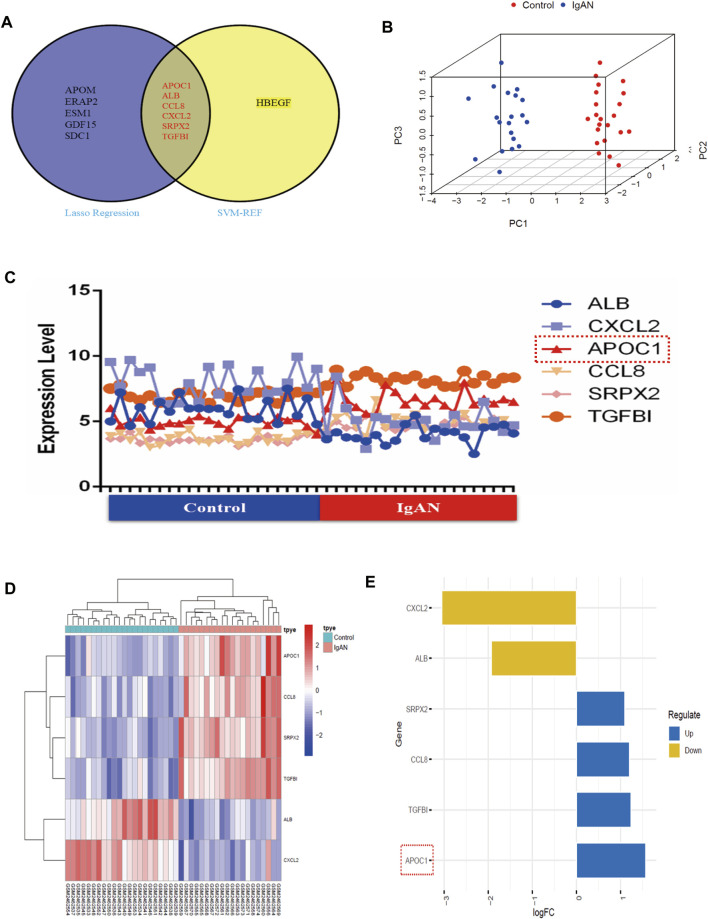
SVM-RFE and LASSO machine learning in screening IgAN diagnostic biomarkers in GSE93798. **(A)** Venn map of common genes between SVM-RFE and LASSO machine learning. **(B)** PCA’s 3D graphics demonstrates six secretory genes to differentiate between healthy and IgAN patients. **(C–E)** Collapsed line, heat, and bar charts indicating the expression of the six hub secretory genes in the dataset.

Regarding the results of the two algorithms, after taking the intersection, we identified six hub secretory genes. APOC1, ALB, CCL8, CXCL2, SRPX2, and TGFBI are illustrated in the Venn diagram (Figure 3A). To verify the accuracy of the outcomes of the two algorithms, we demonstrated spatial differentiation between the IgAN and control groups using the PCA algorithm for the six genes that were screened. Six hub secretory genes were successfully distinguished between the IgAN and control groups (Figure 3B). Additionally, the expression of the six core secretory genes is illustrated by line, heatmap, and bar charts (Figures 3C–E).

### 3.4 APOC1 expression and diagnostic efficacy

**FIGURE 4:**
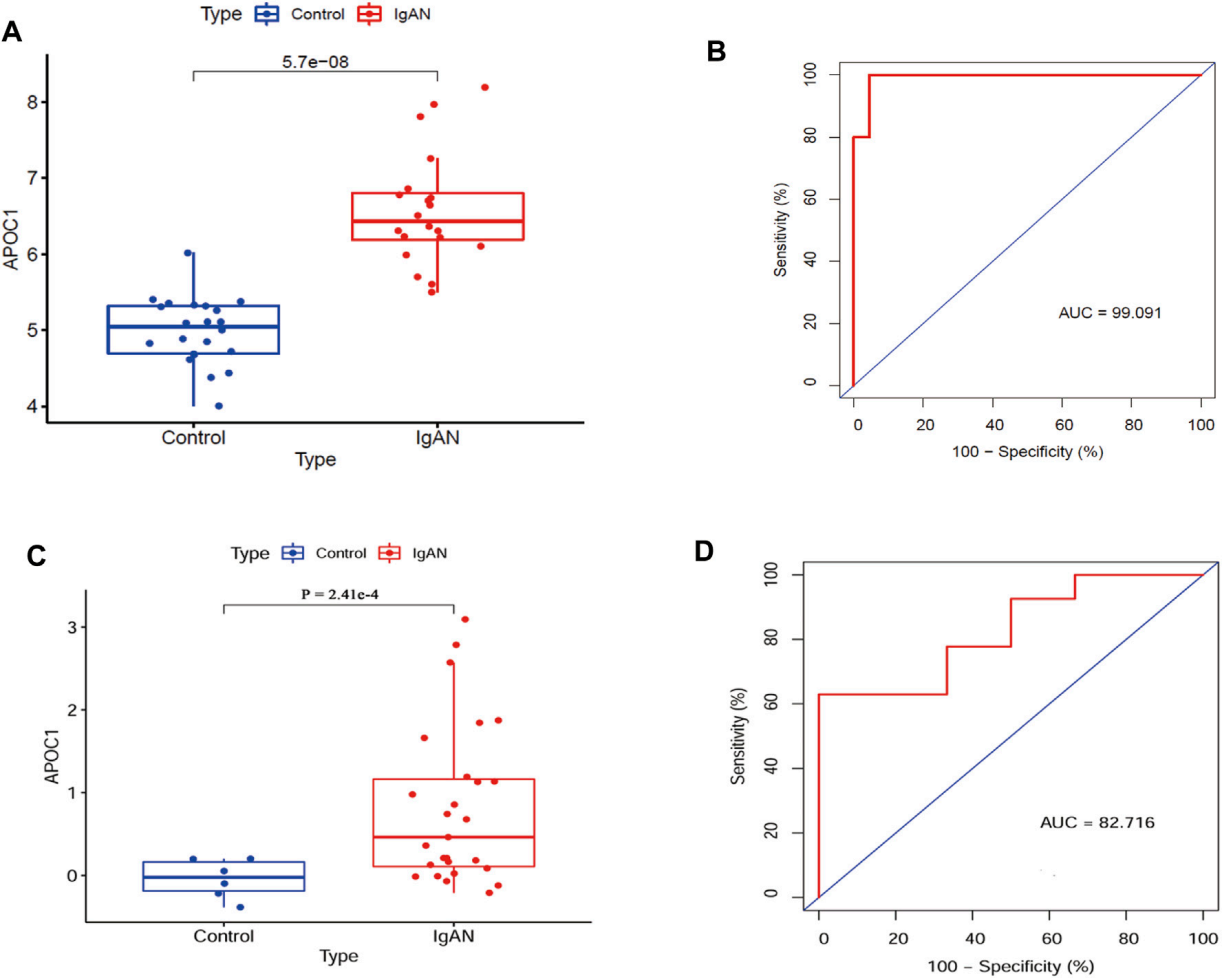
Expression of APOC1 is elevated in IgAN with diagnostic function. **(A, B)** APOC1 expression and ROC diagnostic efficacy in the GSE93798 dataset (Ctrl = 22, IgAN = 20, AUC = 99%, sensitivity = 99%, and specificity = 95%, *p* < 0.001). **(C, D)** APOC1 expression and ROC diagnostic efficacy in the GSE35487 dataset (Ctrl = 6, IgAN = 25, AUC = 82.7%, sensitivity = 63%, and specificity = 99%, *p* < 0.001). Ctrl:healthy, IgAN, IgAN patients. *p < 0.05, **p < 0.01, ***p < 0.001.

Figures 4A, B suggest that APOC1 expression is elevated in patients with IgAN (*p* < 0.001). To assess the diagnostic accuracy of APOC1 in predicting IgAN, receiver operating characteristics were evaluated. The research demonstrates that the AUC was 99.091% (95% CI: 97.131–99.921), specificity was 95.455%, and sensitivity was 99.141%. In addition, the results of the experiment were validated by the GSE35487 dataset. The outcome of the research is consistent with the aforementioned results, indicating that APOC1 expression was elevated in IgAN (Figure 4C), and the AUC was 82.716% (95% CI: 66.115-99.317), specificity was 99.515%, and sensitivity was 62.963% (Figure 4D).

### 3.5 Correlation between APOC1 and IgA patients’ clinical data

**FIGURE 5:**
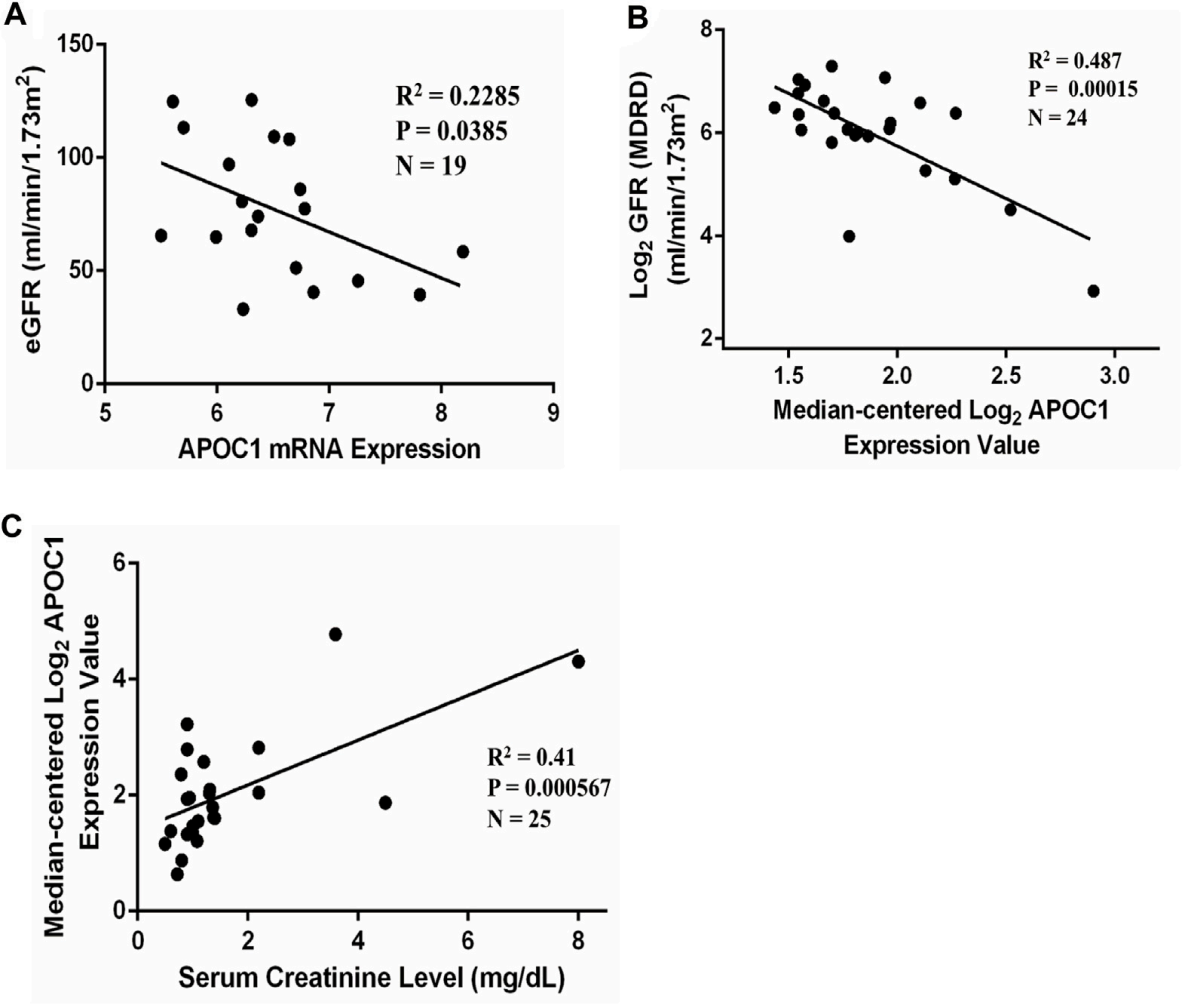
APOC1 expression is correlated with eGFR and serum creatinine in patients with IgAN. **(A)** APOC1 expression was negatively correlated with eGFR in GSE93798 (*n* = 19, *R* ^2^ = 0.2285, *p* = 0.0385). **(B)** Negative correlation between APOC1 expression and GFR in the Nephroseq database (*n* = 24, *R* ^2^ = 0.487, *p* < 0.001). **(C)** Positive correlation between APOC1 expression and creatinine (*n* = 25, *R* ^2^ = 0.41, *p* < 0.001).

Figure 5A illustrates that APOC1 expression negatively correlates with eGFR in IgAN (*n* = 19, *R* ^2^ = 0.2285, *p* = 0.0385). Furthermore, we have analyzed the correlation between APOC1 and clinical data through the Nephroseq database for IgAN. The conclusions of the research also revealed that the expression of APOC1 negatively correlated with eGFR (*n* = 24, *R* ^2^ = 0.487, *p* = 0.00015) (Figure 5B). Nevertheless, the expression of APOC1 positively correlated with serum creatinine (*n* = 25, *R* ^2^ = 0.41, *p* = 0.000567) (Figure 5C).

### 3.6 Validation of APOC1 expression in IgA patients

**FIGURE 6:**
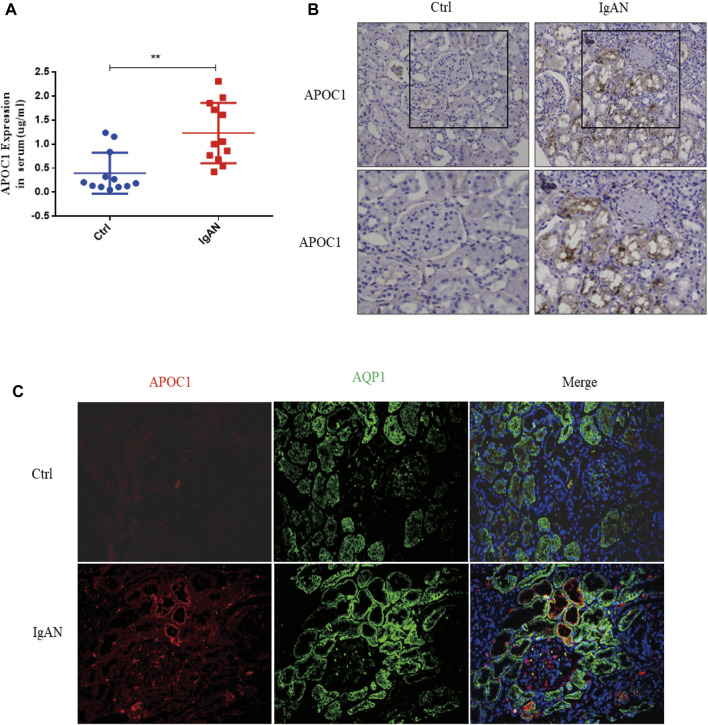
Elevated expression of APOC1 in IgAN patients. **(A)** Expression of APOC1 in serum. **(B)** Representative IHC illustrating elevated expression of APOC1 in IgAN. **(C)** Representative IF identifies elevated APOC1 expression in renal tubular epithelial cells of IgAN patients. IHC, immunohistochemical; IF, immunofluorescence; AQP1, aquaporin 1 (proximal renal tubular markers), **p < 0.01 vs. Ctrl.

A total of 12 patients with IgAN nephropathy were enrolled in this research. The serum concentration of APOC1 in IgAN is 1.232 ± 0.1812 μg/ml, whereas it is 0.3956 ± 0.1233 μg/ml in healthy individuals (Figure 6A). The IHC research revealed elevated expression of APOC1 in IgAN kidney tissue using normal kidney tissue from cancer as a control group (Figure 6B). The results were consistent with the IHC results, and APOC1 was predominantly detected in renal tubular epithelial cells in IF (Figure 6C).

### 3.7 APOC1 exacerbates human renal tubular epithelial cells through the activation of the NF-κB signaling pathway

**FIGURE 7:**
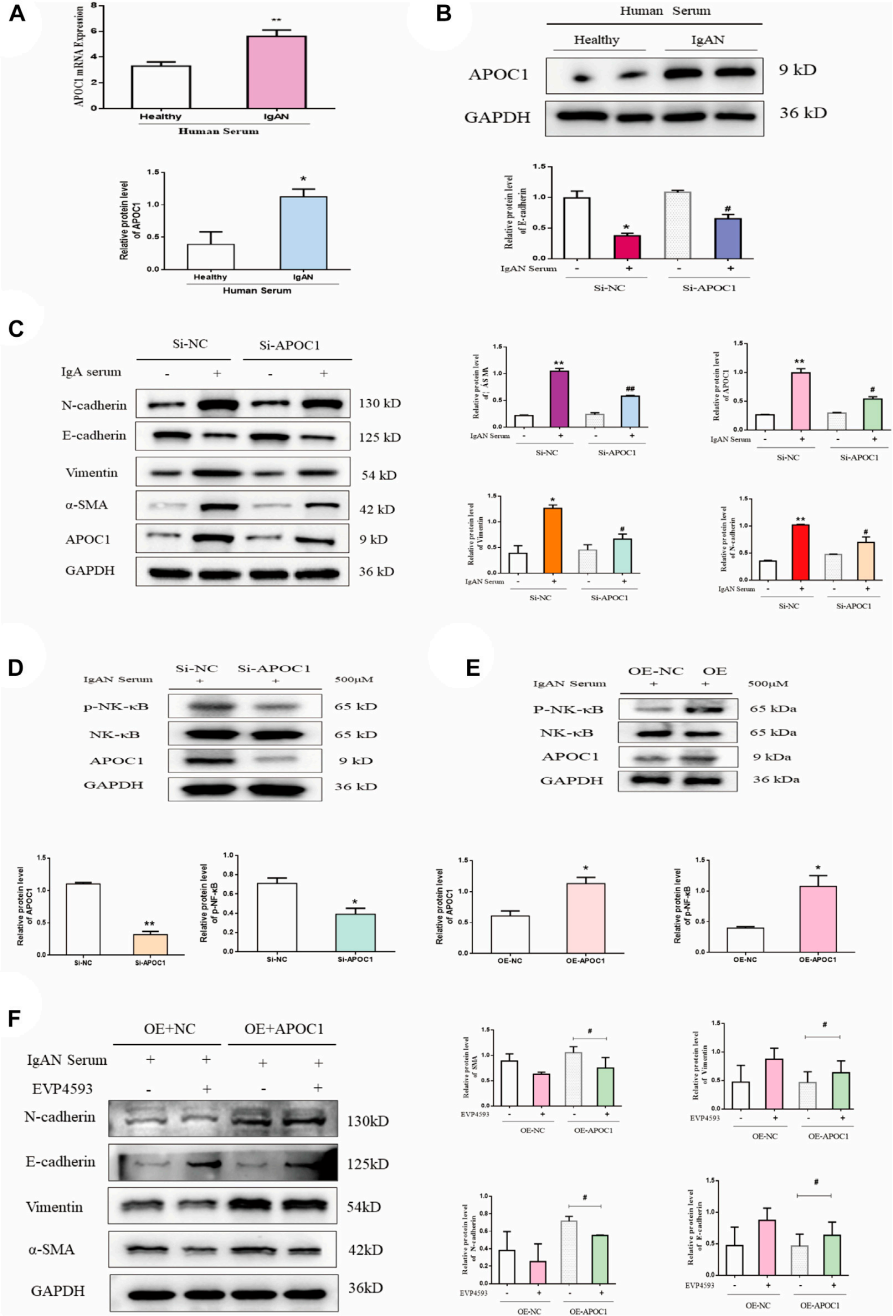
APOC1 exacerbates human renal tubular epithelial cell fibrosis through activation of the NF-κB signaling pathway. **(A)** Expression of APOC1 mRNA in HK-2 cells after serum stimulation. Healthy: healthy serum, IgAN: IgAN serum. **(B)** Representative WB demonstrates APOC1 expression. **(C)**. Representative WB demonstrates APOC1 and fibrosis-related protein expression (E-cadherin, N-cadherin, vimentin, and α-SMA). **(D)** Si-APOC1 expression inhibition of the NF-κB pathway. **(E)** OE-APOC1 expression activation of the NF-κB signaling pathway. **(F)** Representative WB demonstrates fibrosis-related protein expression. Stimulation of HK-2 cells with the serum concentration of 500 μmol. Data are presented as mean ± SEM, *p < 0.05 vs. healthy (Si-NC, OE-NC), ^#^ *p* < 0.05 vs. Si-NC, ^##^ *p* < 0.01 vs. Si-NC.

Using serum from IgAN patients to stimulate HK-2 cells, we discovered that APOC1 expression was elevated by PCR and WB (*p* < 0.05, Figures 7A, B). Fibrosis-associated protein results revealed that interference with APOC1 expression could inhibit HK-2 cells’ epithelial–mesenchymal transition (Figure 7C). Interfering with APOC1 expression decreases P65 phosphorylation, whereas overexpressing APOC1 increases P65 phosphorylation (Figures 7D, E). More importantly, the results showed that APOC1 may partly activate NF-κB signaling in IgAN. We conducted experiments using EVP4593—an inhibitor of the NF-κB pathway. Our research results suggest that the inhibition of the NF-κB pathway partially prevented APOC1 causing fibrosis in kidney tissue (Figure 7F).

## 4 Discussion

IgAN is one of the most common primary glomerular diseases in the world (Sim et al., 2016; Schena and Nistor, 2018). Research reveals that in 20%–30% of patients with IgAN, renal fibrosis progresses progressively to end-stage renal disease within 20 years (Jarrick et al., 2019; Storrar et al., 2022). IgAN is characterized by the deposition of IgA-based immunoglobulin molecules in the mesangial region of the kidney (Matsumoto et al., 2022), interstitial fibrosis, and tubular atrophy (Juan et al., 2022). However, the exact pathogenesis of IgAN remains unclear, and specific diagnostic markers and effective therapeutic targets are currently unavailable. Therefore, renal histopathology and immunopathological examination are indispensable to confirm the diagnosis of IgAN.

For this research, the GSE93798 dataset was downloaded from the GEO database to screen DEGs from IgAN patients. We determined 339 DEGs and conducted heat and volcano mapping of the DEGs. In addition, the KEGG enrichment of DEGs was analyzed in ECM–receptor interaction and IL-17 signaling pathway, which was consistent closely with previous studies (Figure 1). A total of 42 secretory genes were filtered from the DEGs and six key candidates were identified (APOC1, ALB, CCL8, CXCL2, SRPX2, and TGFBI) by the SVM and LASSO algorithms (Figures 2, 3). In the end, APOC1 was identified as a new hub gene for IgAN (Figure 4). What is extremely interesting is that we discovered that APOC1 may also be a hub secretory gene in IgAN. Therefore, we performed immunohistochemistry and immunofluorescence of APOC1 in patients with IgAN. APOC1 expression is elevated in the tubules of patients with IgAN, whereas glomerular expression of APOC1 is elevated in patients with DN (Yu et al., 2023). Expression of APOC1 in different kidney cells implicates that there might be different functions.

Apolipoprotein C1 (APOC1) is a member of the apolipoprotein family involved in lipid metabolism (Berbee et al., 2005; Bouillet et al., 2016) and immune inflammation. Research revealed that APOC1 plays a role in the pathogenesis of glomerulosclerosis (Bus et al., 2017). Additionally, APOC1 represents a novel diagnostic marker for clear cell renal cell carcinoma (Cui et al., 2020) and stimulates renal cell carcinoma through Wnt3a/STAT signaling (Li et al., 2020; Jiang et al., 2021). APOC1 is involved in breast cancer progression via the EMT and MAPK/JNK pathway (Zhang et al., 2022b). Furthermore, APOC1 is commonly associated with immune inflammation. Systematic pan-cancer analysis identifies APOC1 as an immunological biomarker that regulates macrophage polarization and promotes tumor metastasis (Ren et al., 2022). We revealed that APOC1 expression is elevated in IgAN serum, closely correlates with serum creatinine and eGFR, and is a novel diagnostic marker for IgAN (Figures 4–6).

More importantly, APOC1 was mainly expressed in renal tubular epithelial cells. We collected clinical data from 12 patients with IgAN nephropathy. Furthermore, we performed whole section scans of APOC1 immunohistochemistry and Masson staining results for these 12 kidney tissues. The original results of the whole section data scan are presented in the Supplementary Material. The results demonstrated that APOC1 was mainly expressed in IgA renal tubular epithelial cells. There may be some correlation between APOC1 expression and renal fibrosis.

Therefore, we selected HK-2 cells for *in vitro* cellular assays. Here, we demonstrated that the inhibition of APOC1 expression alleviates fibrosis in HK-2 cells. The NF-κB pathway activation can exacerbate renal fibrosis (Yang et al., 2019; Li et al., 2022c). Previous studies have demonstrated that the activation of the NF-κB signaling pathway can exacerbate renal fibrosis, whereas the inhibition of NF-κB signaling pathway activation can ameliorate renal injury (Moller-Hackbarth et al., 2021). In this research, we revealed that APOC1 can promote HK-2 cell fibrosis by activating the NF-κB signaling pathway (Figure 7). However, the exact mechanism of the APOC1 regulation of the NF-κB signaling pathway still requires more research to elucidate.

In conclusion, our research demonstrates that APOC1 may play an important role in the development of IgAN and as a novel potential molecular target for the diagnosis and treatment of IgAN. An early diagnosis of IgAN, leading to early prevention and treatment, may reduce the global burden of end-stage renal disease caused by IgAN. However, the role and mechanism of APOC1 in IgAN still require more basic and clinical studies to elucidate.

## Data Availability

The datasets presented in this study can be found in online repositories. The names of the repository/repositories and accession number(s) can be found in the article/Supplementary Material.
