# Supplementary figures and images for: APOC1 exacerbates renal fibrosis through the activation of the NF-κB signaling pathway in IgAN
Source: Front Pharmacol. 2023 May 25;14:1181435. doi: 10.3389/fphar.2023.1181435 (PMC10248024; doi:10.3389/fphar.2023.1181435)

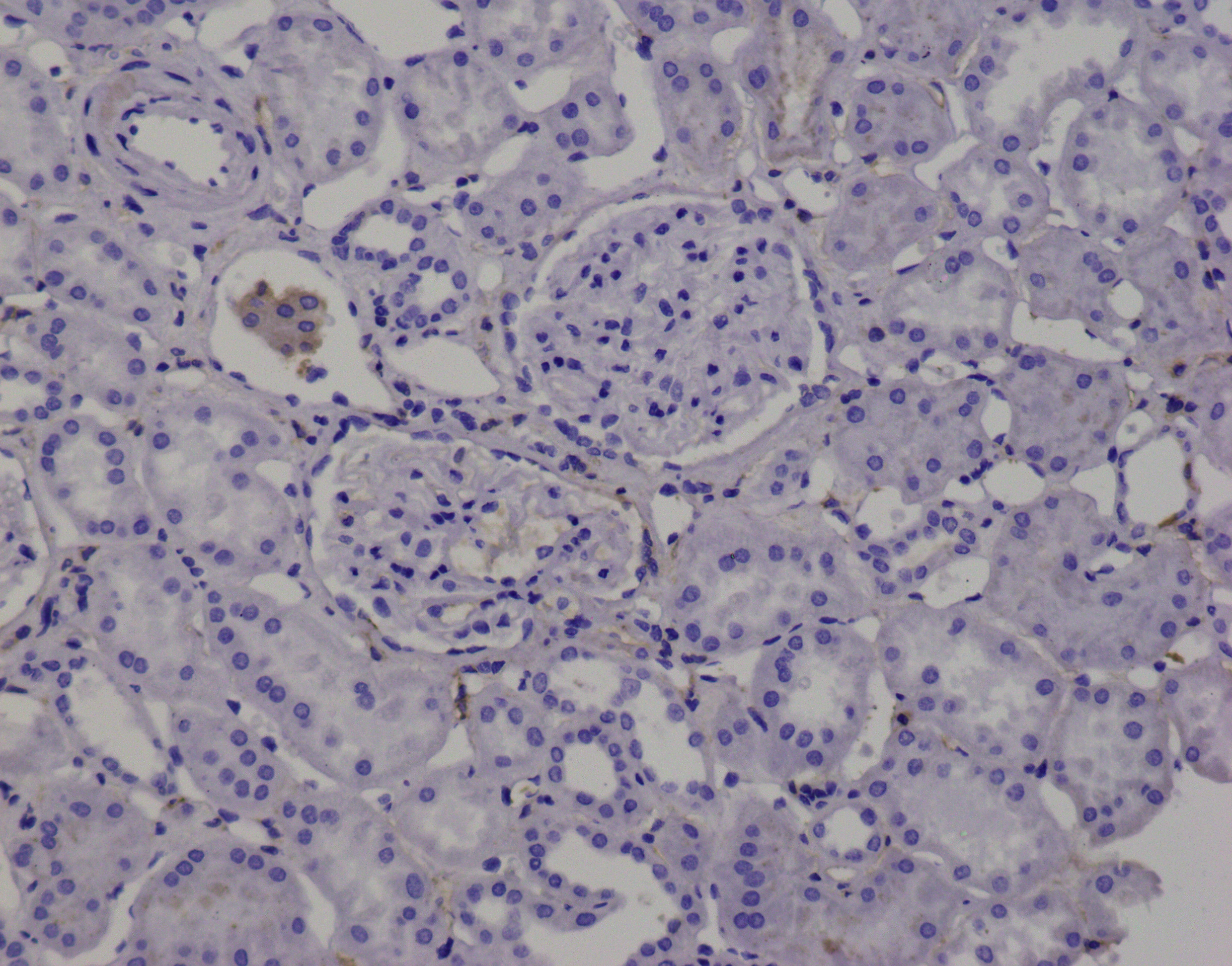

Supplement: Supplementary file 2 [file DataSheet3.ZIP › 2.IgAN patients IHF, APOC1 in IgAN/Ctrl ═╝╧±_29820.tif]

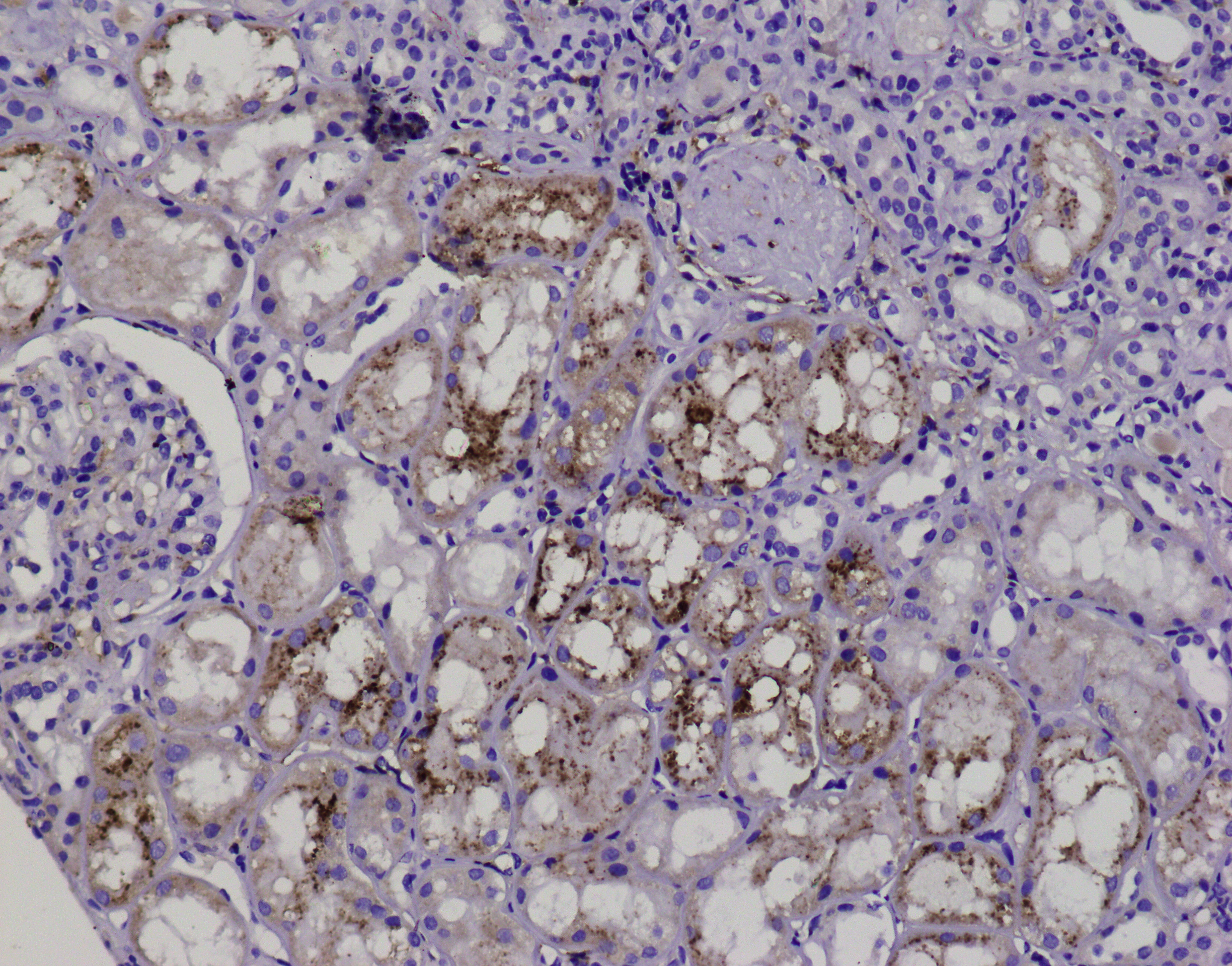

Supplement: Supplementary file 2 [file DataSheet3.ZIP › 2.IgAN patients IHF, APOC1 in IgAN/IgAN ═╝╧±_29815.tif]

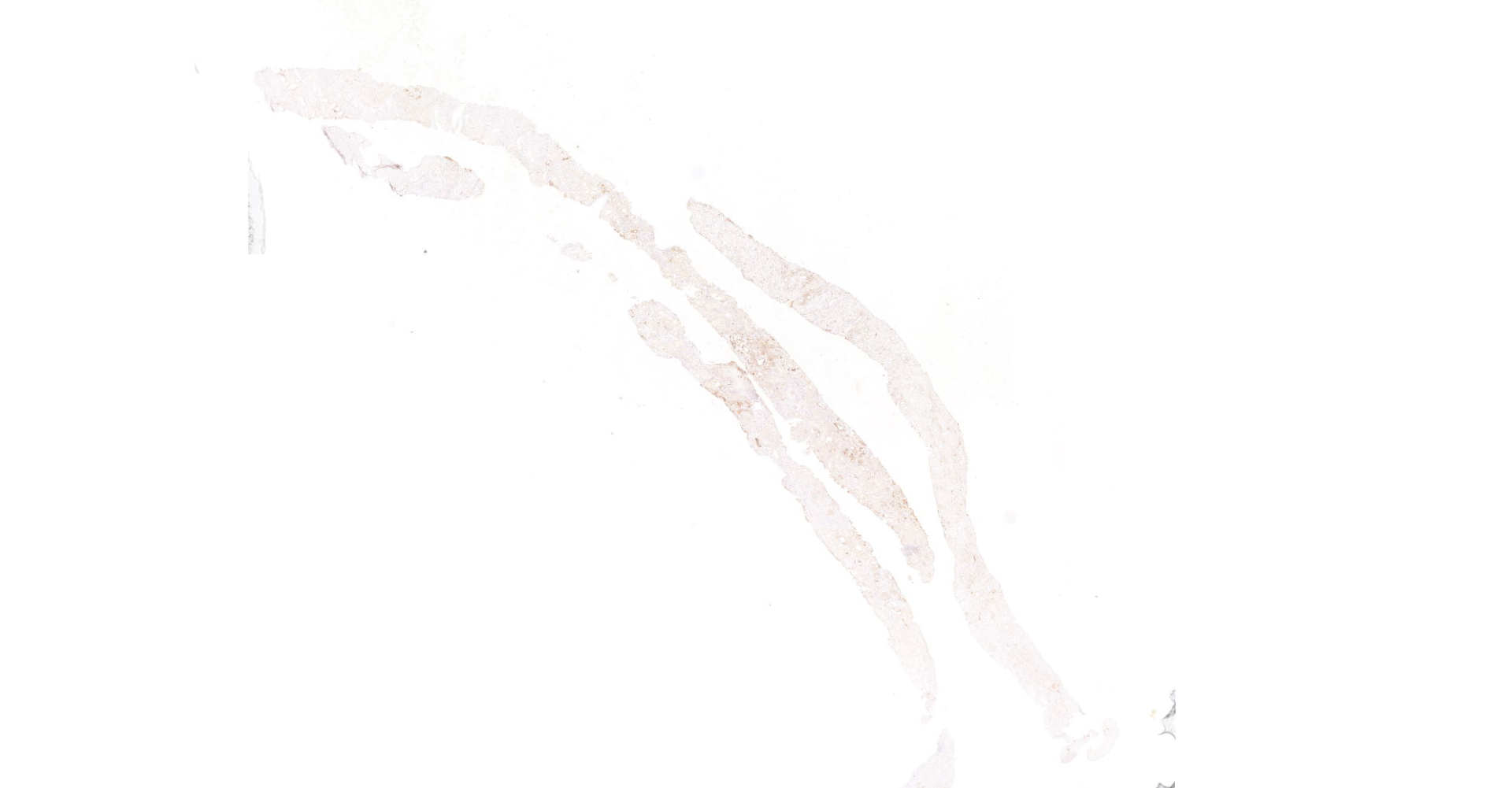

Supplement: Supplementary file 3 [file DataSheet11.ZIP › APOC1 IHC Full slice scan results/22-136_1.1x.tif]

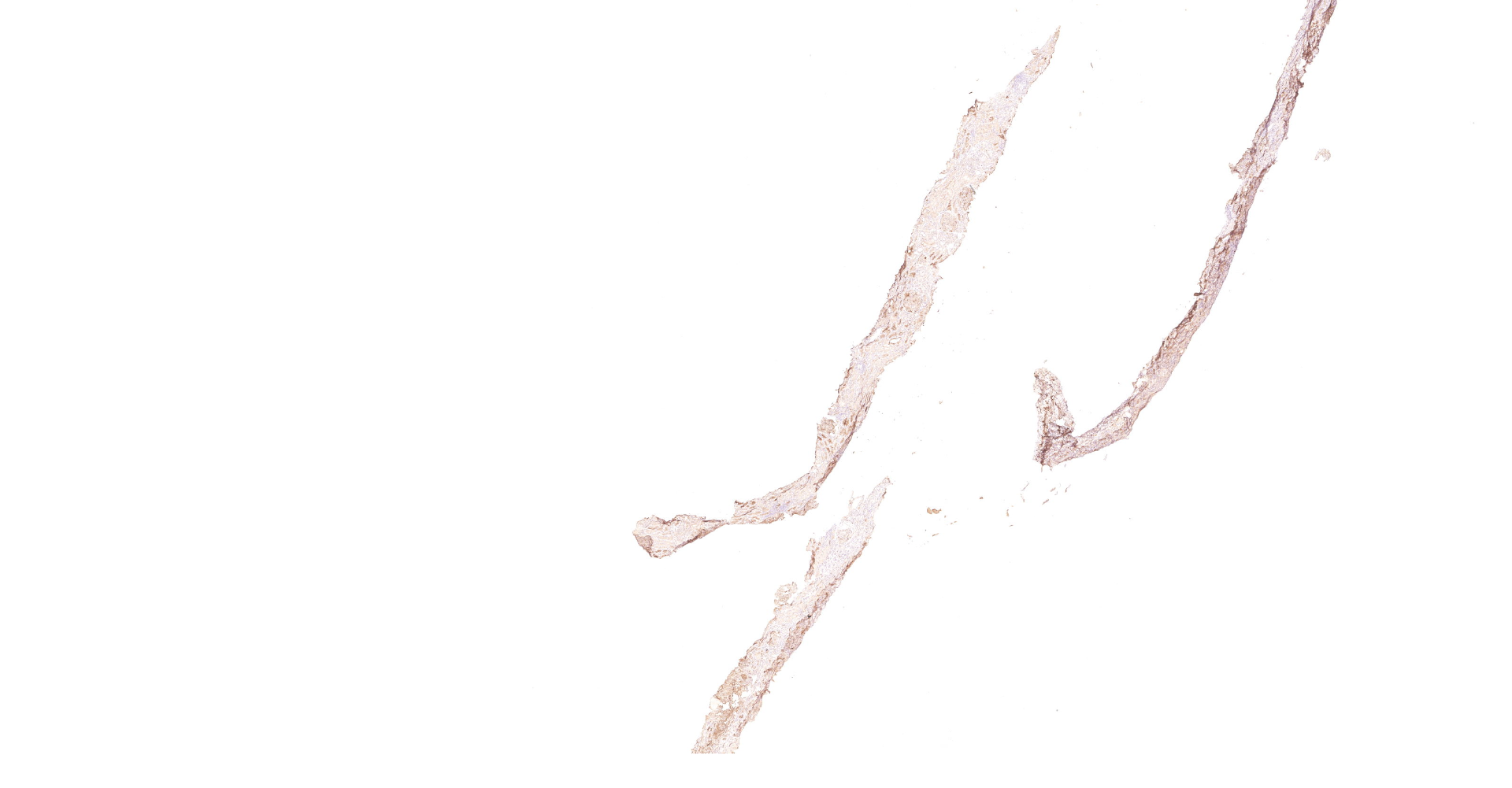

Supplement: Supplementary file 3 [file DataSheet11.ZIP › APOC1 IHC Full slice scan results/22-210_1.4x.tif]

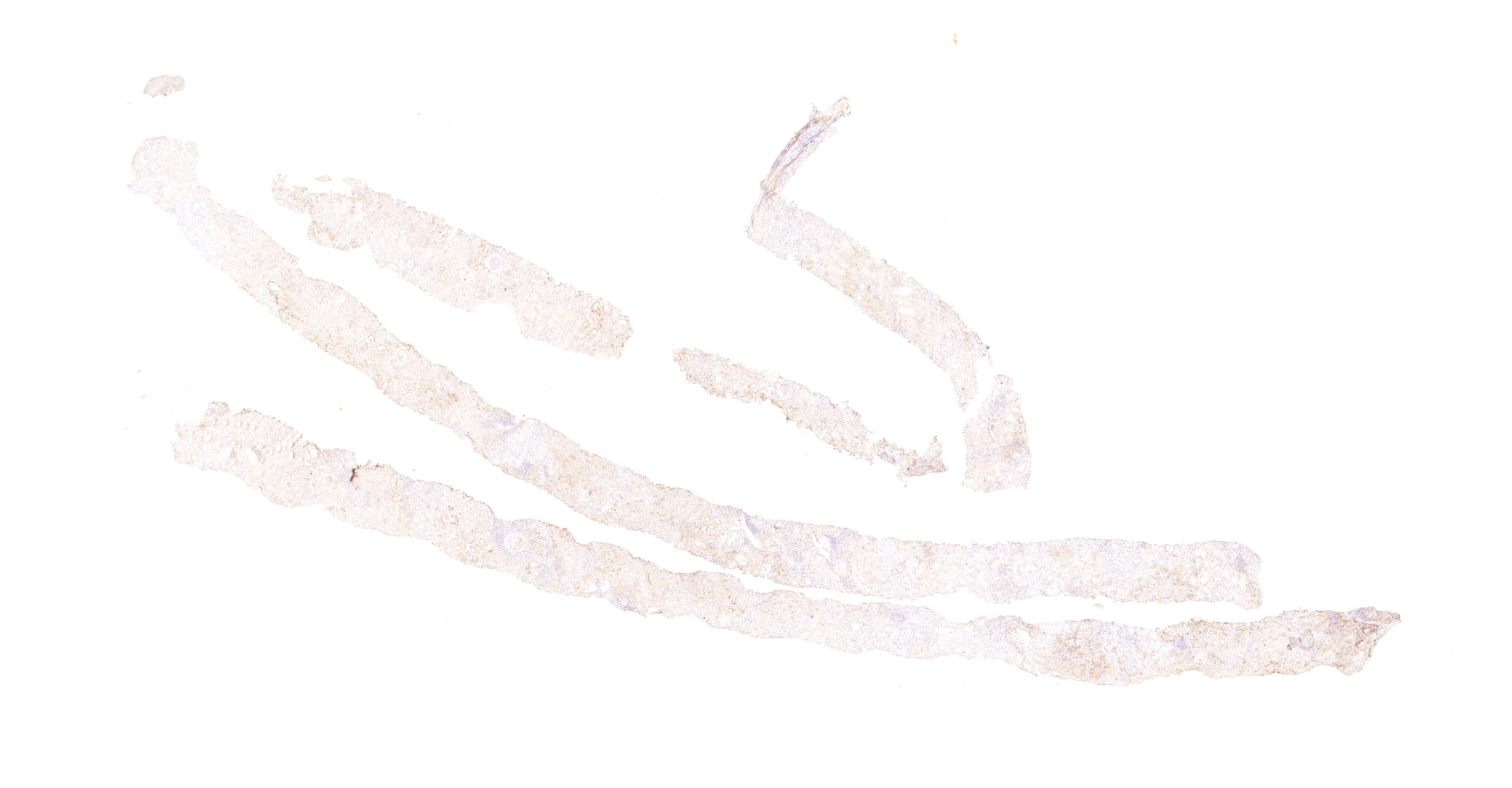

Supplement: Supplementary file 3 [file DataSheet11.ZIP › APOC1 IHC Full slice scan results/22-216_1.5x.tif]

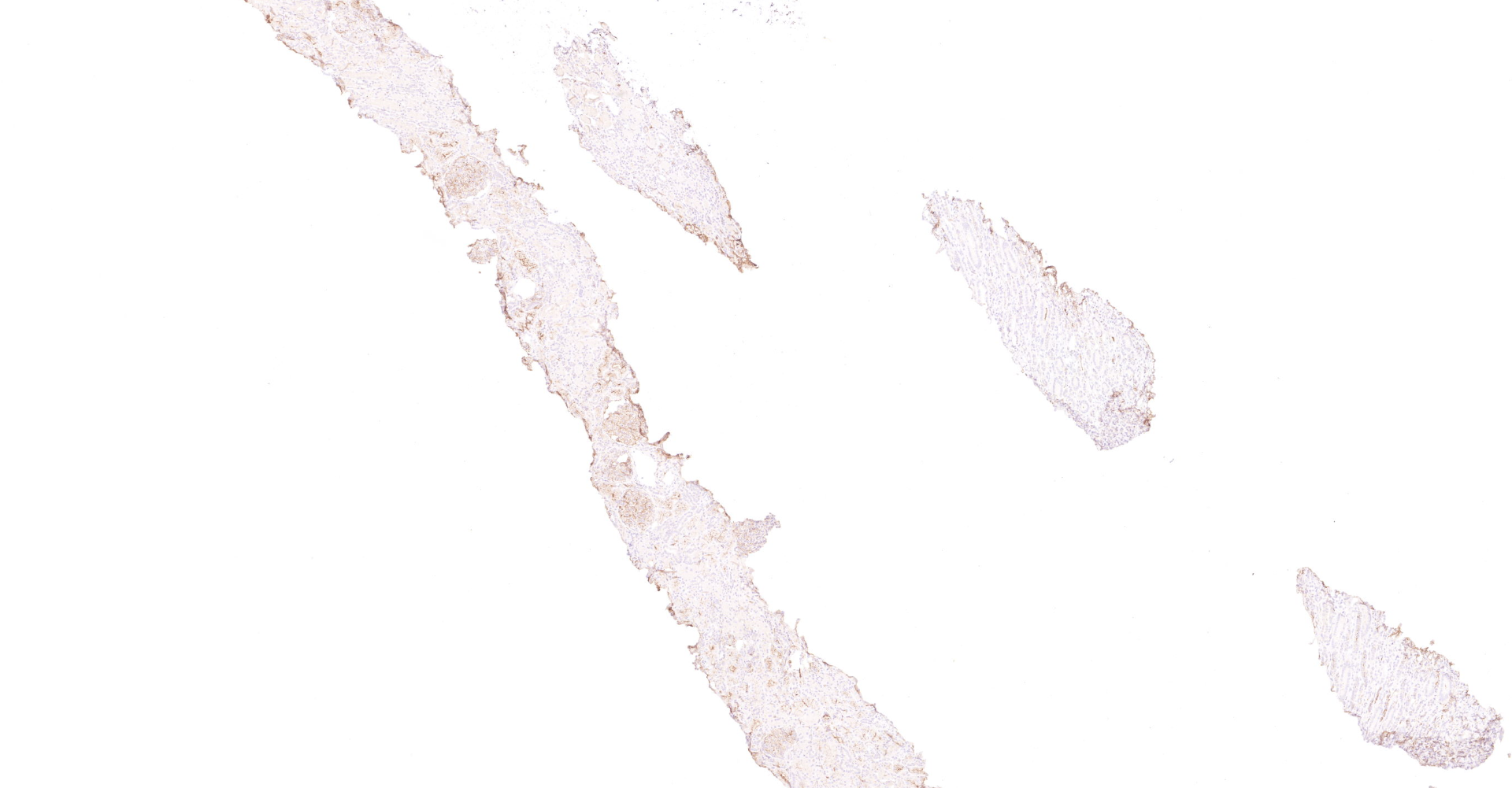

Supplement: Supplementary file 3 [file DataSheet11.ZIP › APOC1 IHC Full slice scan results/22-238_3.2x.tif]

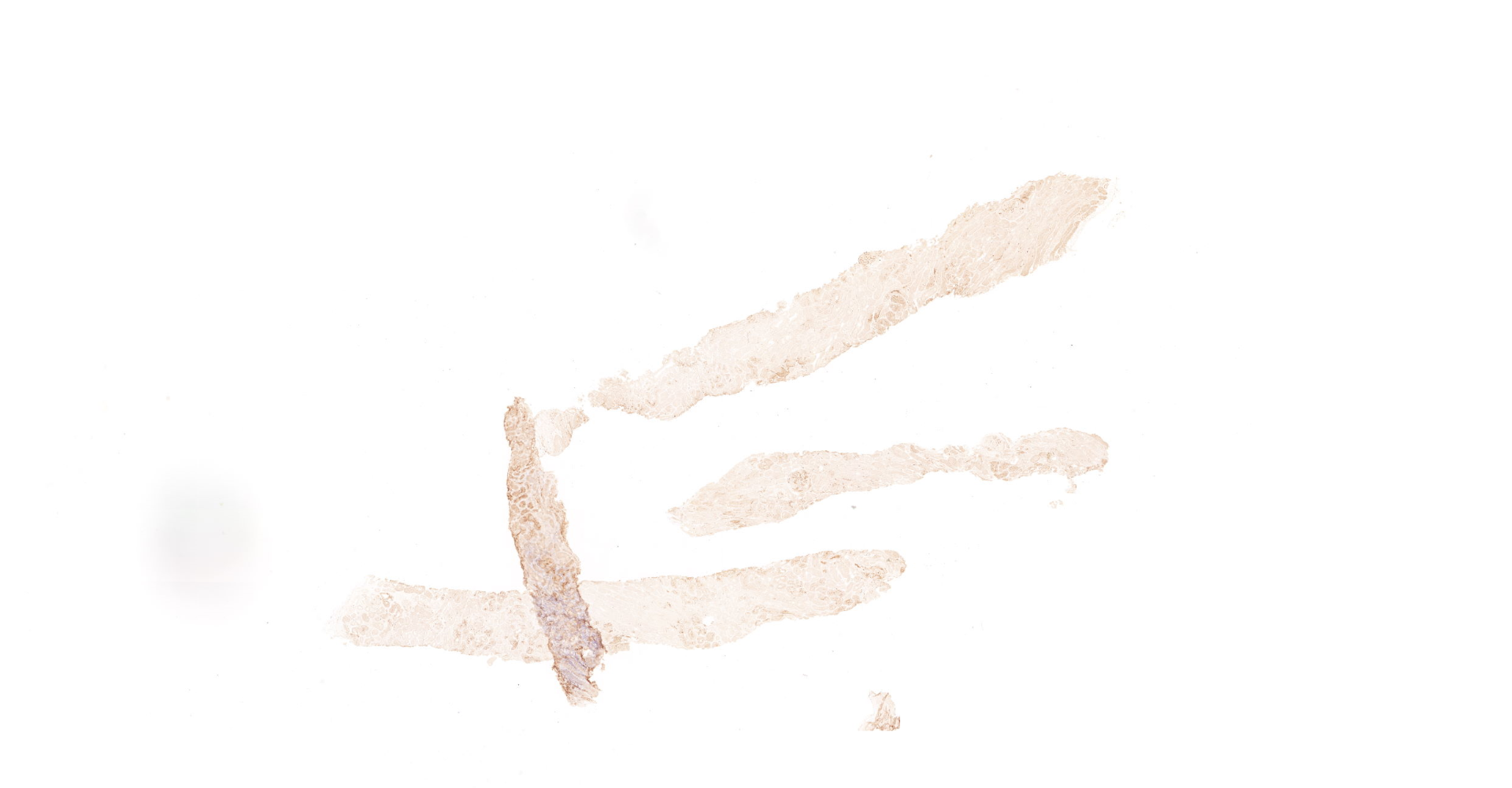

Supplement: Supplementary file 3 [file DataSheet11.ZIP › APOC1 IHC Full slice scan results/22-323_1.8x.tif]

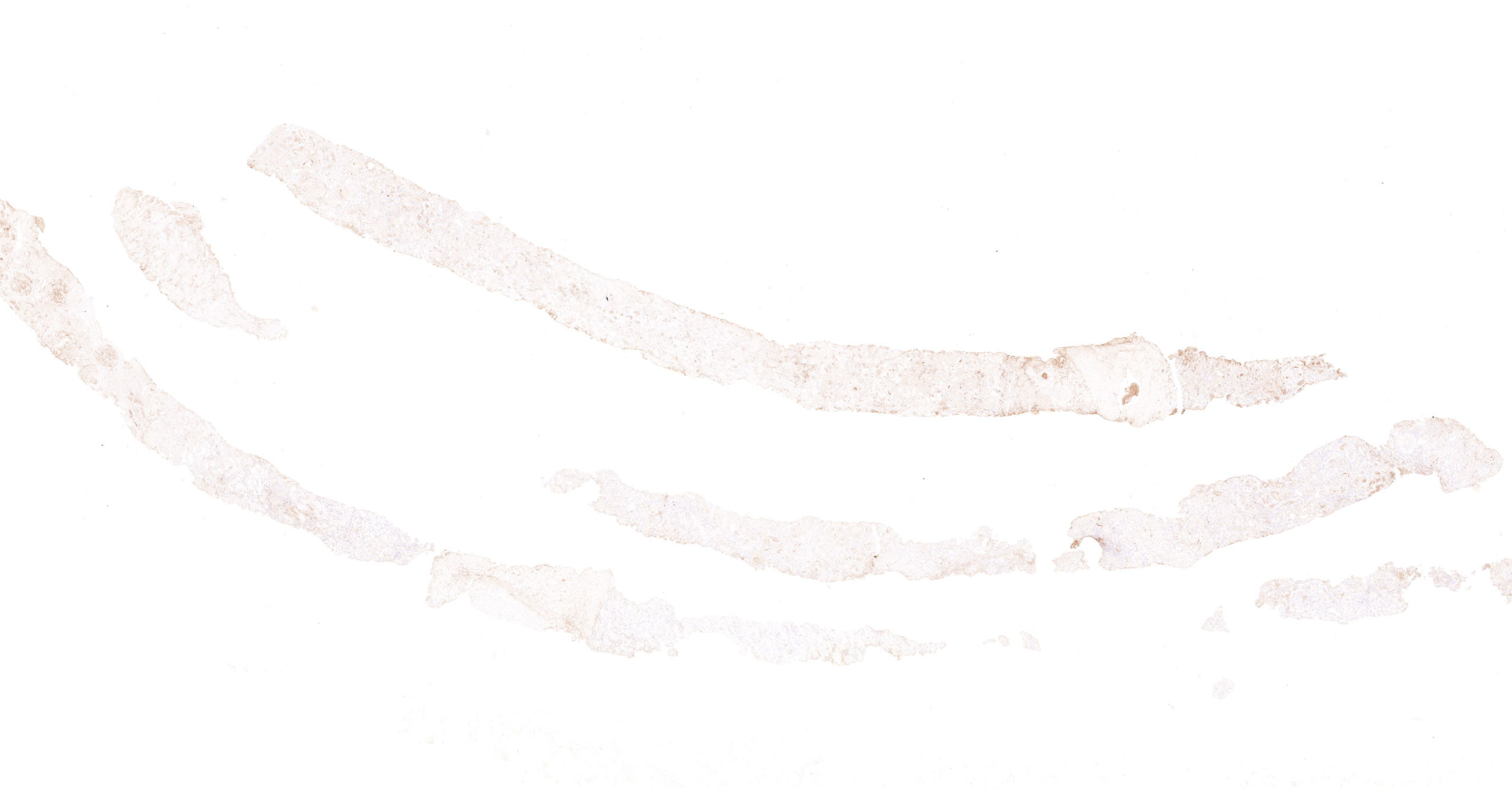

Supplement: Supplementary file 3 [file DataSheet11.ZIP › APOC1 IHC Full slice scan results/22-370_1.7x.tif]

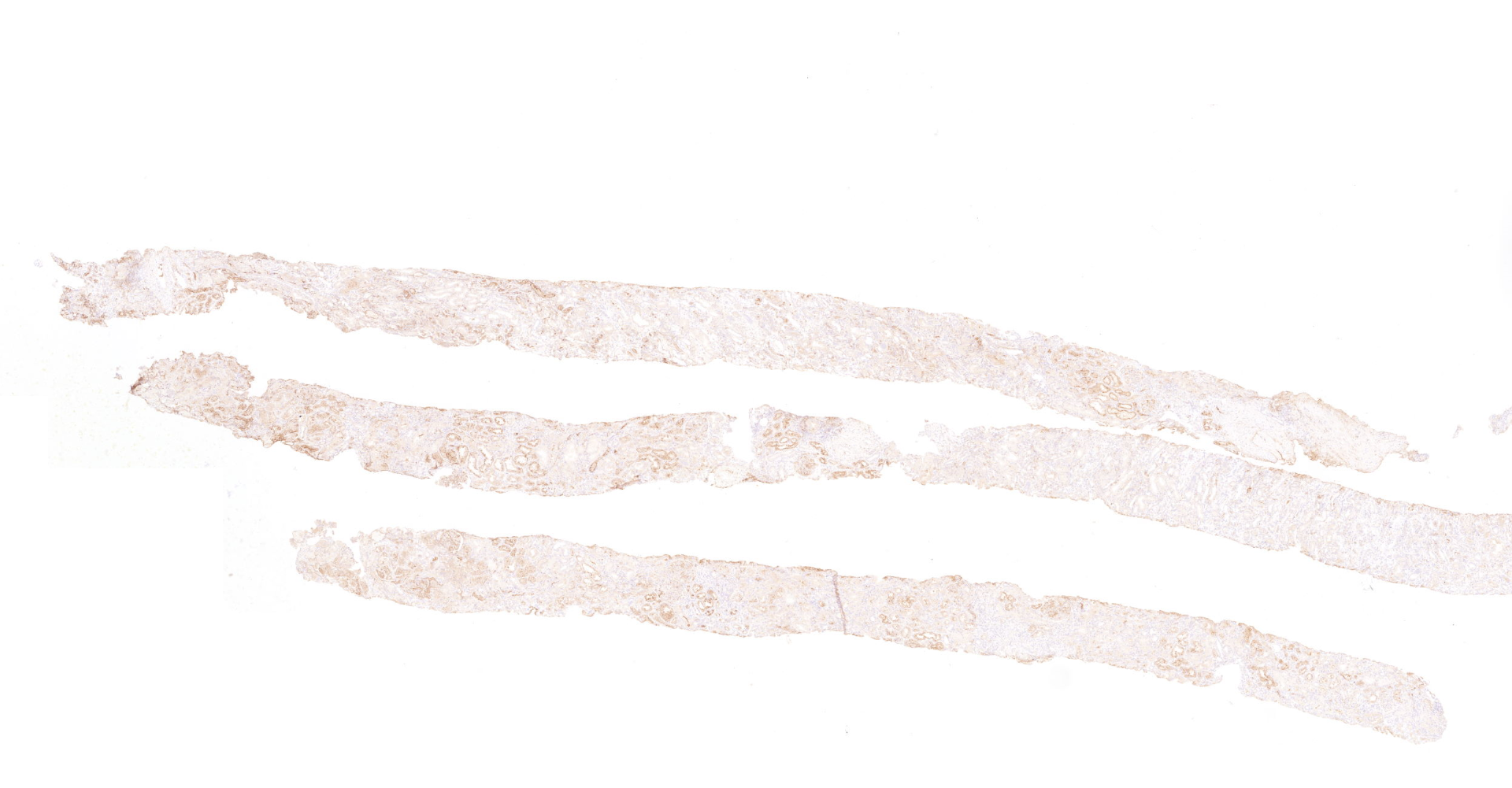

Supplement: Supplementary file 3 [file DataSheet11.ZIP › APOC1 IHC Full slice scan results/22-375_1.8x.tif]

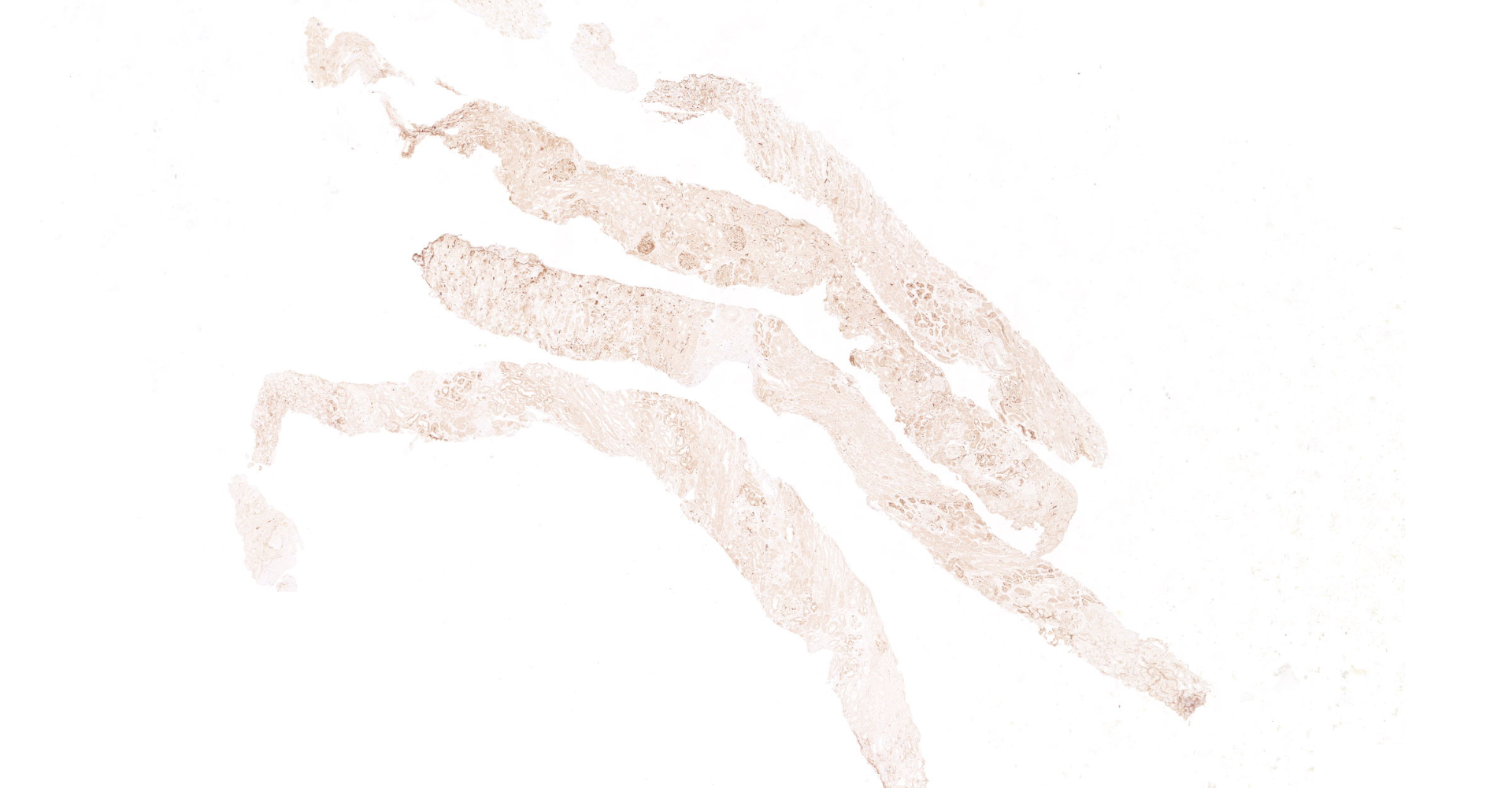

Supplement: Supplementary file 3 [file DataSheet11.ZIP › APOC1 IHC Full slice scan results/22-381_1.8x.tif]

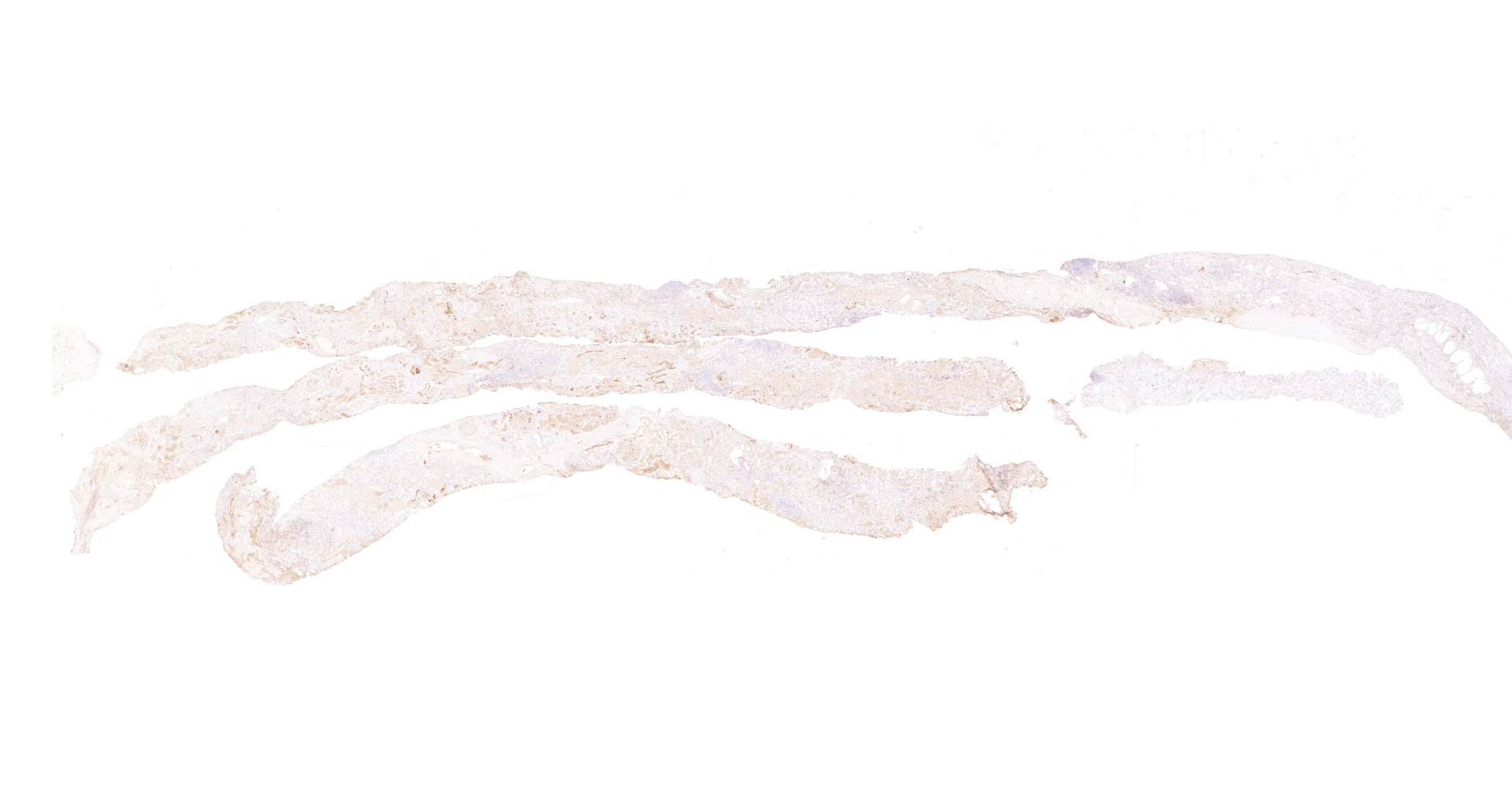

Supplement: Supplementary file 3 [file DataSheet11.ZIP › APOC1 IHC Full slice scan results/22-384_1.6x.tif]

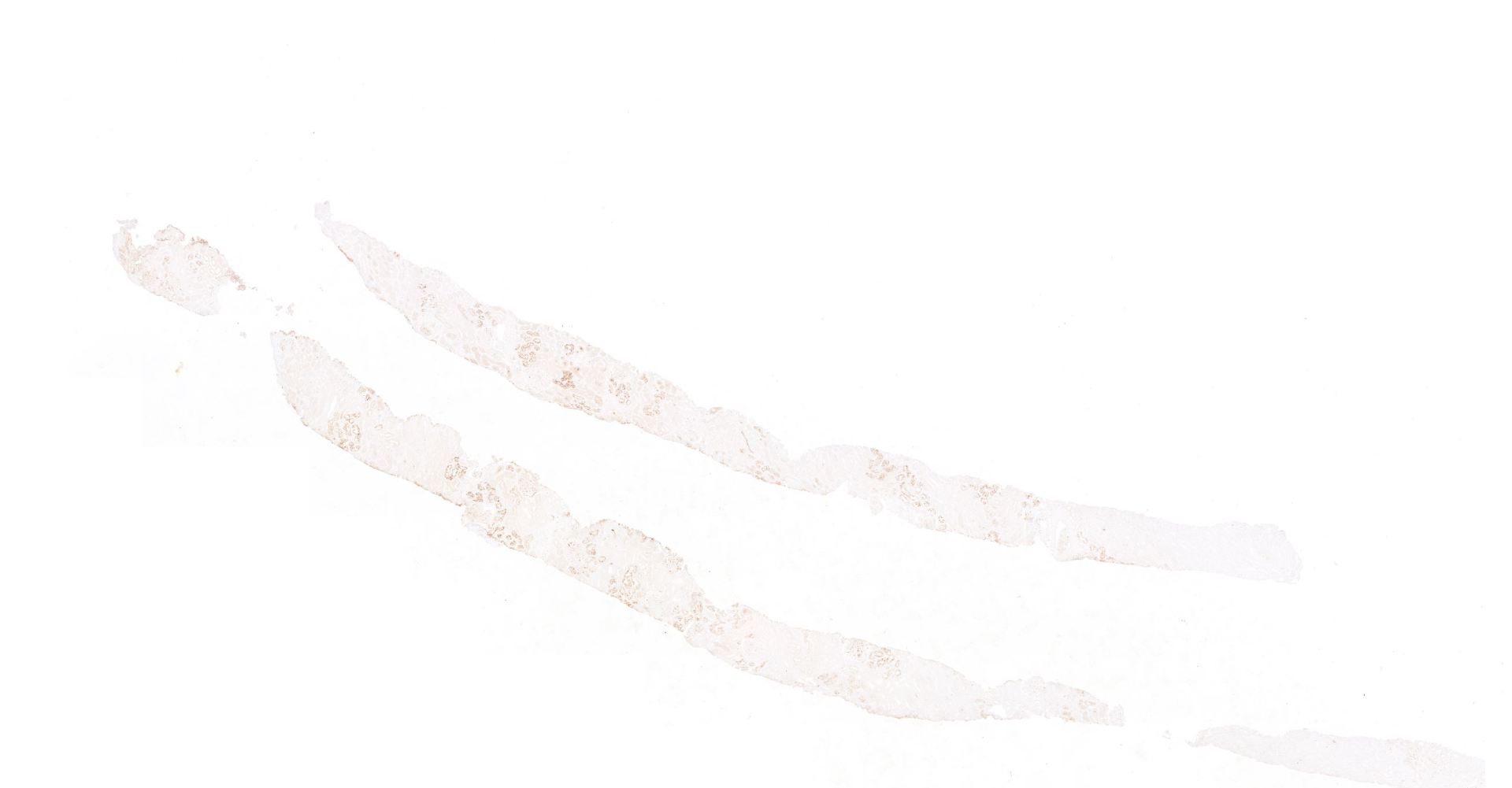

Supplement: Supplementary file 3 [file DataSheet11.ZIP › APOC1 IHC Full slice scan results/22-389_1.7x.tif]

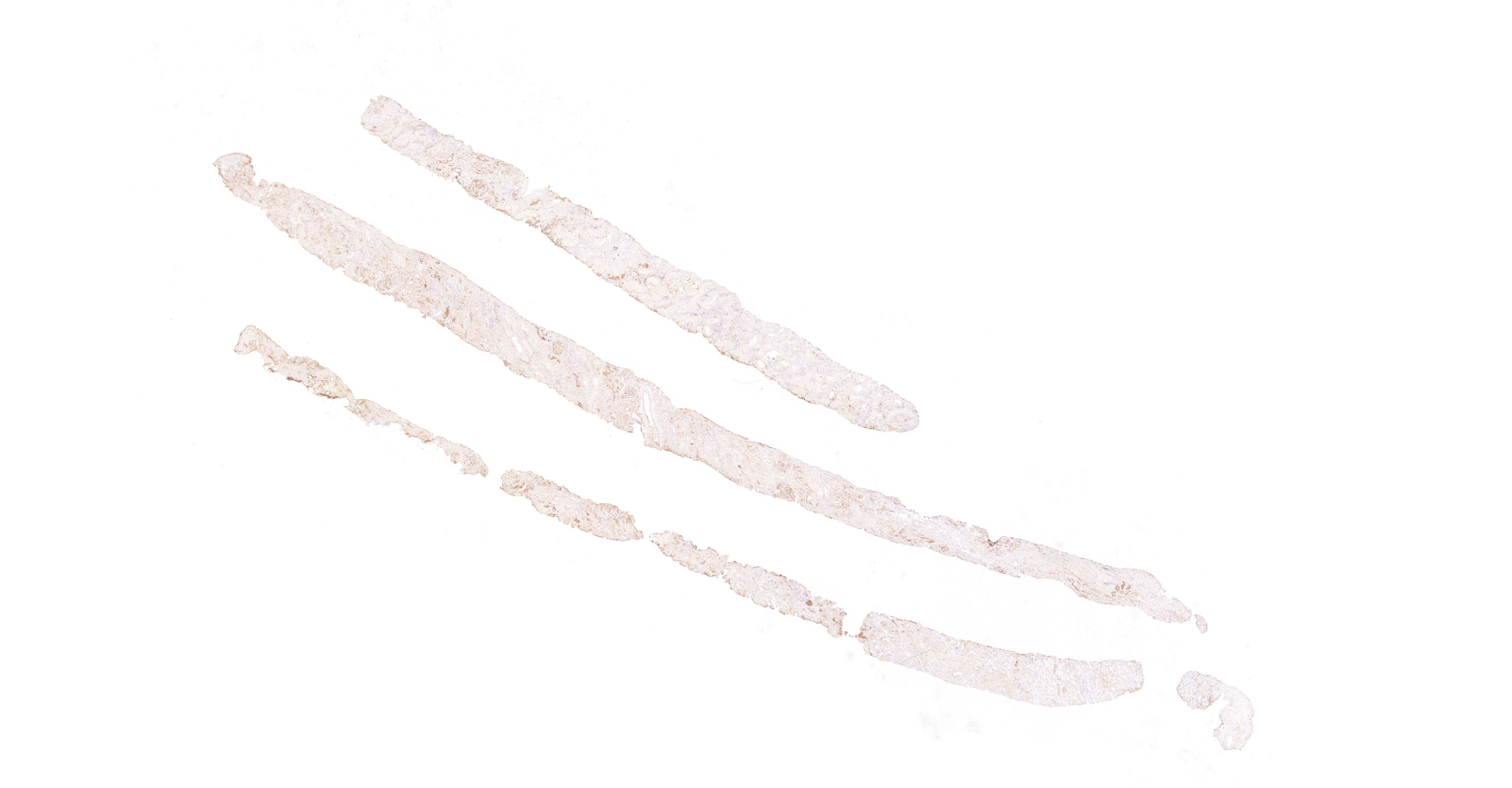

Supplement: Supplementary file 3 [file DataSheet11.ZIP › APOC1 IHC Full slice scan results/22-402_1.3x.tif]

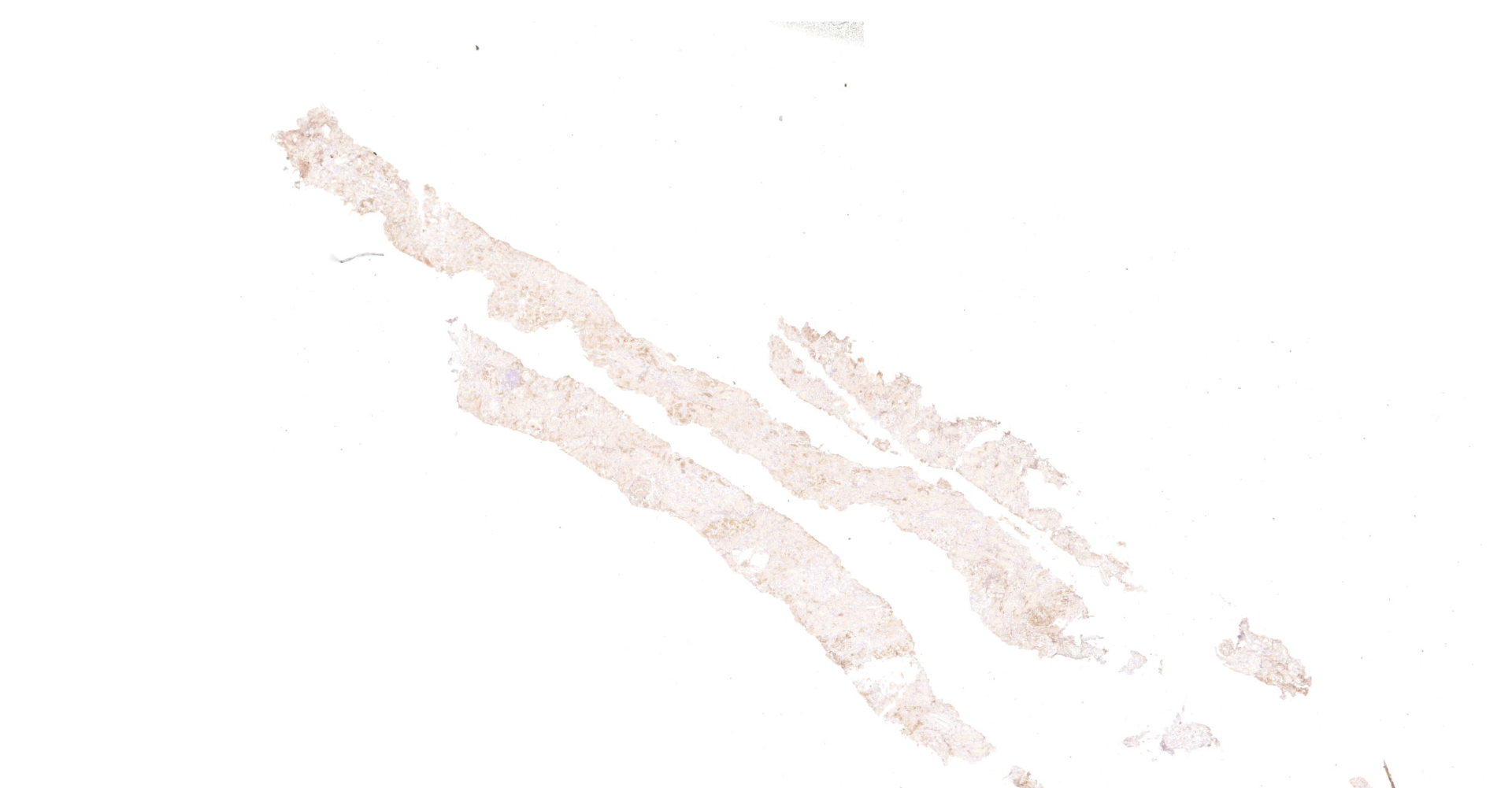

Supplement: Supplementary file 3 [file DataSheet11.ZIP › APOC1 IHC Full slice scan results/22-424_1.8x.tif]

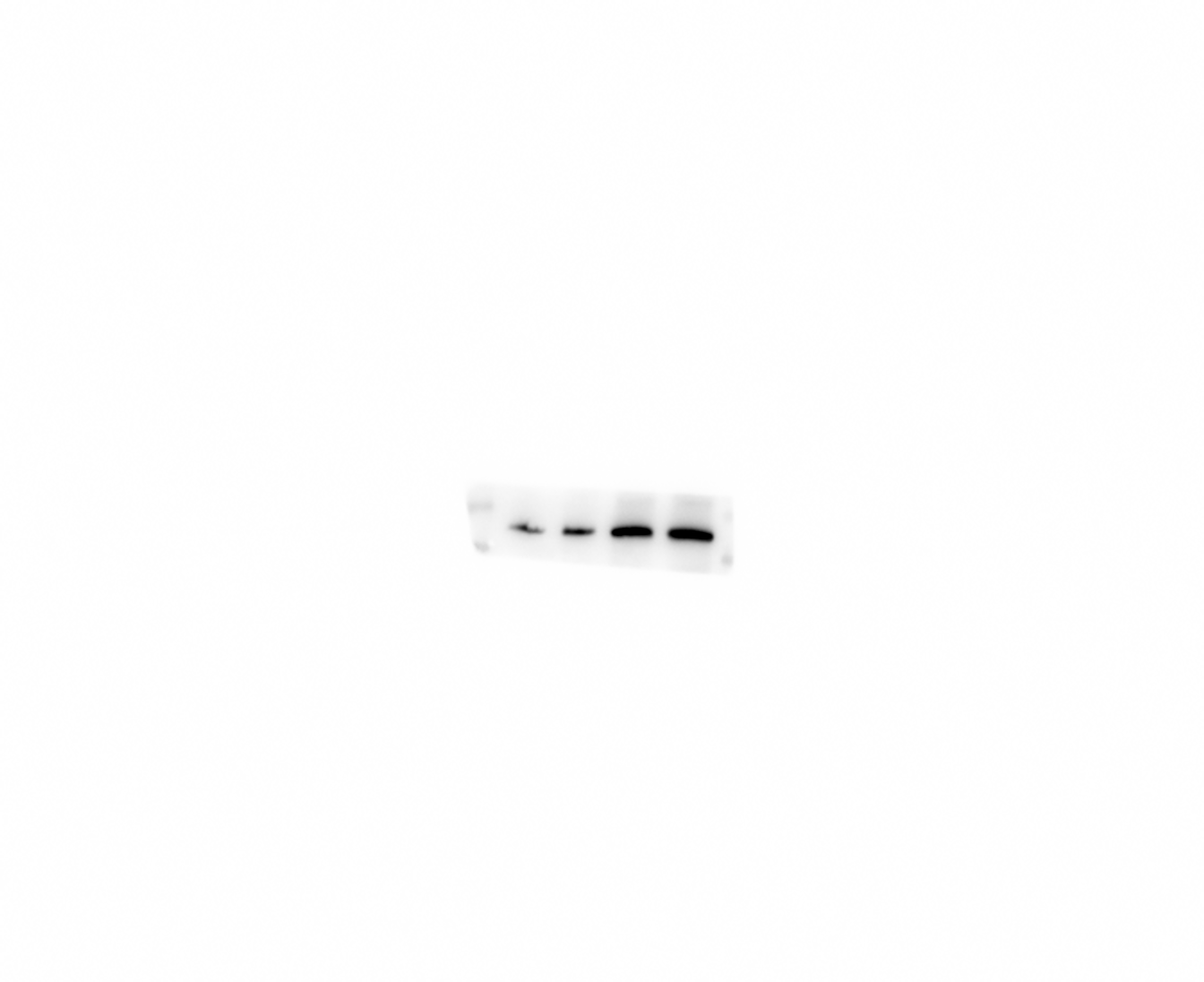

Supplement: Supplementary file 4 [file DataSheet8.ZIP › 7.WB OE-APOC1/APCO1 11-1.Tif]

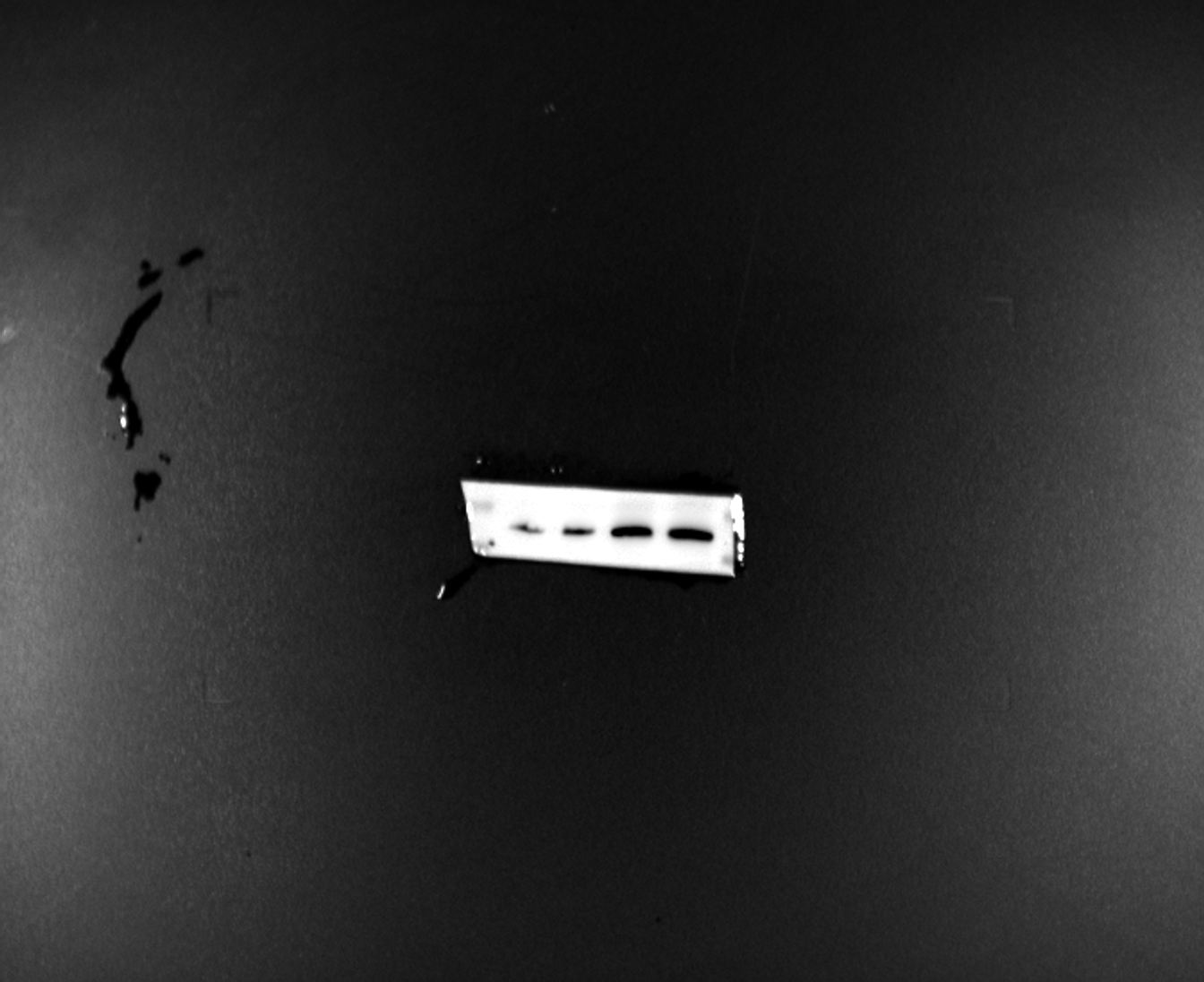

Supplement: Supplementary file 4 [file DataSheet8.ZIP › 7.WB OE-APOC1/APOC1 11-2.Tif]

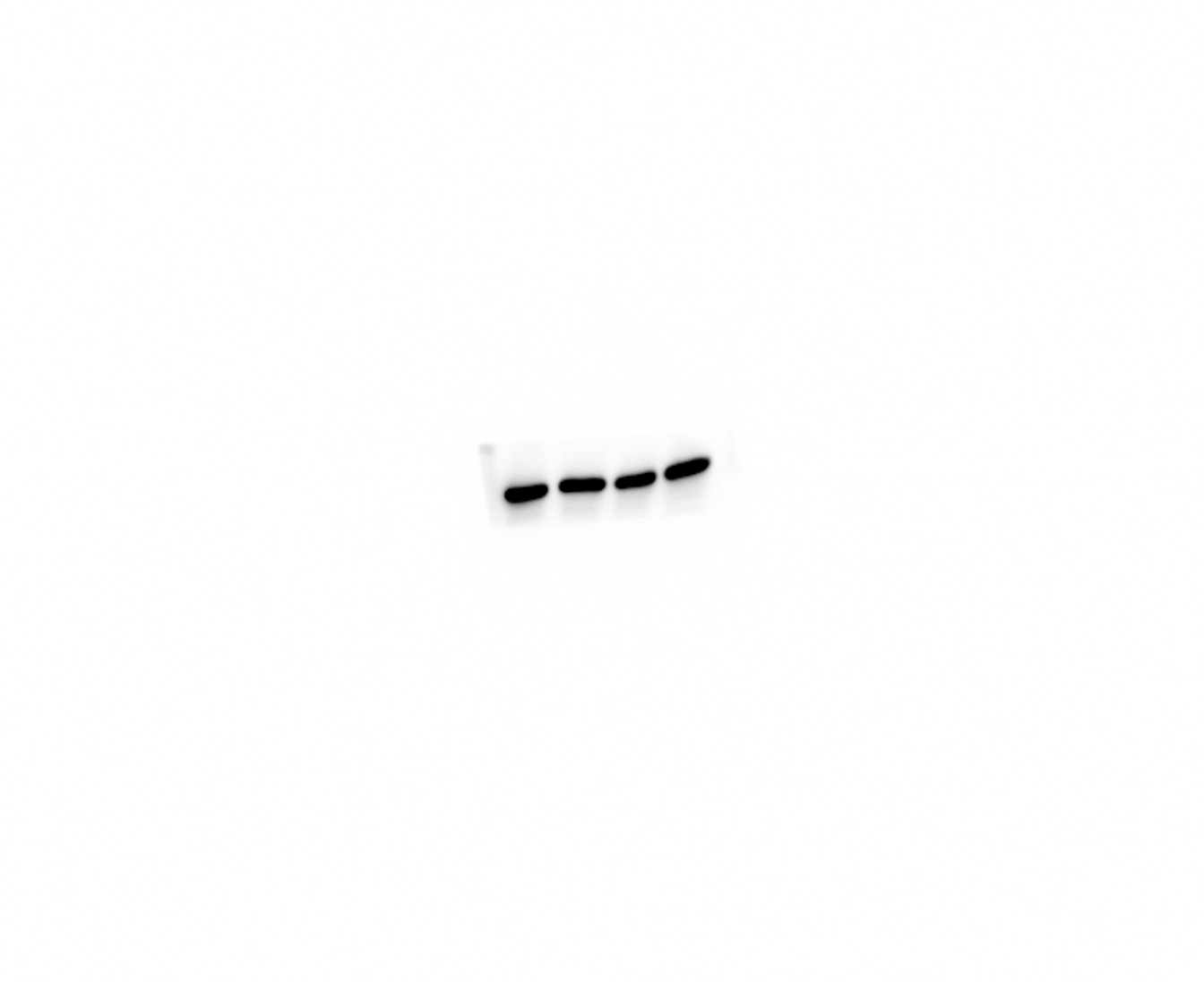

Supplement: Supplementary file 4 [file DataSheet8.ZIP › 7.WB OE-APOC1/GAPDH 2-1.Tif]

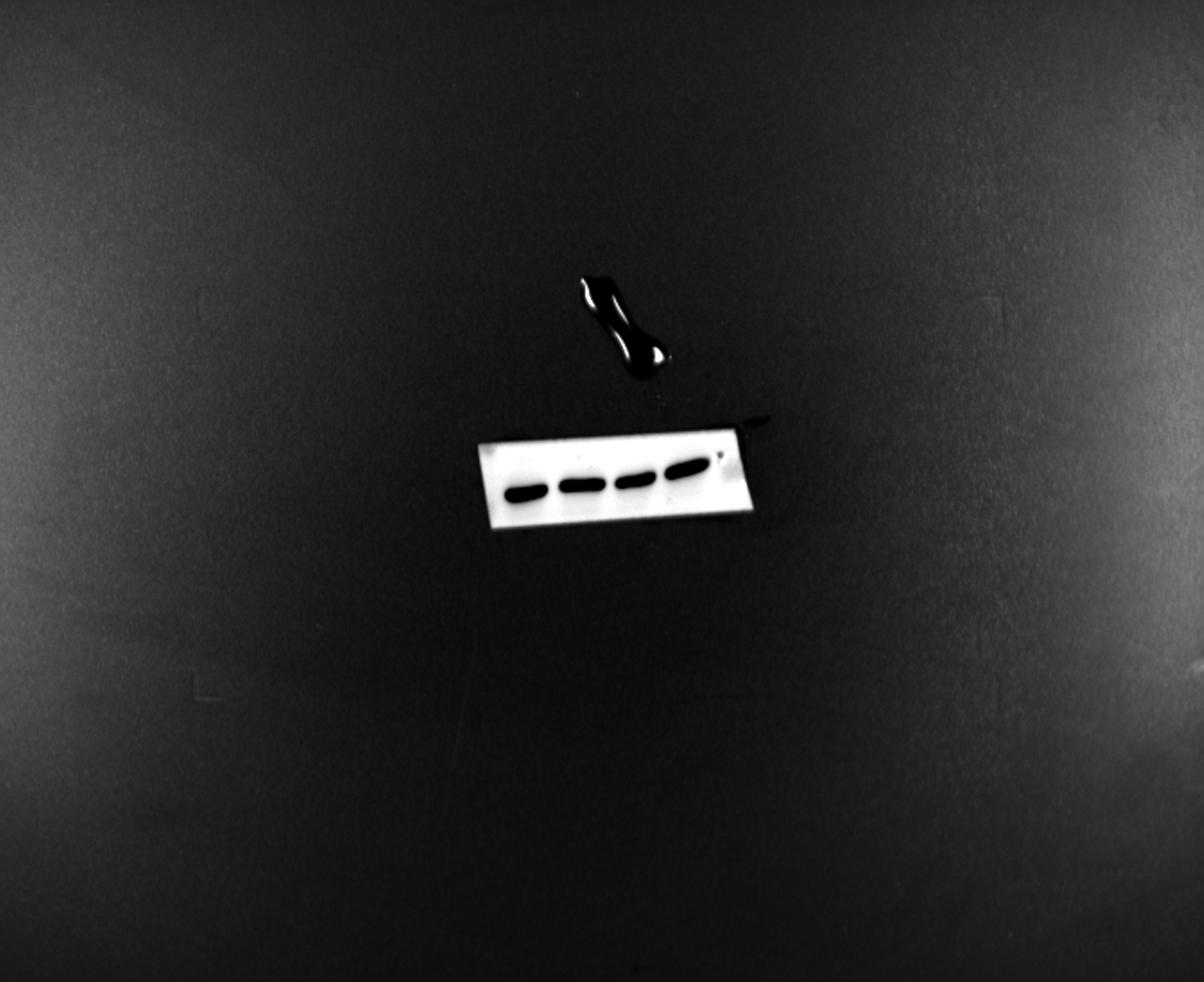

Supplement: Supplementary file 4 [file DataSheet8.ZIP › 7.WB OE-APOC1/GAPDH 2-2.Tif]

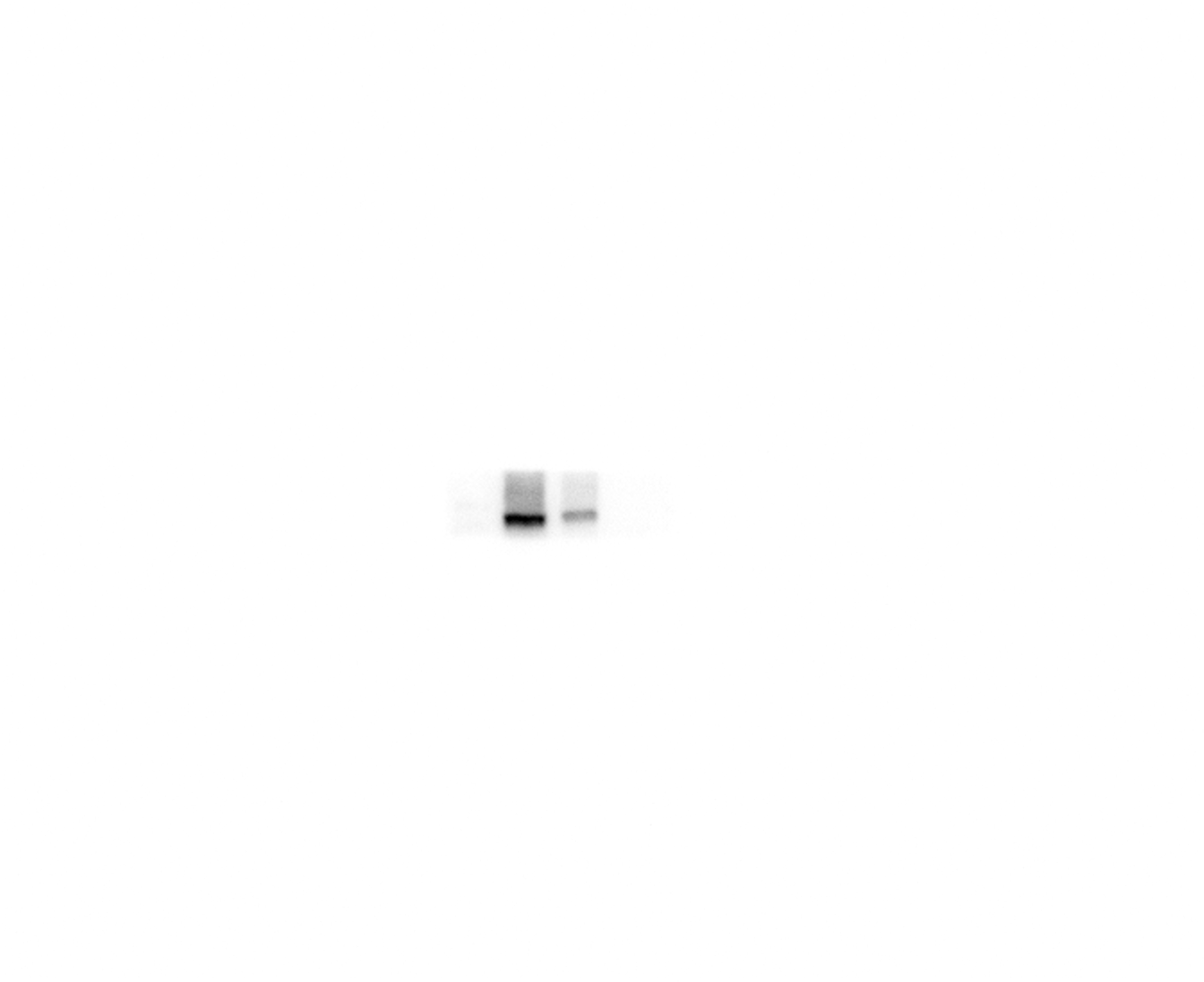

Supplement: Supplementary file 5 [file DataSheet9.ZIP › 8. WB si-APOC1 NF-a╩B/APOC136-1.Tif]

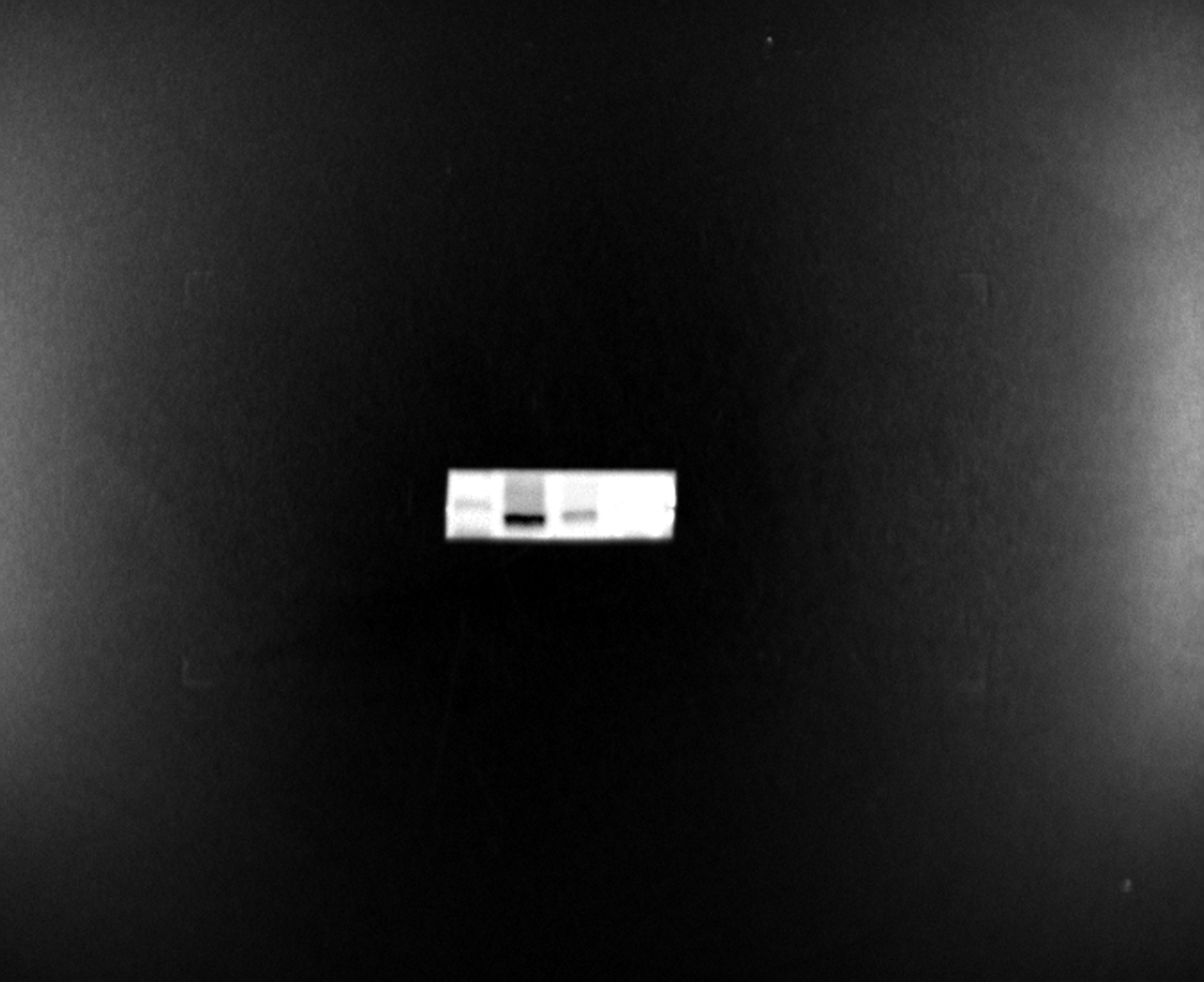

Supplement: Supplementary file 5 [file DataSheet9.ZIP › 8. WB si-APOC1 NF-a╩B/APOC136-2.Tif]

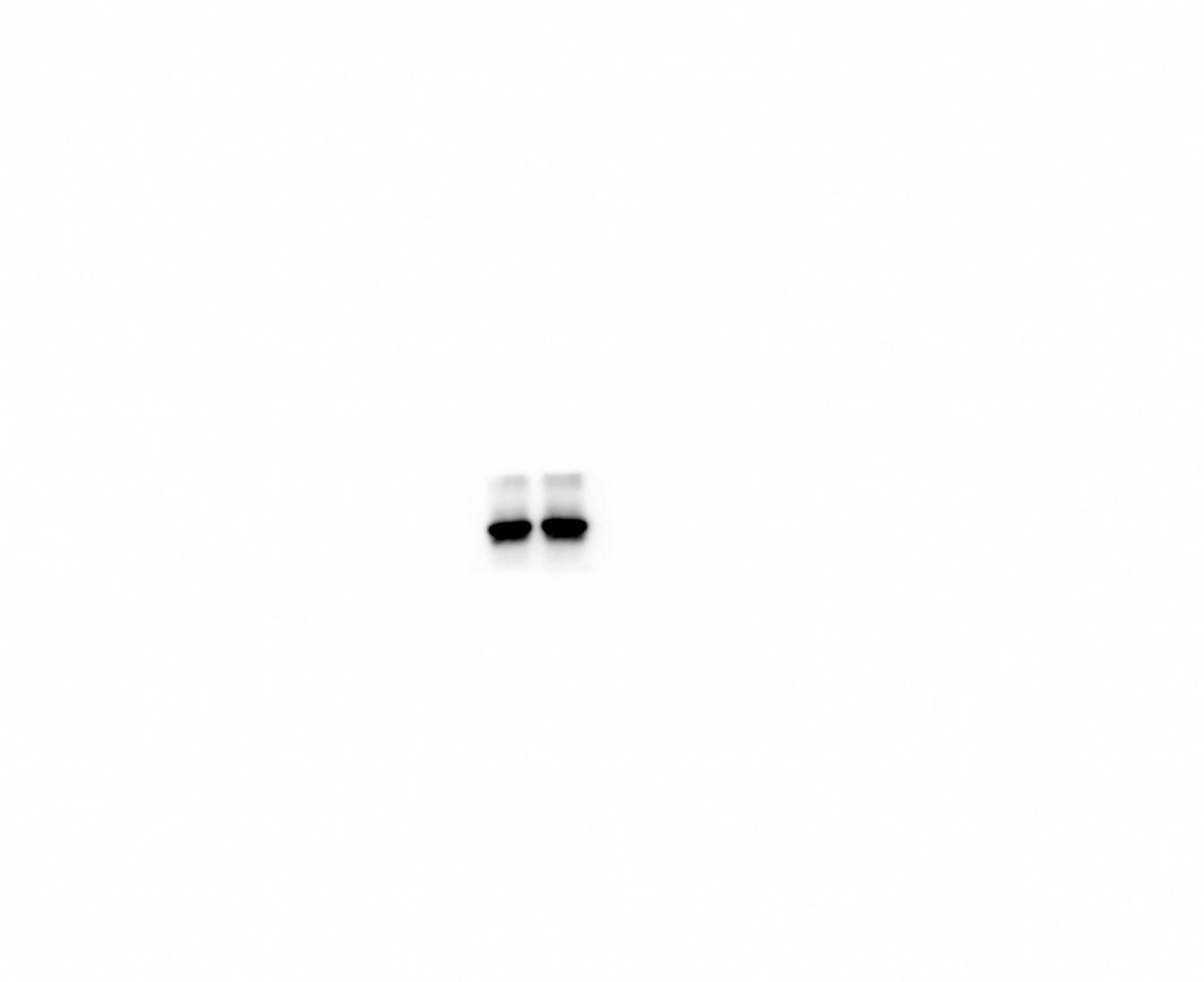

Supplement: Supplementary file 5 [file DataSheet9.ZIP › 8. WB si-APOC1 NF-a╩B/GAPDH12-1.Tif]

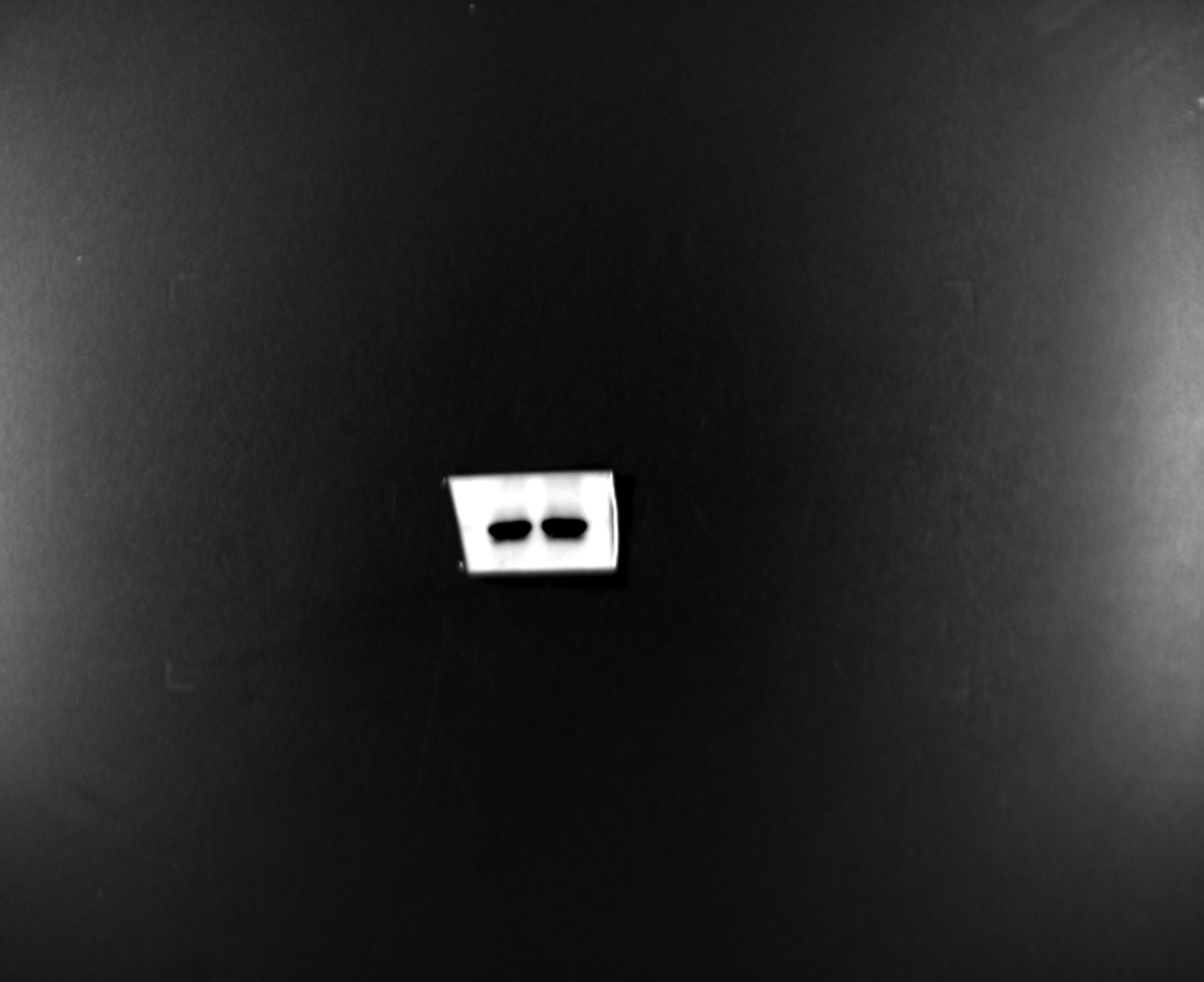

Supplement: Supplementary file 5 [file DataSheet9.ZIP › 8. WB si-APOC1 NF-a╩B/GAPDH12-2.Tif]

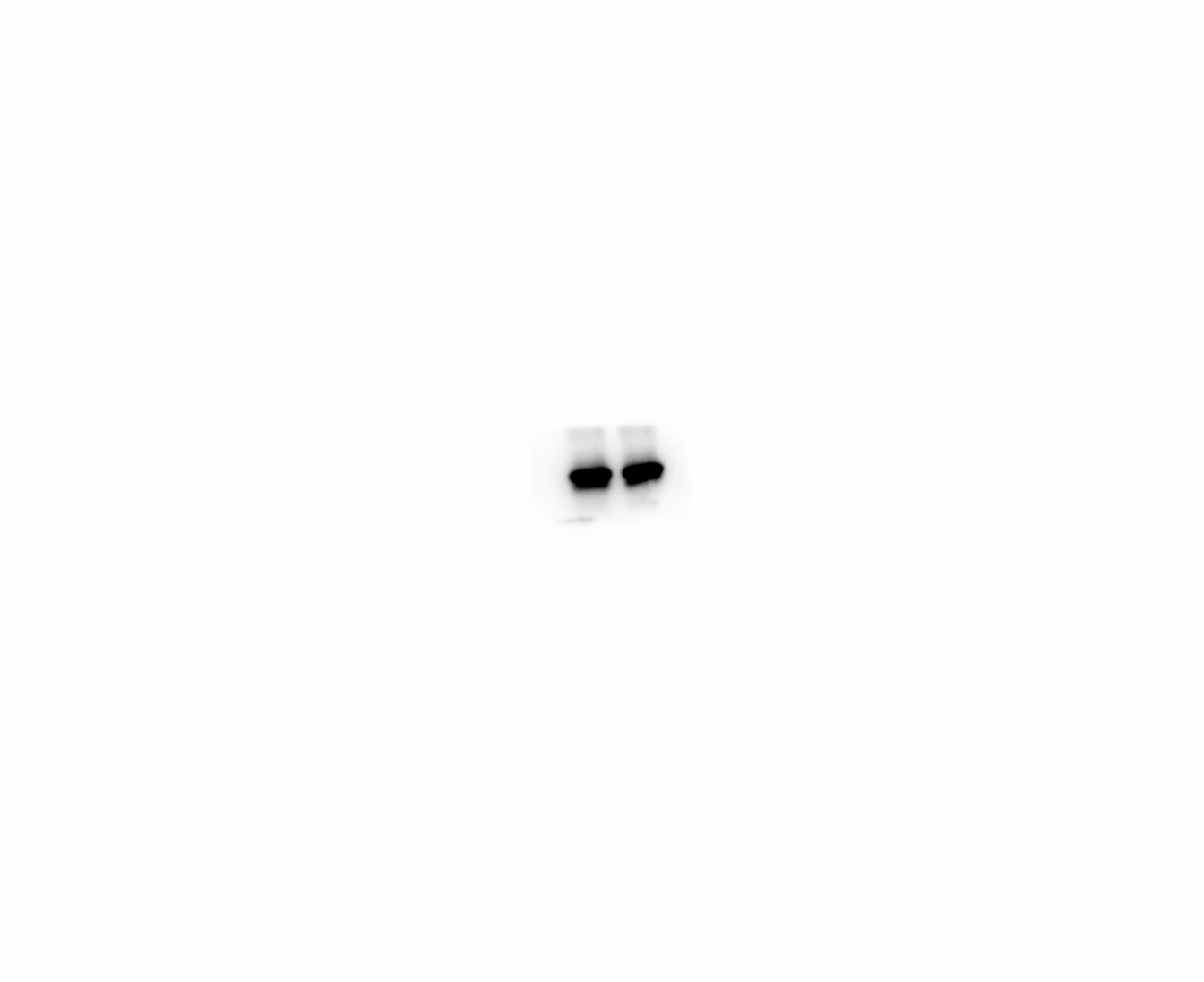

Supplement: Supplementary file 5 [file DataSheet9.ZIP › 8. WB si-APOC1 NF-a╩B/NFKB10-1.Tif]

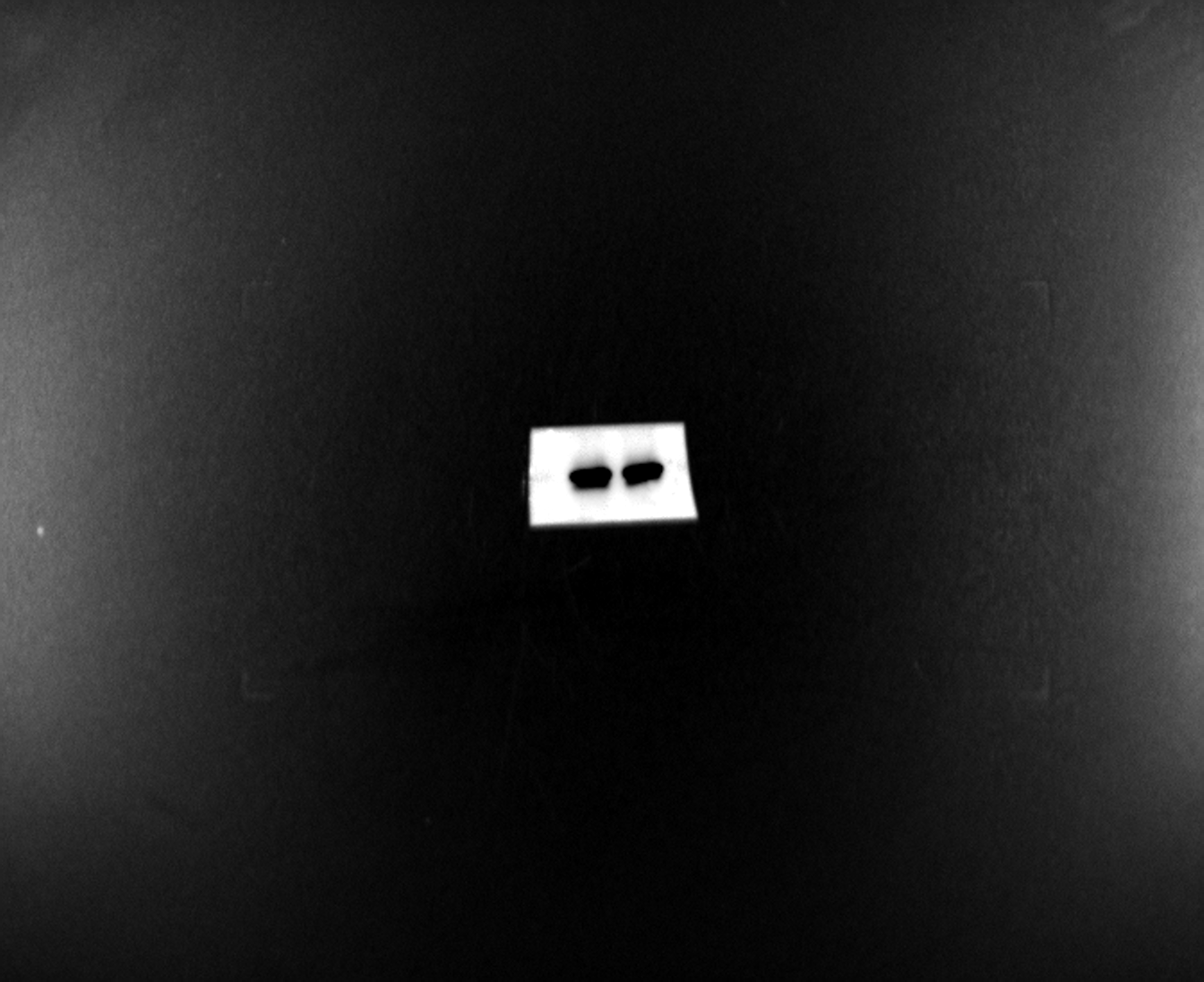

Supplement: Supplementary file 5 [file DataSheet9.ZIP › 8. WB si-APOC1 NF-a╩B/NFKB10-2.Tif]

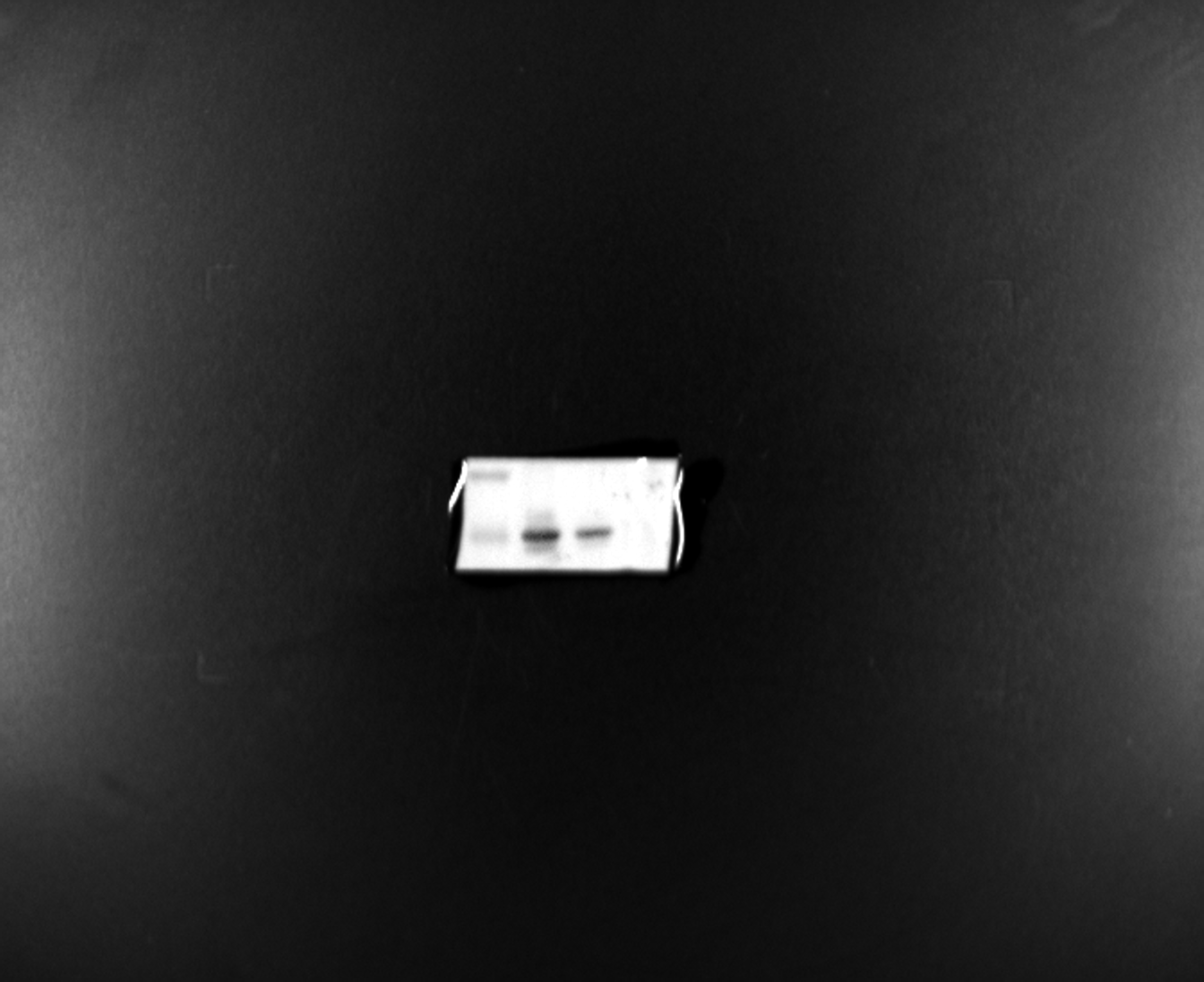

Supplement: Supplementary file 5 [file DataSheet9.ZIP › 8. WB si-APOC1 NF-a╩B/PNFKB5-2.Tif]

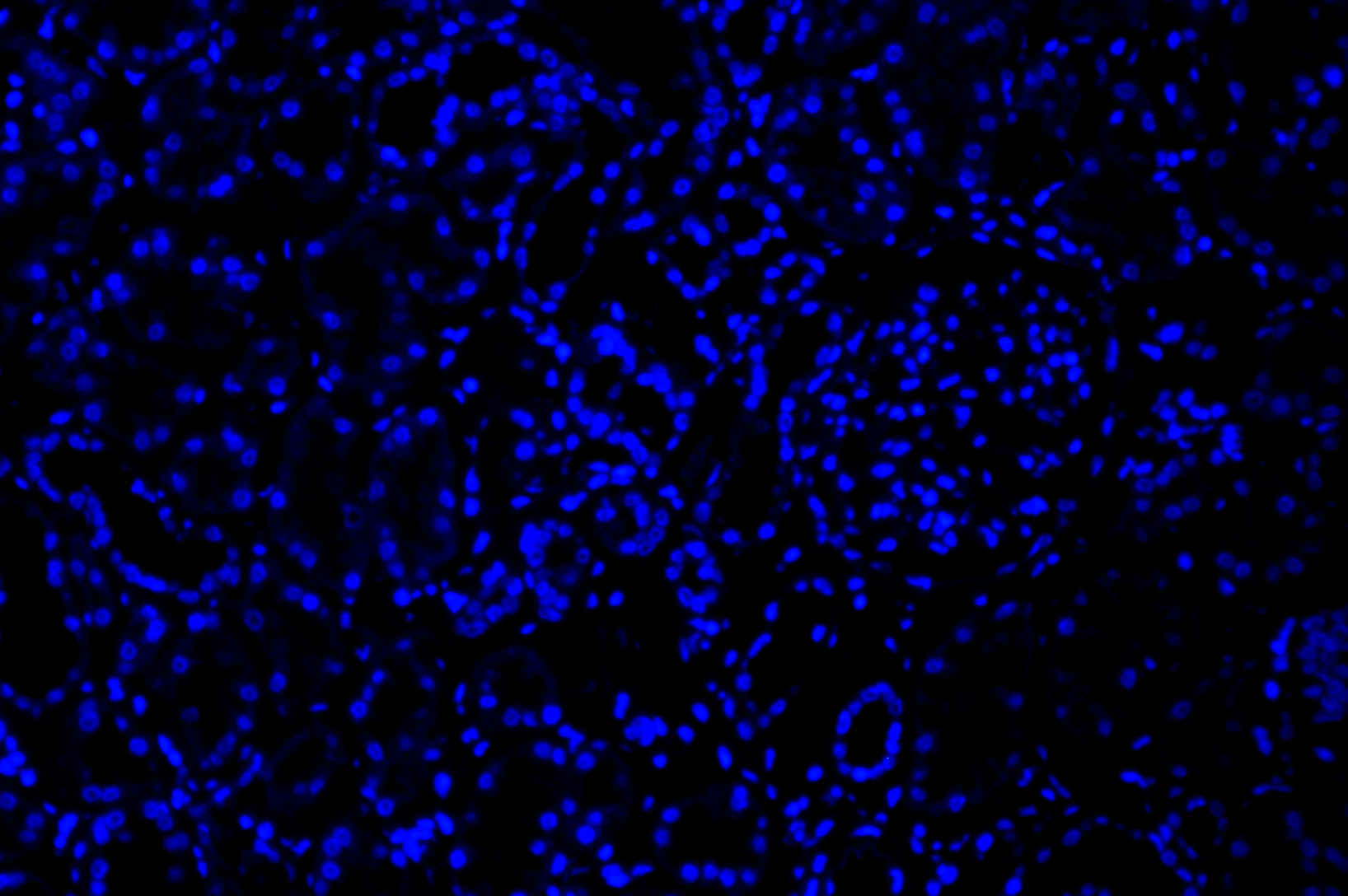

Supplement: Supplementary file 10 [file DataSheet4.ZIP › 3. IgAN patients IF,APOC1 in IgAN/Ctrl/DAPI.tif]

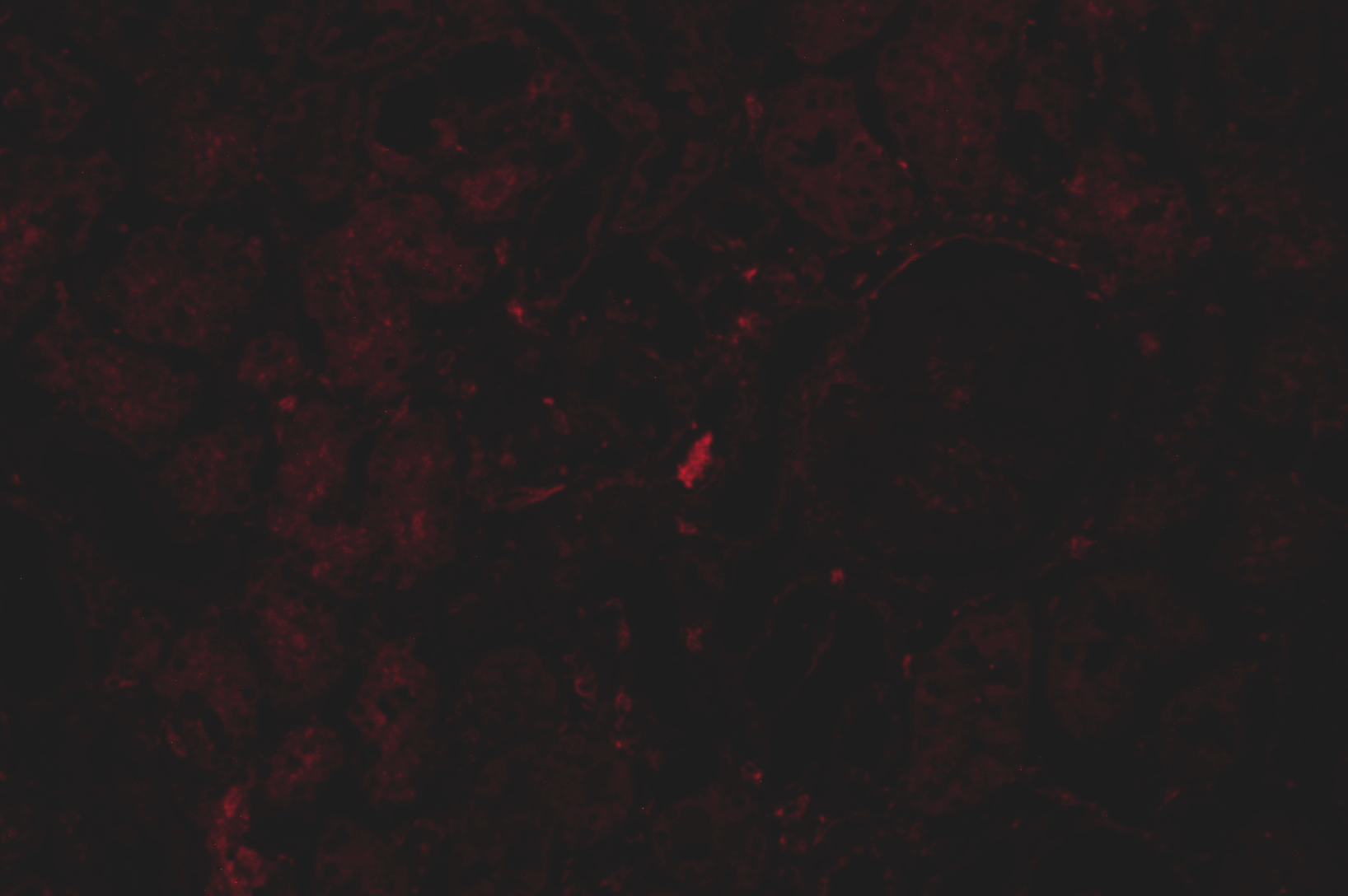

Supplement: Supplementary file 10 [file DataSheet4.ZIP › 3. IgAN patients IF,APOC1 in IgAN/Ctrl/Red.tif]

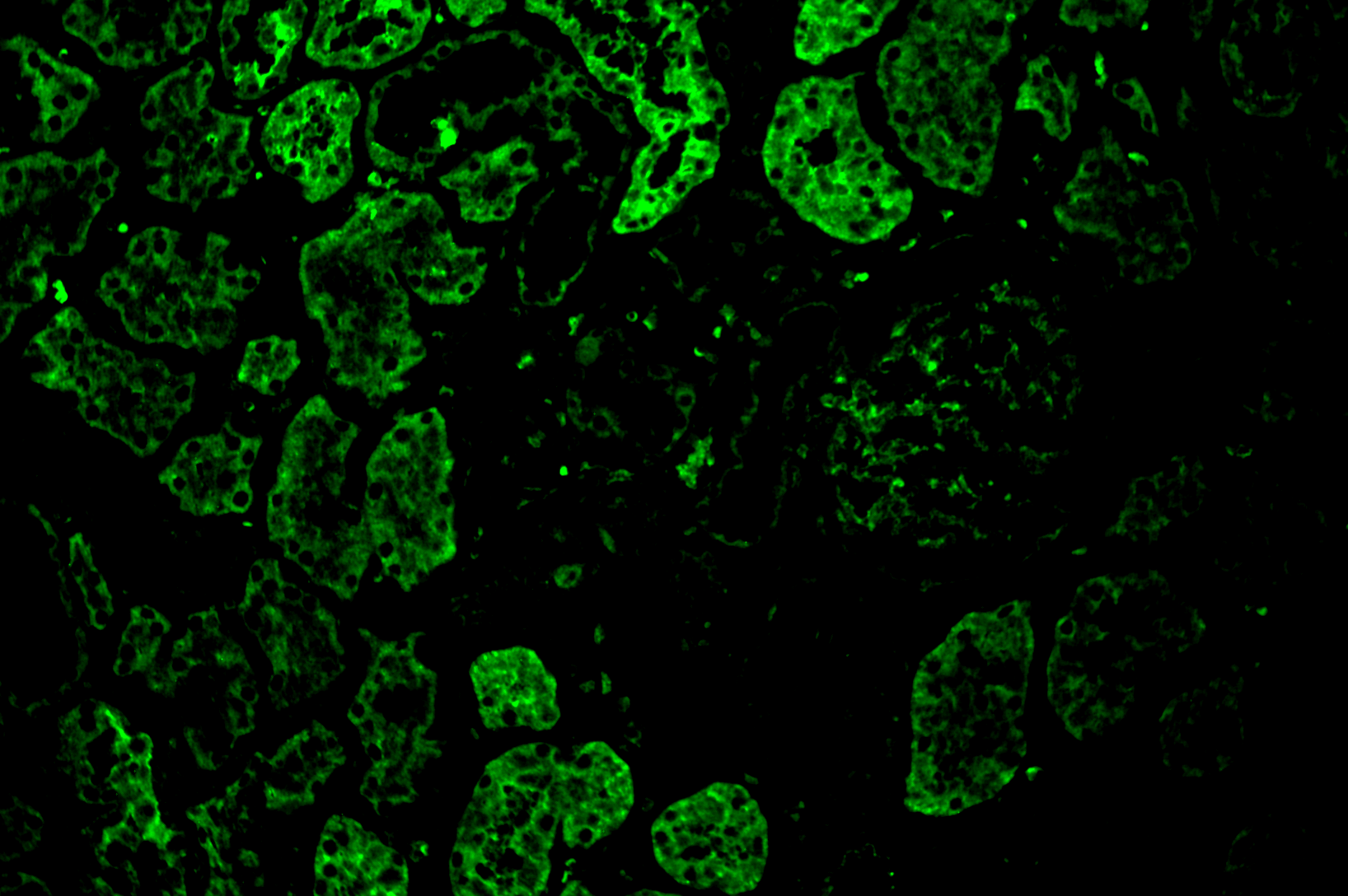

Supplement: Supplementary file 10 [file DataSheet4.ZIP › 3. IgAN patients IF,APOC1 in IgAN/Ctrl/green.tif]

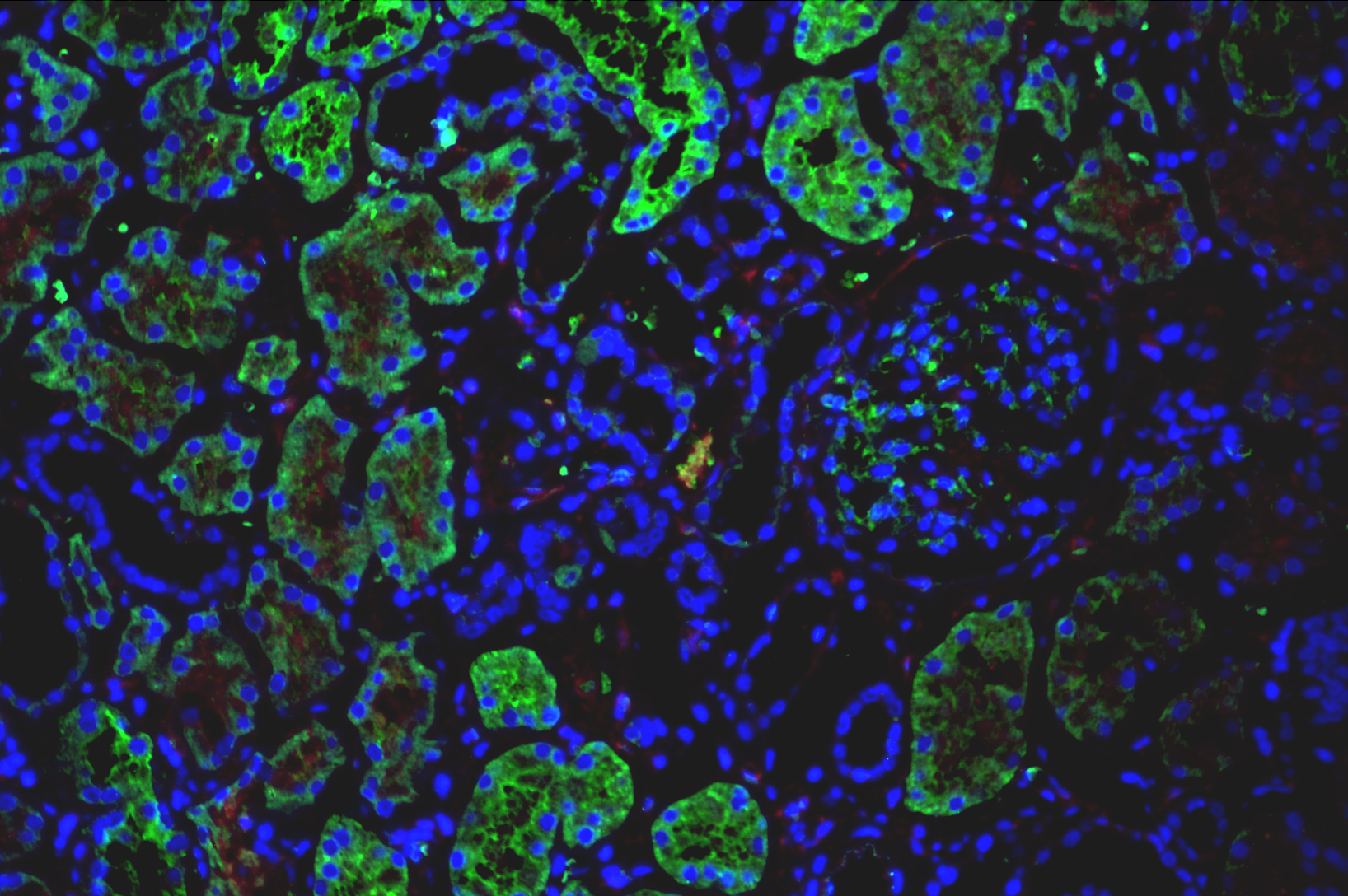

Supplement: Supplementary file 10 [file DataSheet4.ZIP › 3. IgAN patients IF,APOC1 in IgAN/Ctrl/merge.tif]

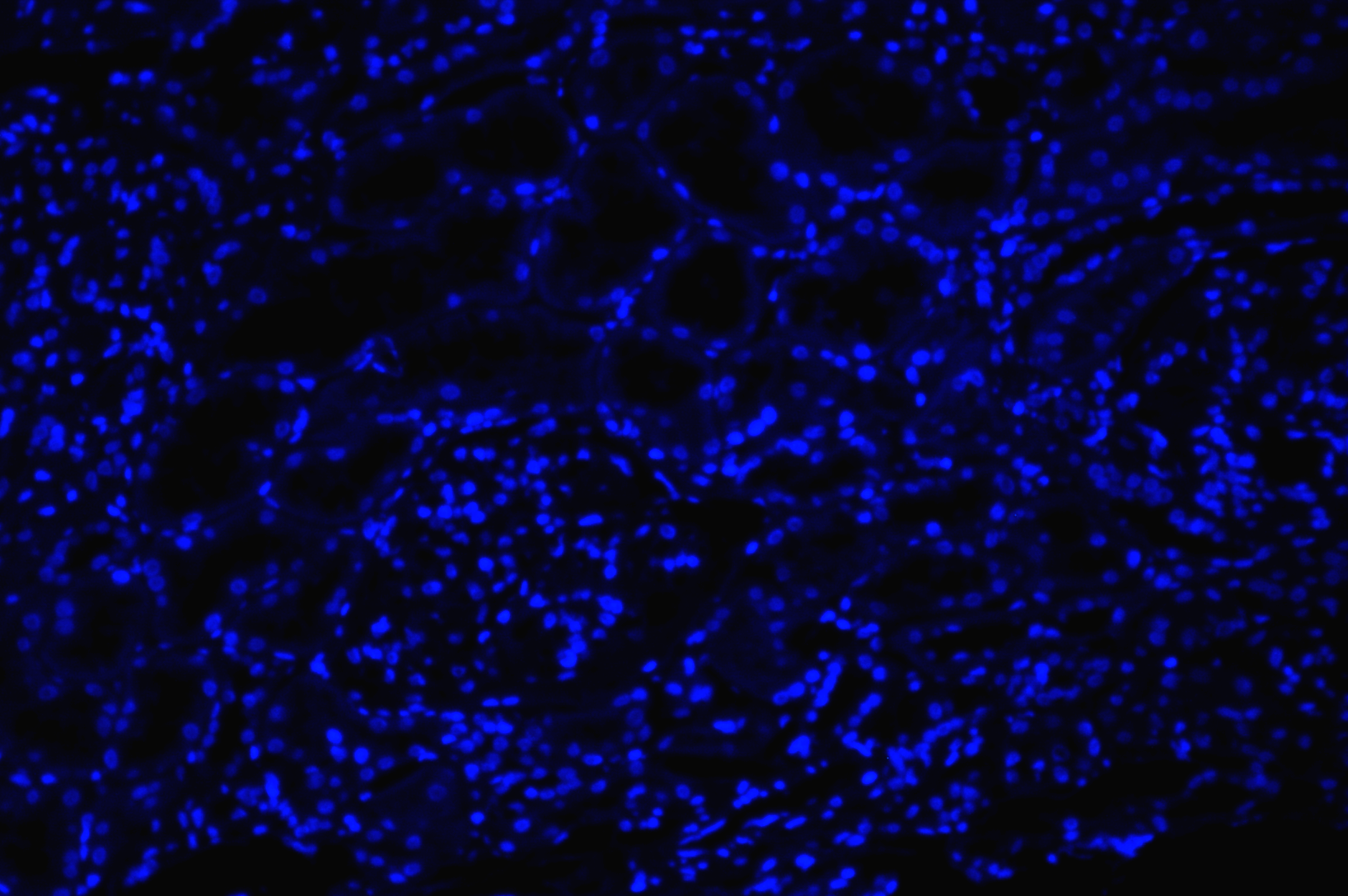

Supplement: Supplementary file 10 [file DataSheet4.ZIP › 3. IgAN patients IF,APOC1 in IgAN/IgAN/DAPI.tif]

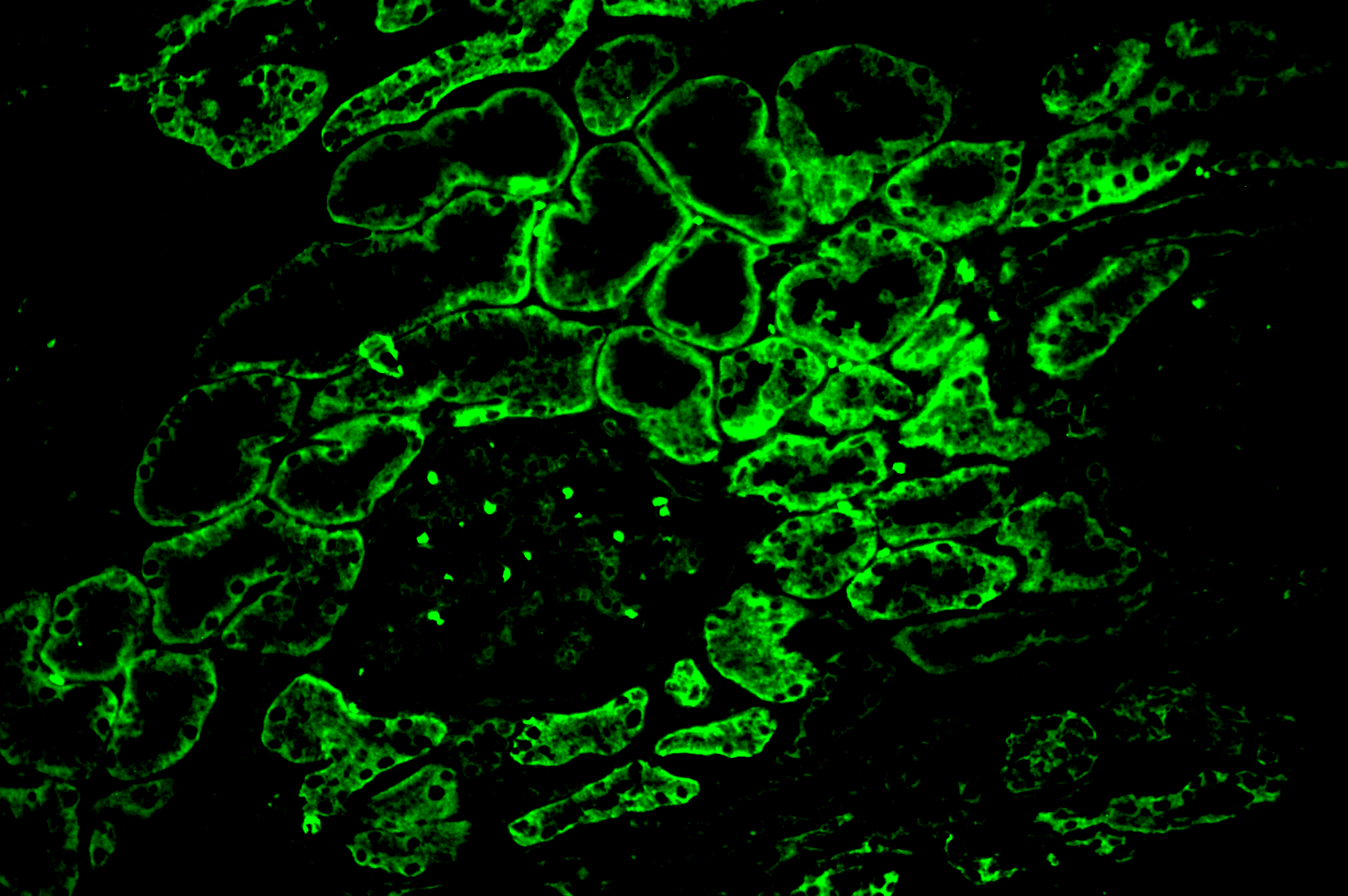

Supplement: Supplementary file 10 [file DataSheet4.ZIP › 3. IgAN patients IF,APOC1 in IgAN/IgAN/green.tif]

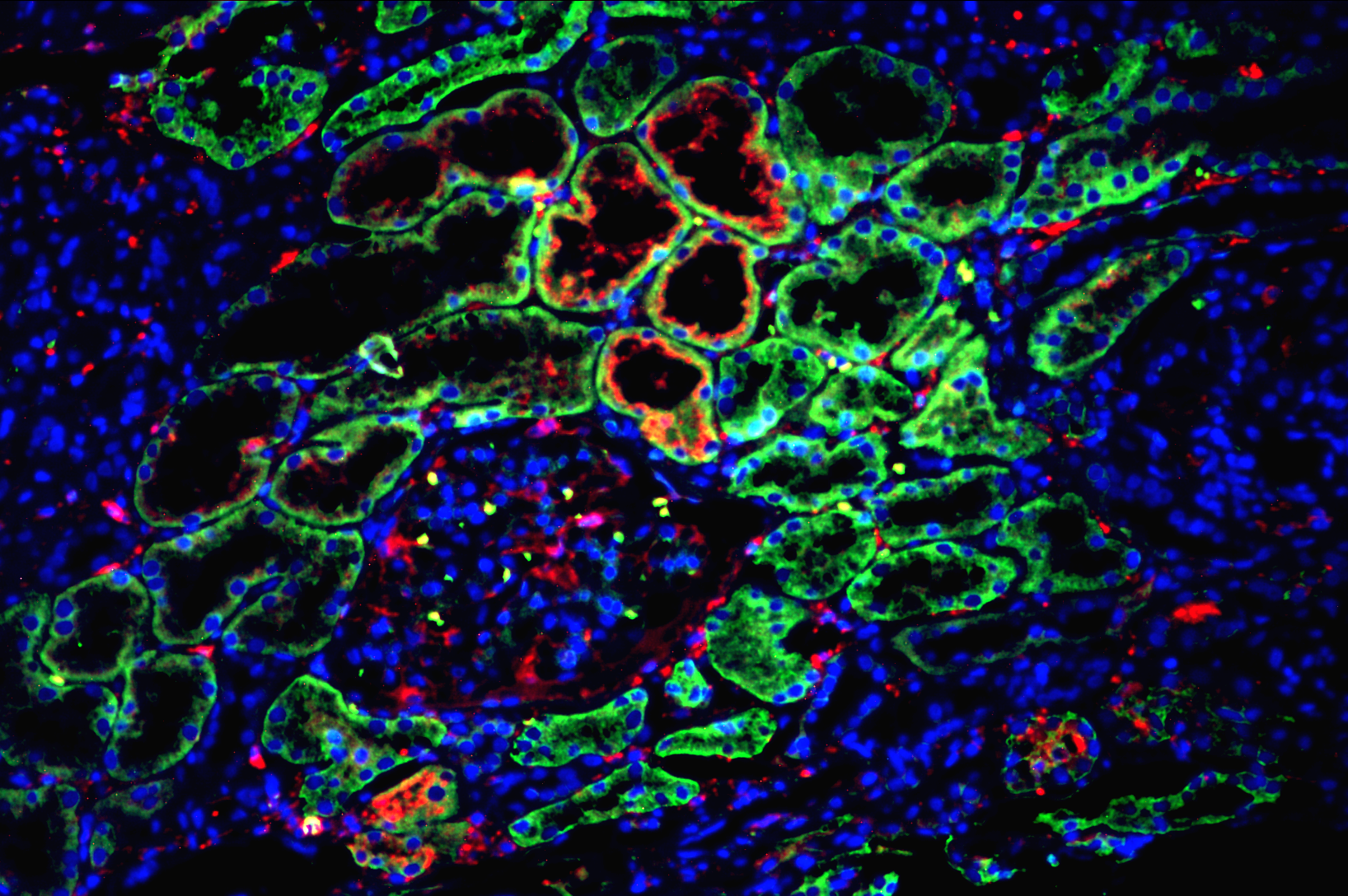

Supplement: Supplementary file 10 [file DataSheet4.ZIP › 3. IgAN patients IF,APOC1 in IgAN/IgAN/merge.tif]

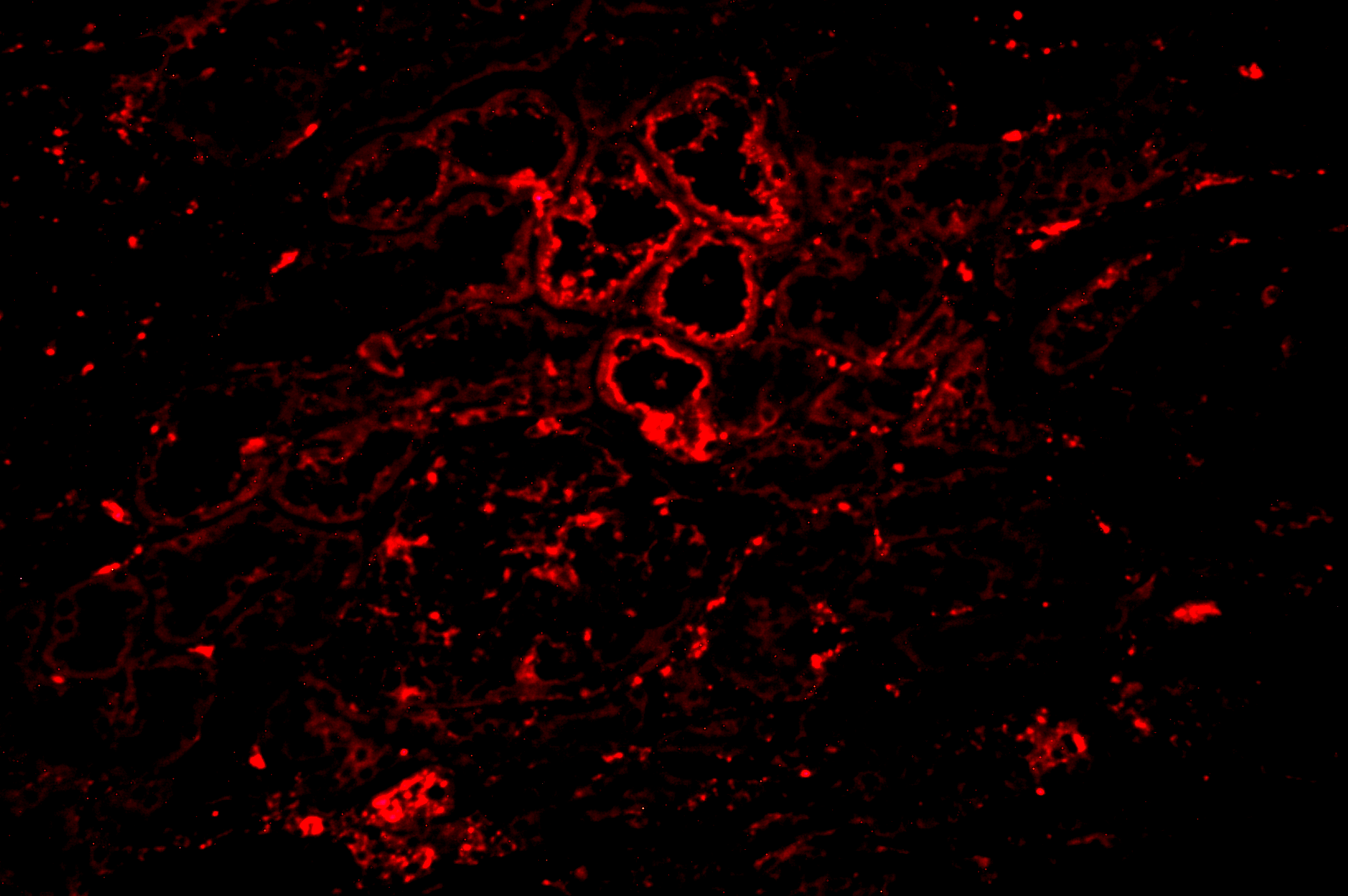

Supplement: Supplementary file 10 [file DataSheet4.ZIP › 3. IgAN patients IF,APOC1 in IgAN/IgAN/red2.tif]

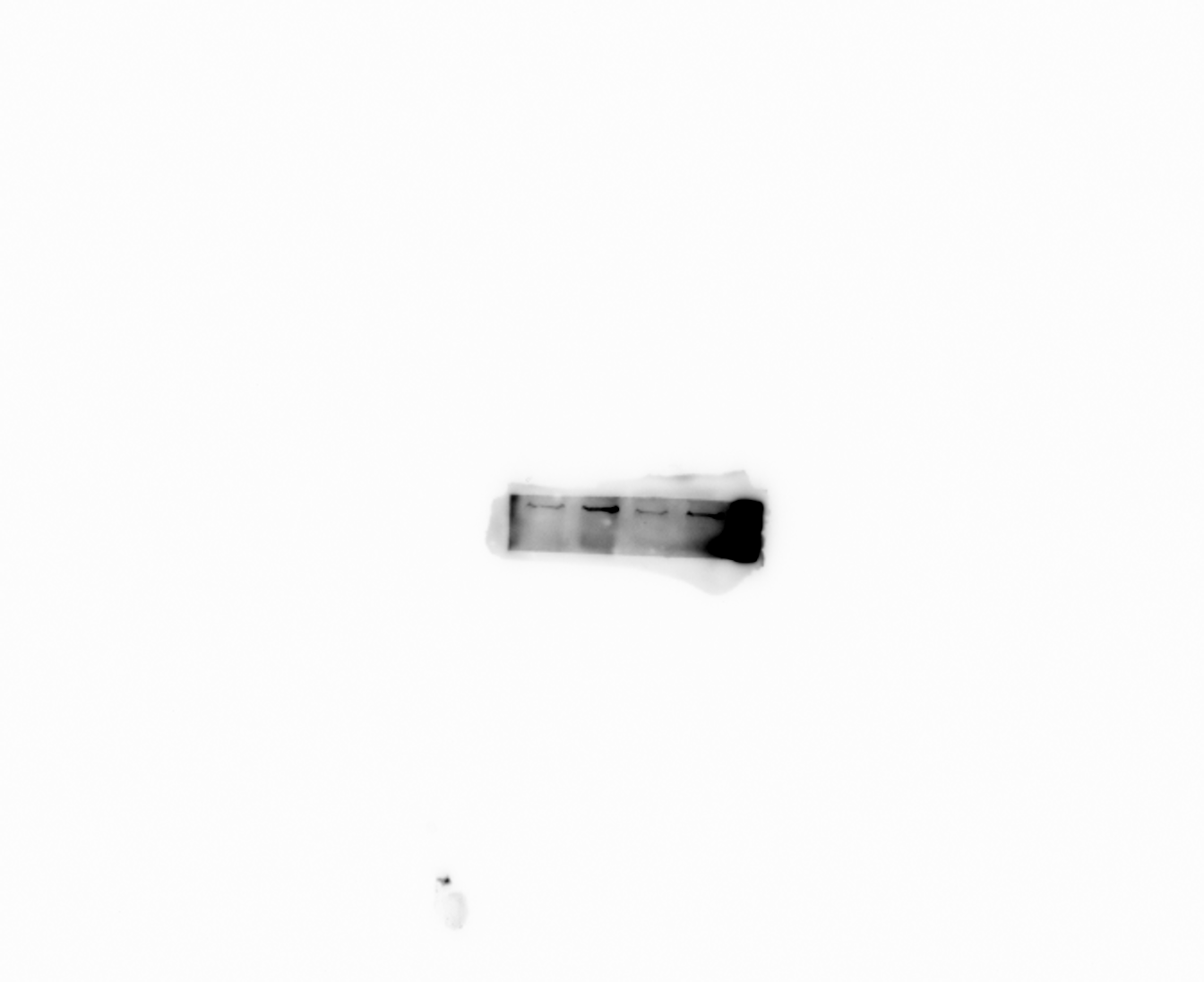

Supplement: Supplementary file 11 [file DataSheet13.ZIP › Fibrosis related protein WB(OE+EVP4593)/E-cadherin1-1.Tif]

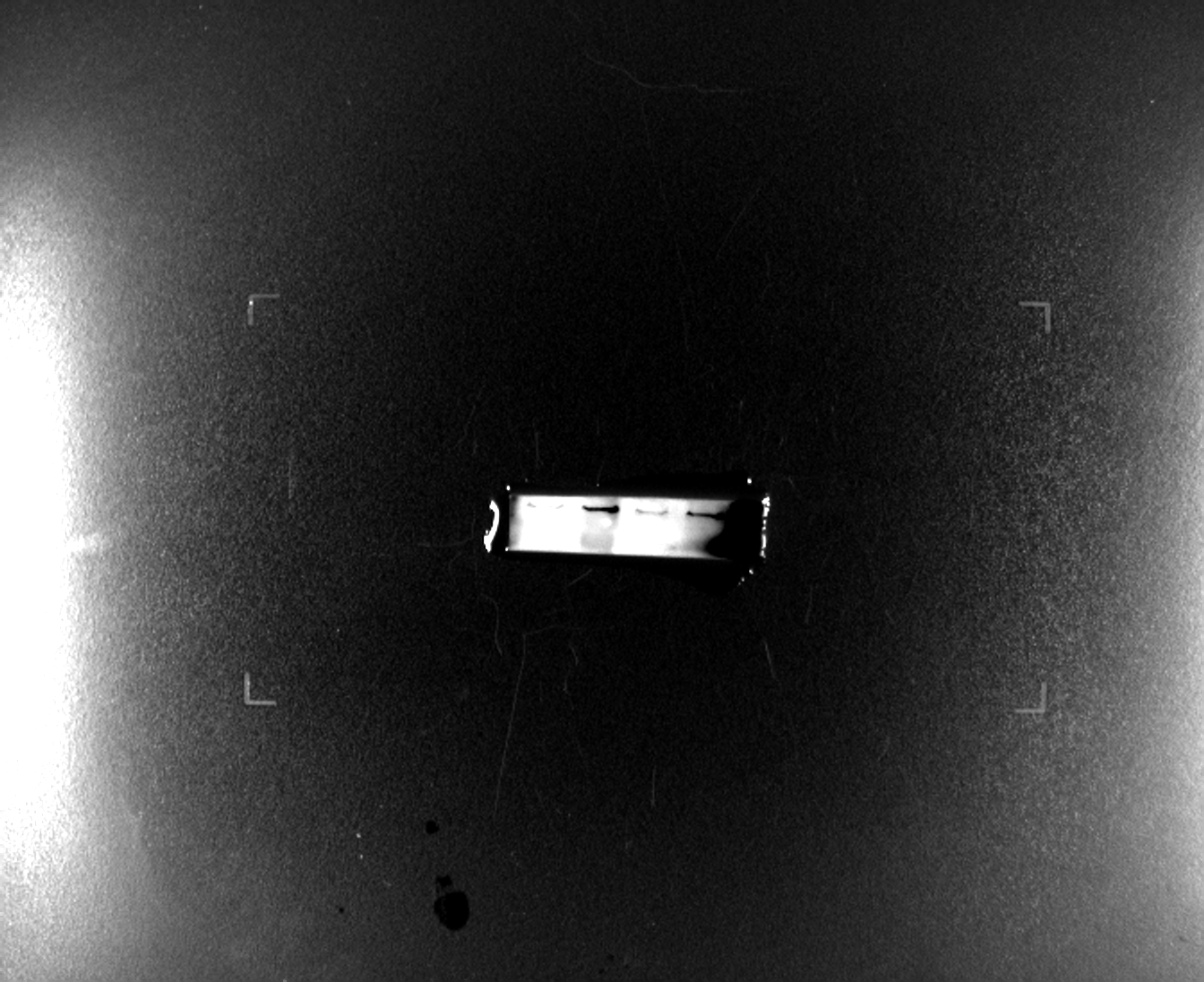

Supplement: Supplementary file 11 [file DataSheet13.ZIP › Fibrosis related protein WB(OE+EVP4593)/E-cadherin1-2.Tif]

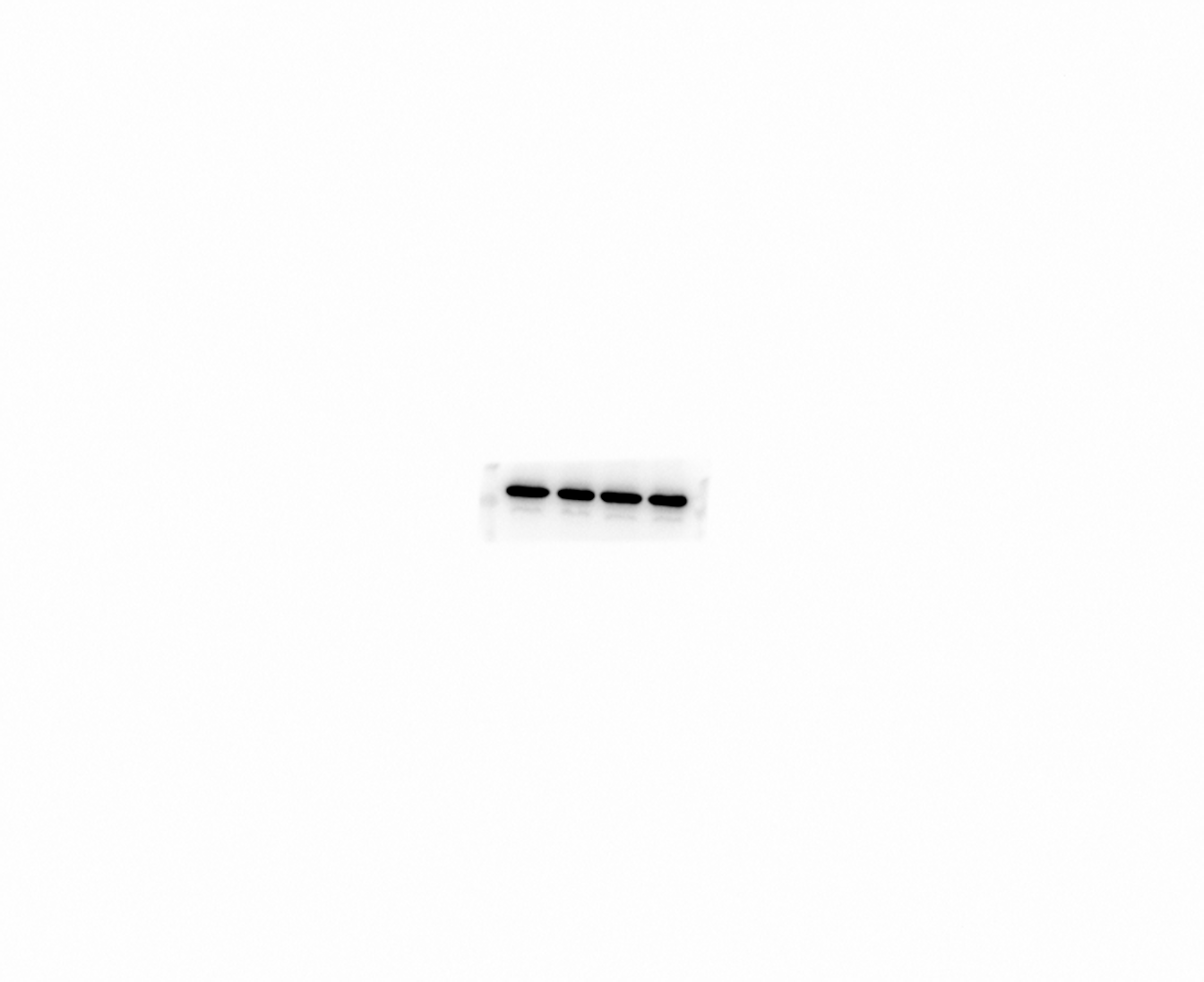

Supplement: Supplementary file 11 [file DataSheet13.ZIP › Fibrosis related protein WB(OE+EVP4593)/GAPDH1-1.Tif]

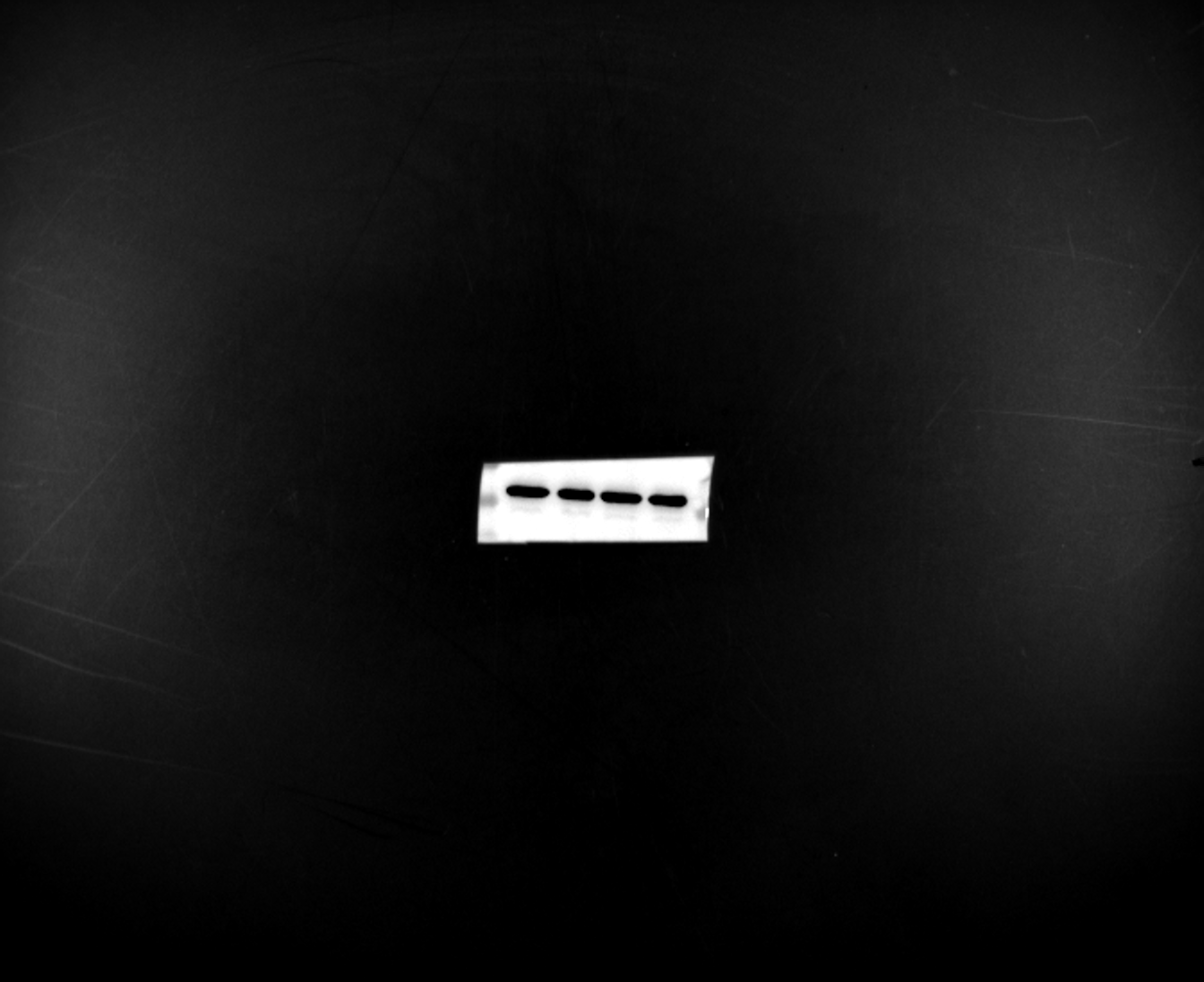

Supplement: Supplementary file 11 [file DataSheet13.ZIP › Fibrosis related protein WB(OE+EVP4593)/GAPDH1-2.Tif]

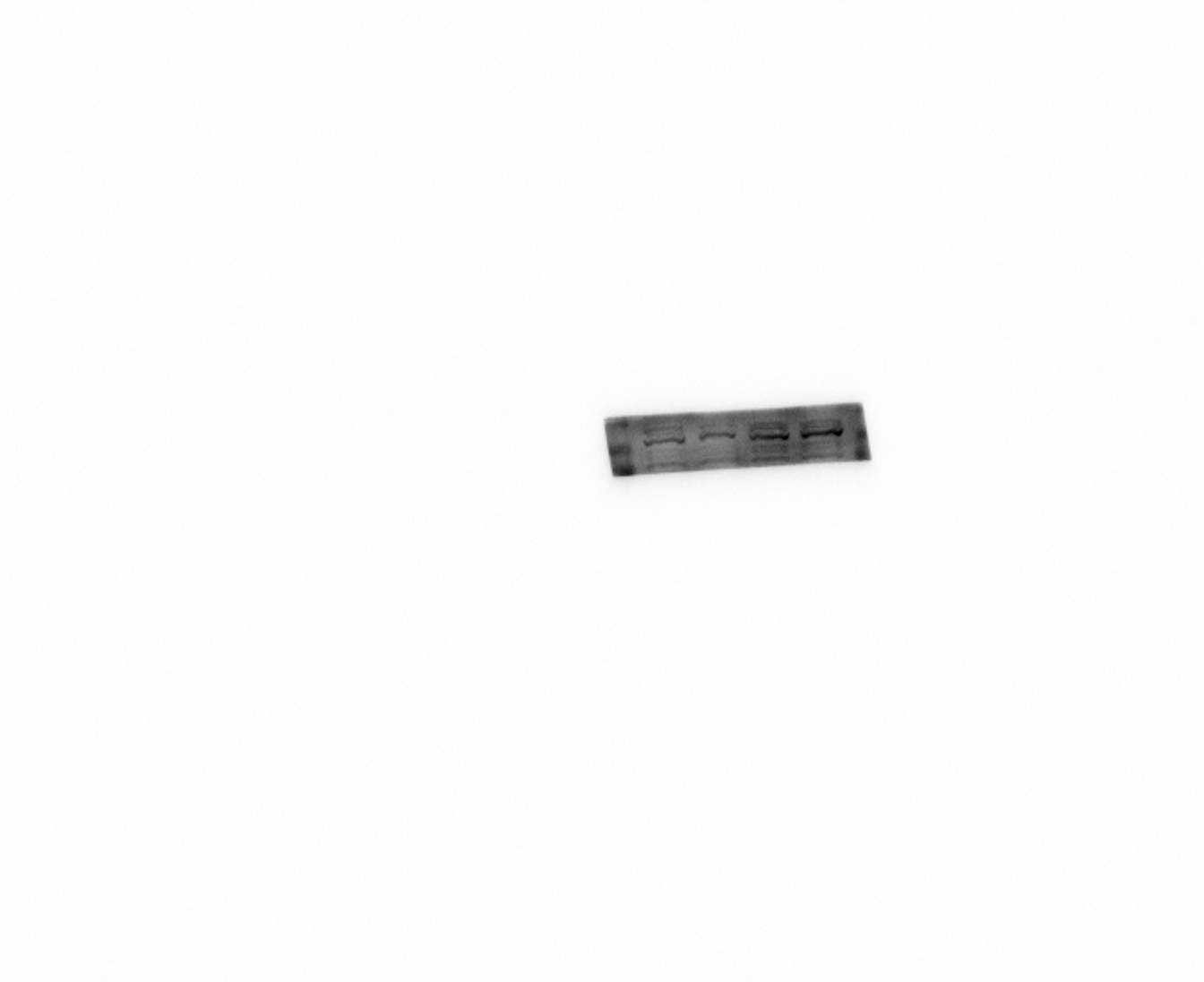

Supplement: Supplementary file 11 [file DataSheet13.ZIP › Fibrosis related protein WB(OE+EVP4593)/N-cadherin1-1.Tif]

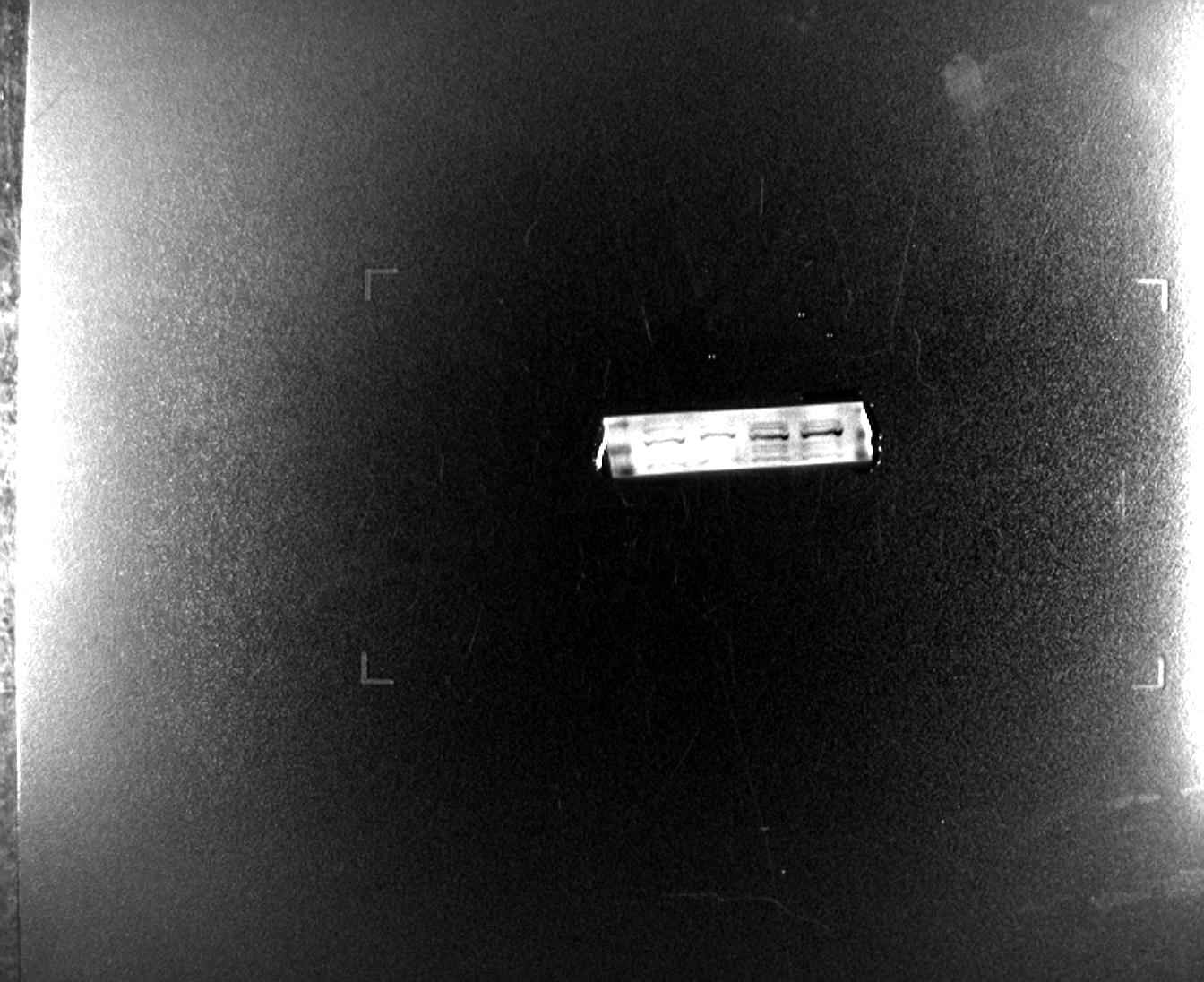

Supplement: Supplementary file 11 [file DataSheet13.ZIP › Fibrosis related protein WB(OE+EVP4593)/N-cadherin1-2.Tif]

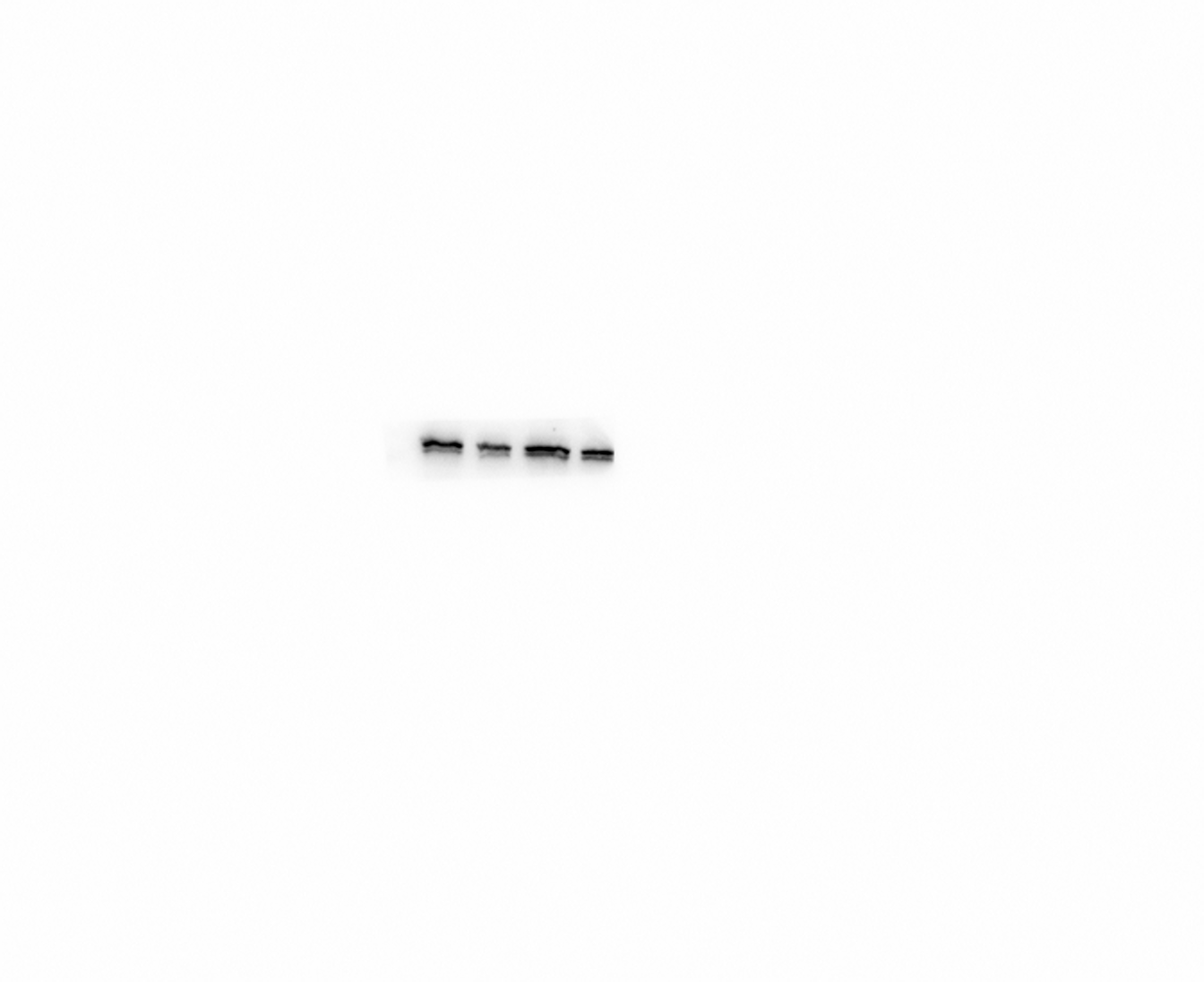

Supplement: Supplementary file 11 [file DataSheet13.ZIP › Fibrosis related protein WB(OE+EVP4593)/SMA1-1.Tif]

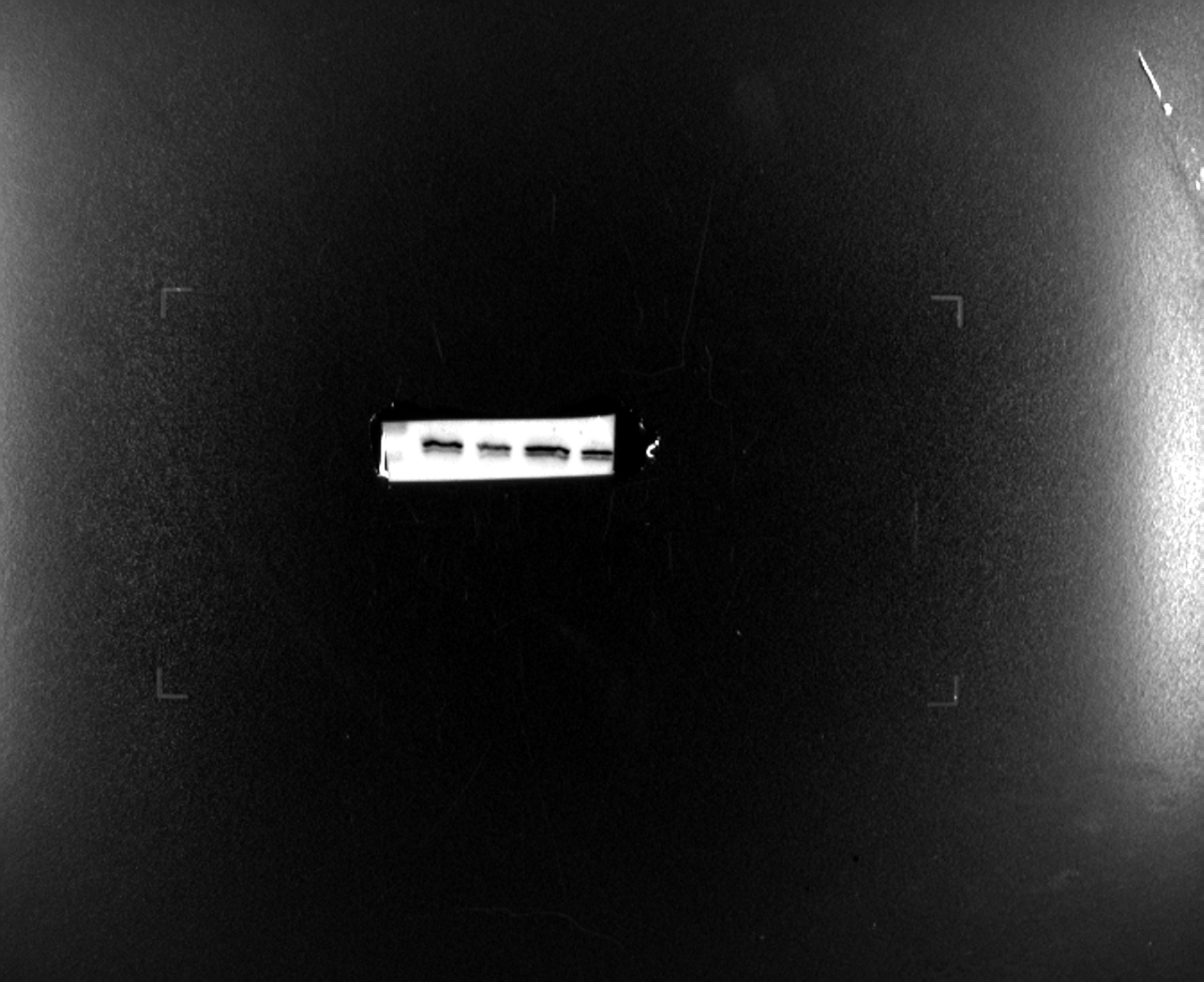

Supplement: Supplementary file 11 [file DataSheet13.ZIP › Fibrosis related protein WB(OE+EVP4593)/SMA1-2.Tif]

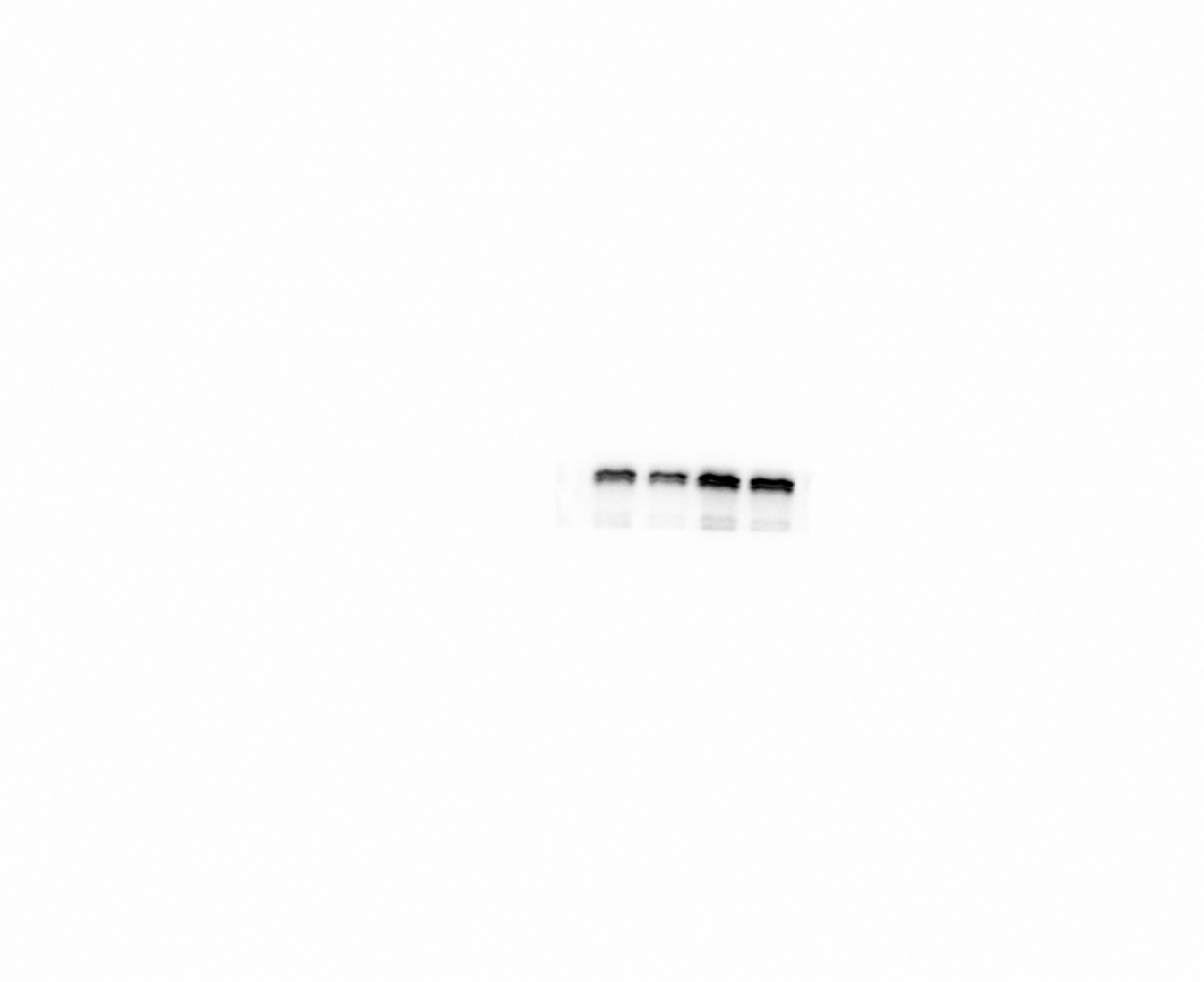

Supplement: Supplementary file 11 [file DataSheet13.ZIP › Fibrosis related protein WB(OE+EVP4593)/Vimentin1-1.Tif]

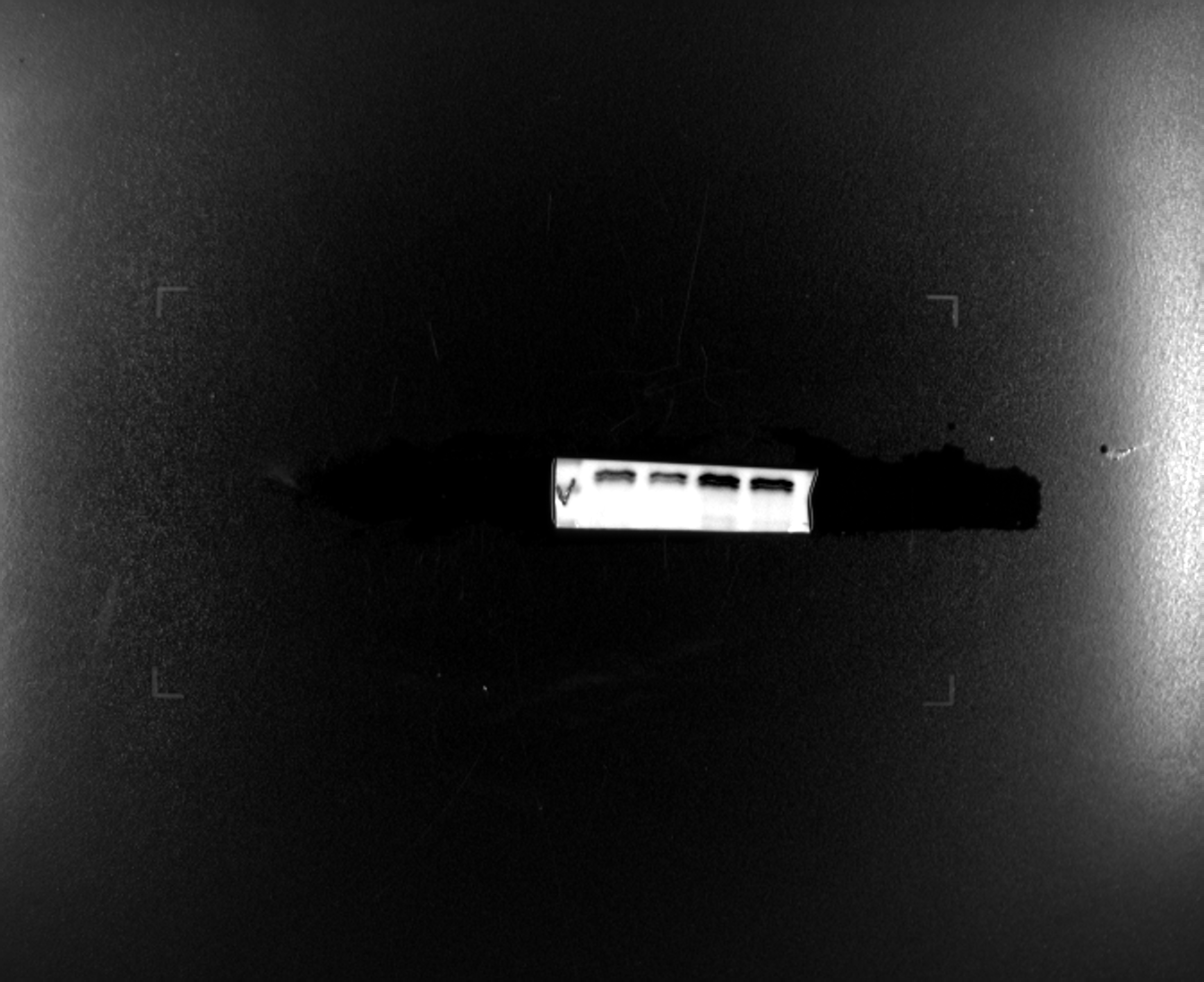

Supplement: Supplementary file 11 [file DataSheet13.ZIP › Fibrosis related protein WB(OE+EVP4593)/Vimentin1-2.Tif]

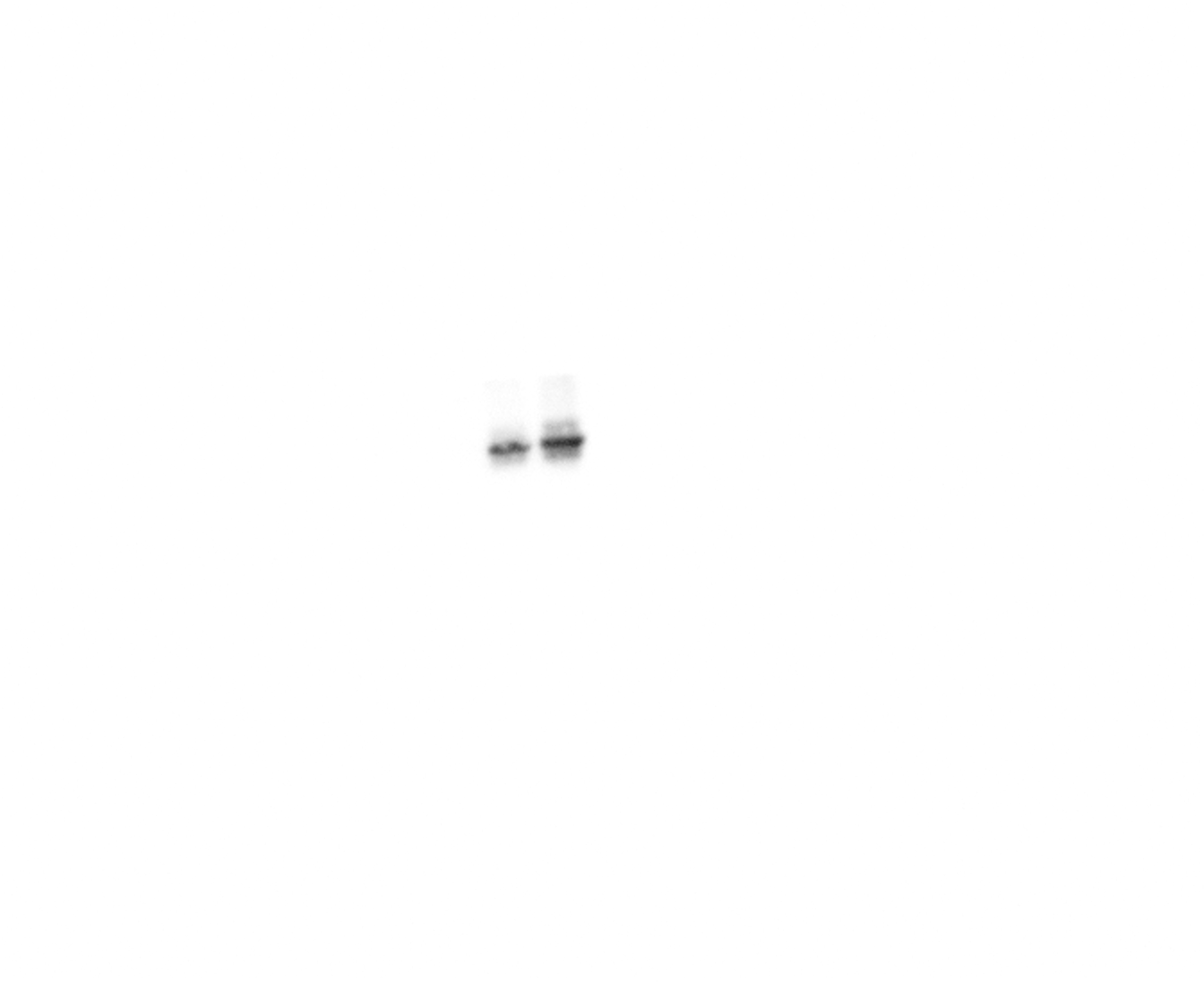

Supplement: Supplementary file 16 [file DataSheet10.ZIP › 9.WB OE-APOC1 NF-a╩B/APOC122-1.Tif]

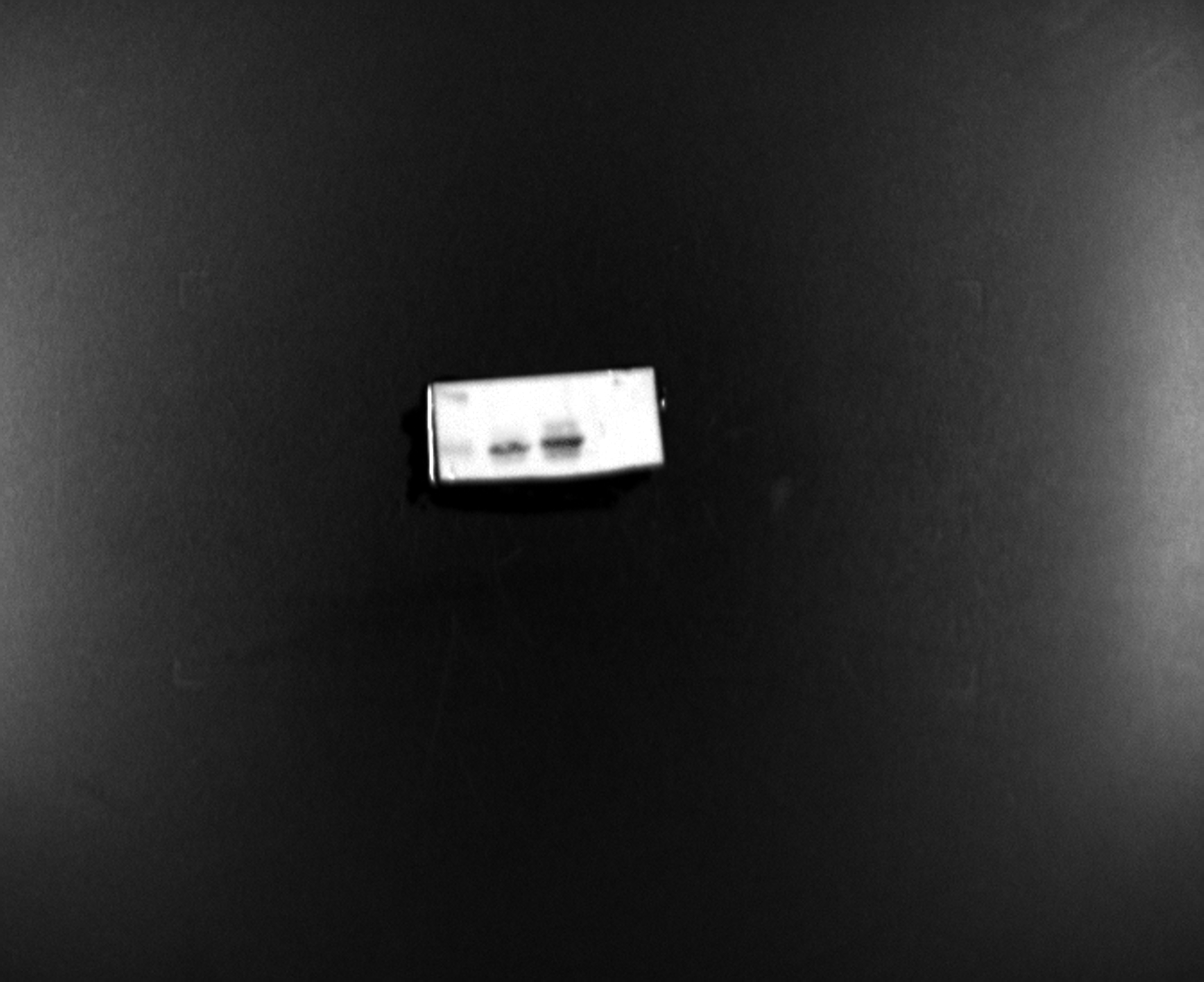

Supplement: Supplementary file 16 [file DataSheet10.ZIP › 9.WB OE-APOC1 NF-a╩B/APOC122-2.Tif]

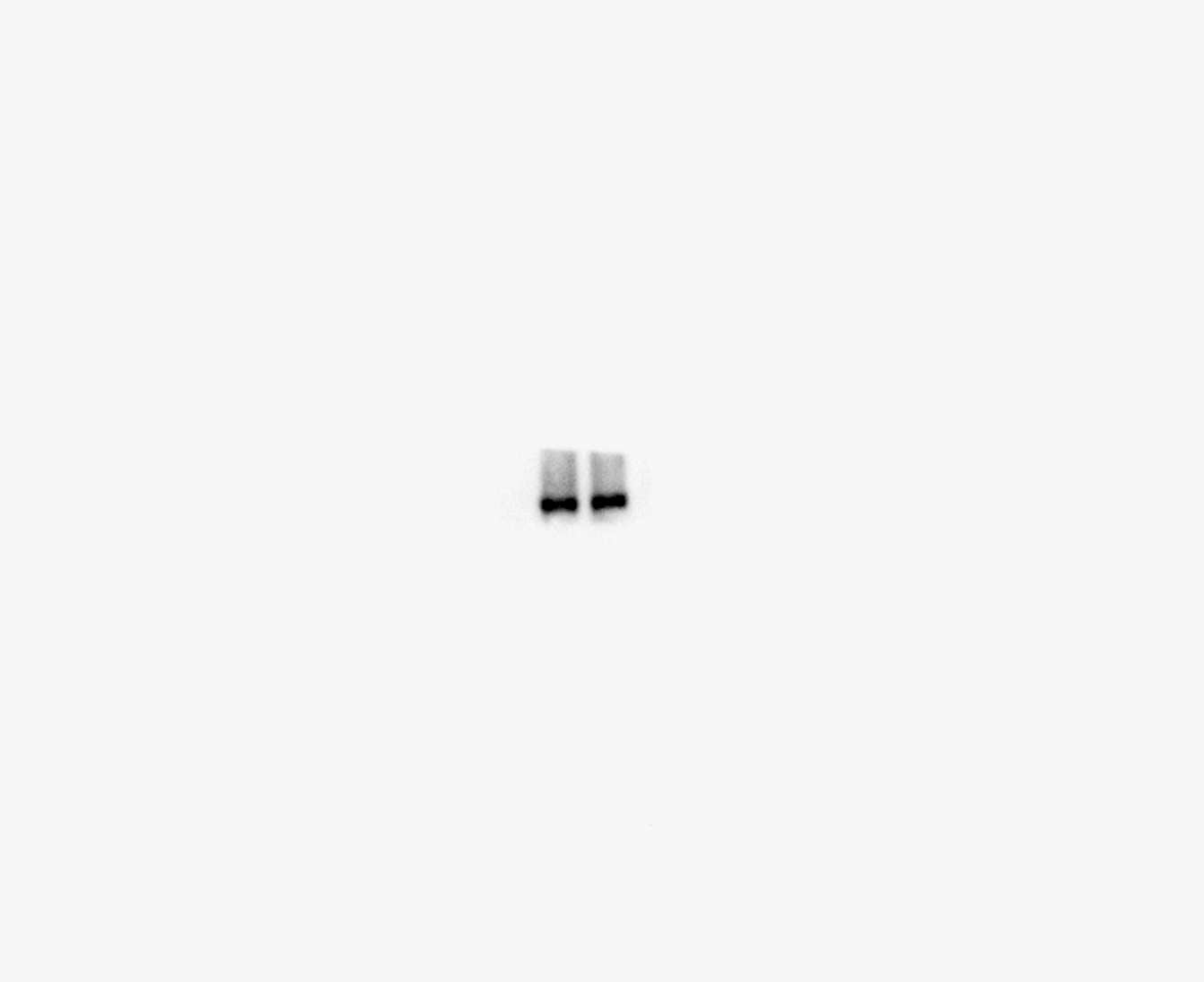

Supplement: Supplementary file 16 [file DataSheet10.ZIP › 9.WB OE-APOC1 NF-a╩B/GAPDH 39-1.Tif]

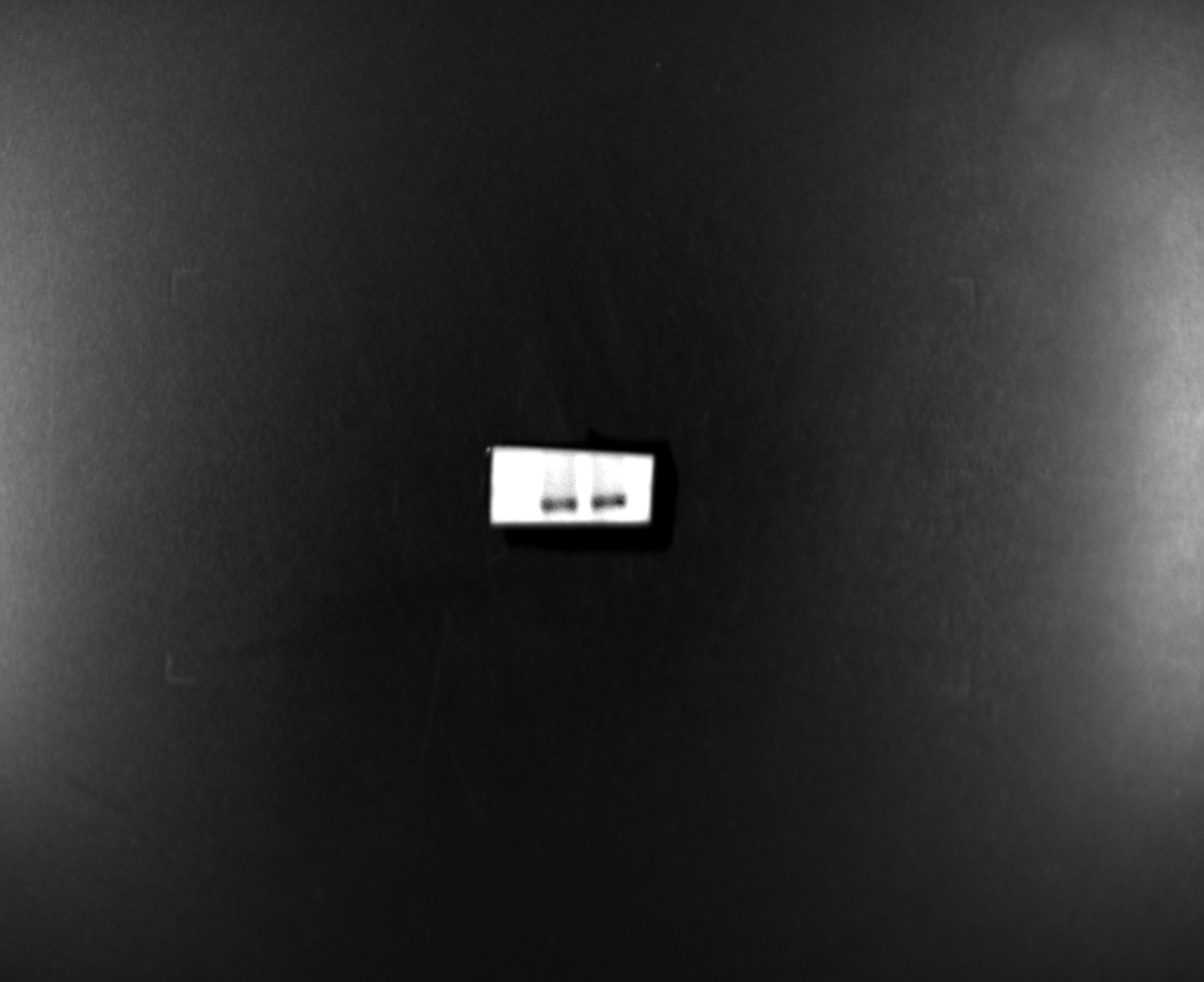

Supplement: Supplementary file 16 [file DataSheet10.ZIP › 9.WB OE-APOC1 NF-a╩B/GAPDH 39-2.Tif]

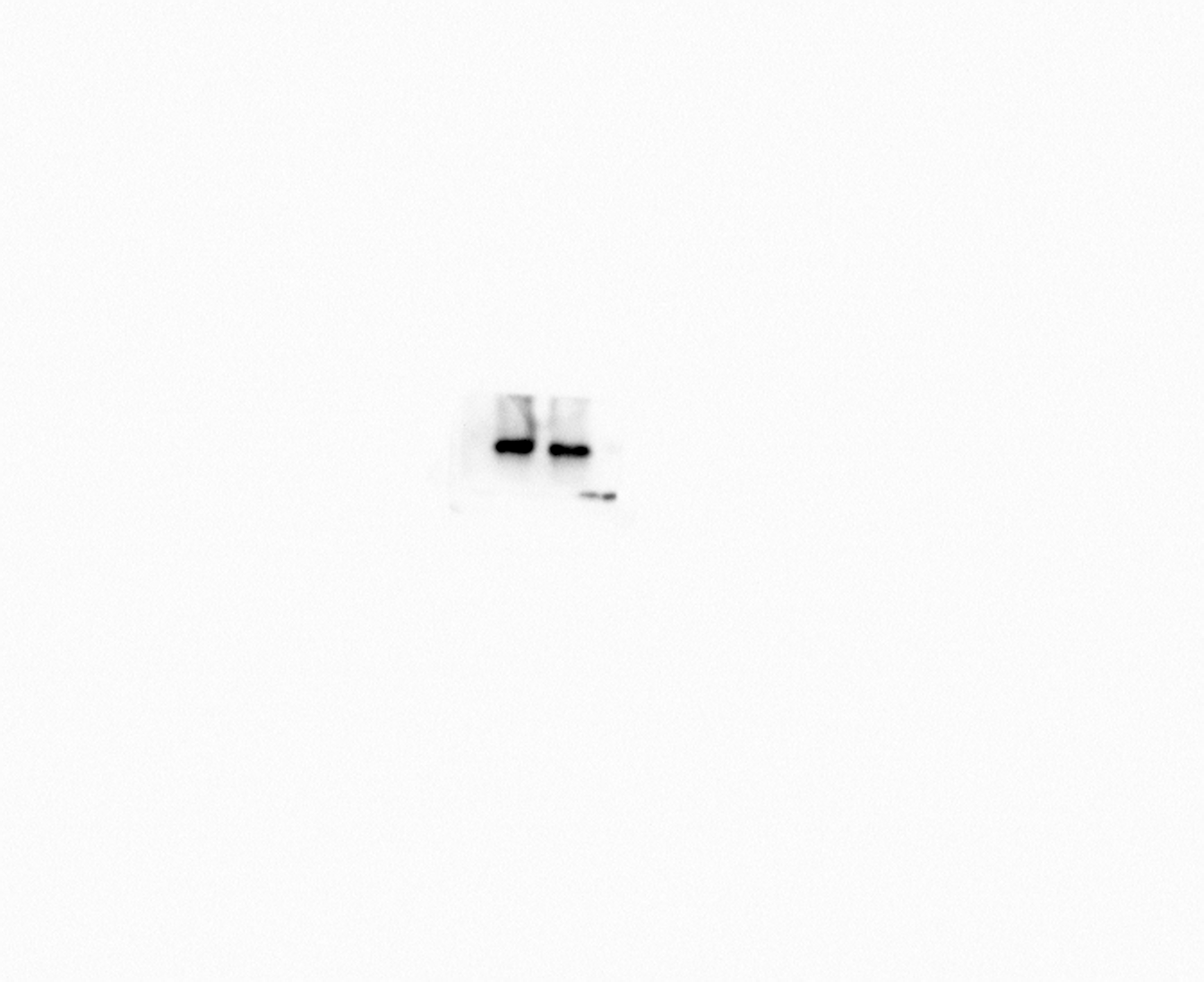

Supplement: Supplementary file 16 [file DataSheet10.ZIP › 9.WB OE-APOC1 NF-a╩B/NF-KB11-1.Tif]

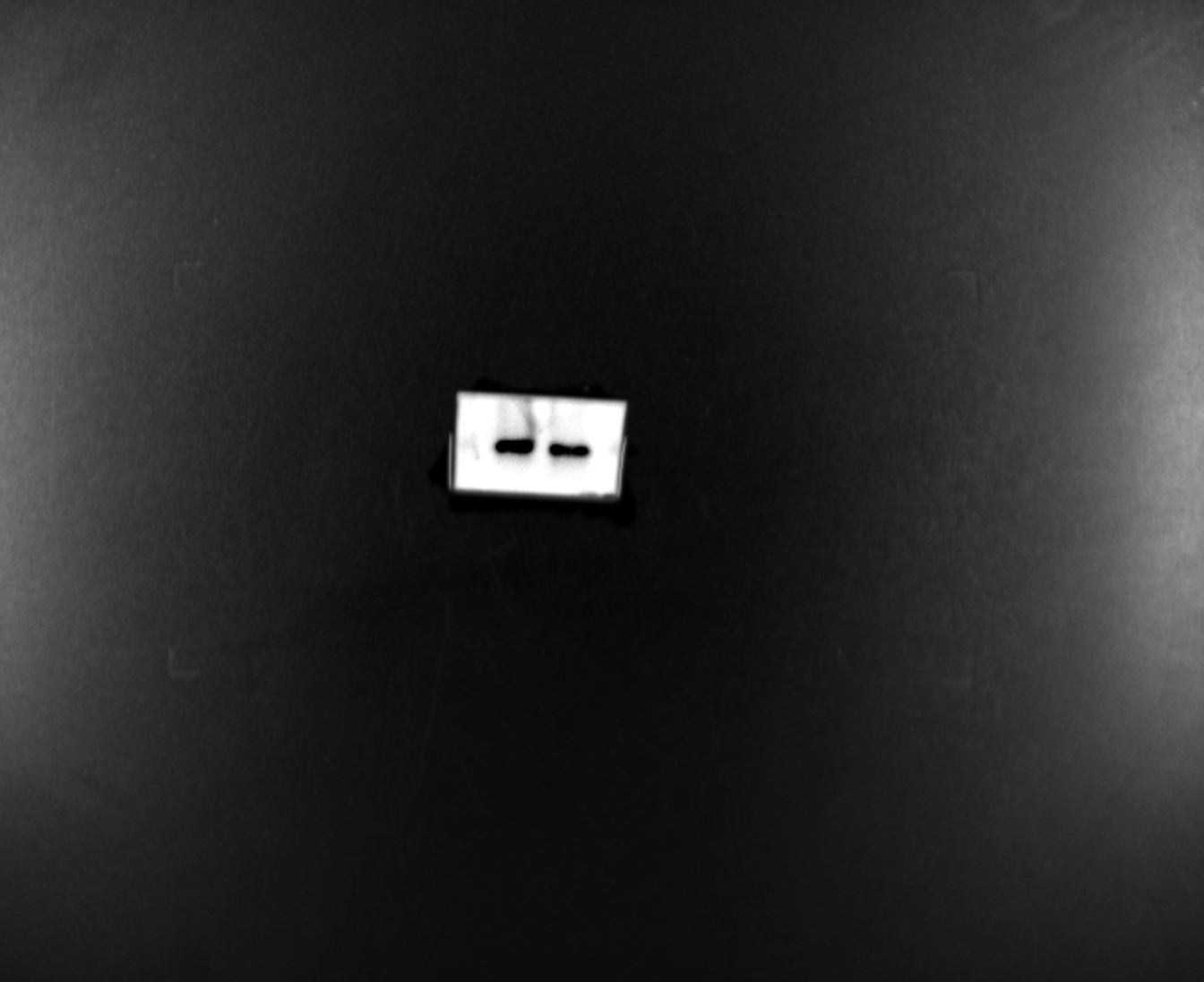

Supplement: Supplementary file 16 [file DataSheet10.ZIP › 9.WB OE-APOC1 NF-a╩B/NF-KB11-2.Tif]

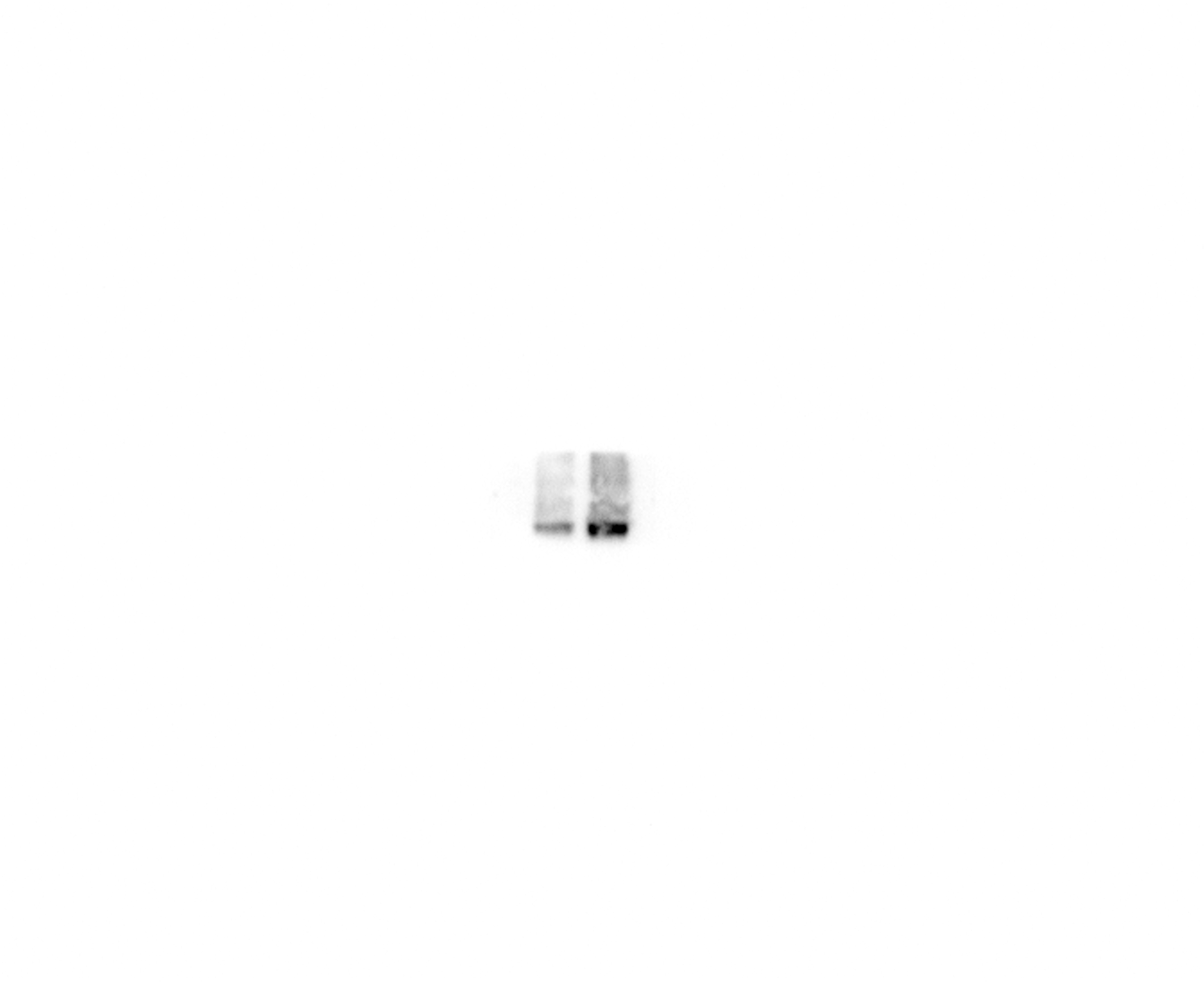

Supplement: Supplementary file 16 [file DataSheet10.ZIP › 9.WB OE-APOC1 NF-a╩B/P-NF-KB42-1.Tif]

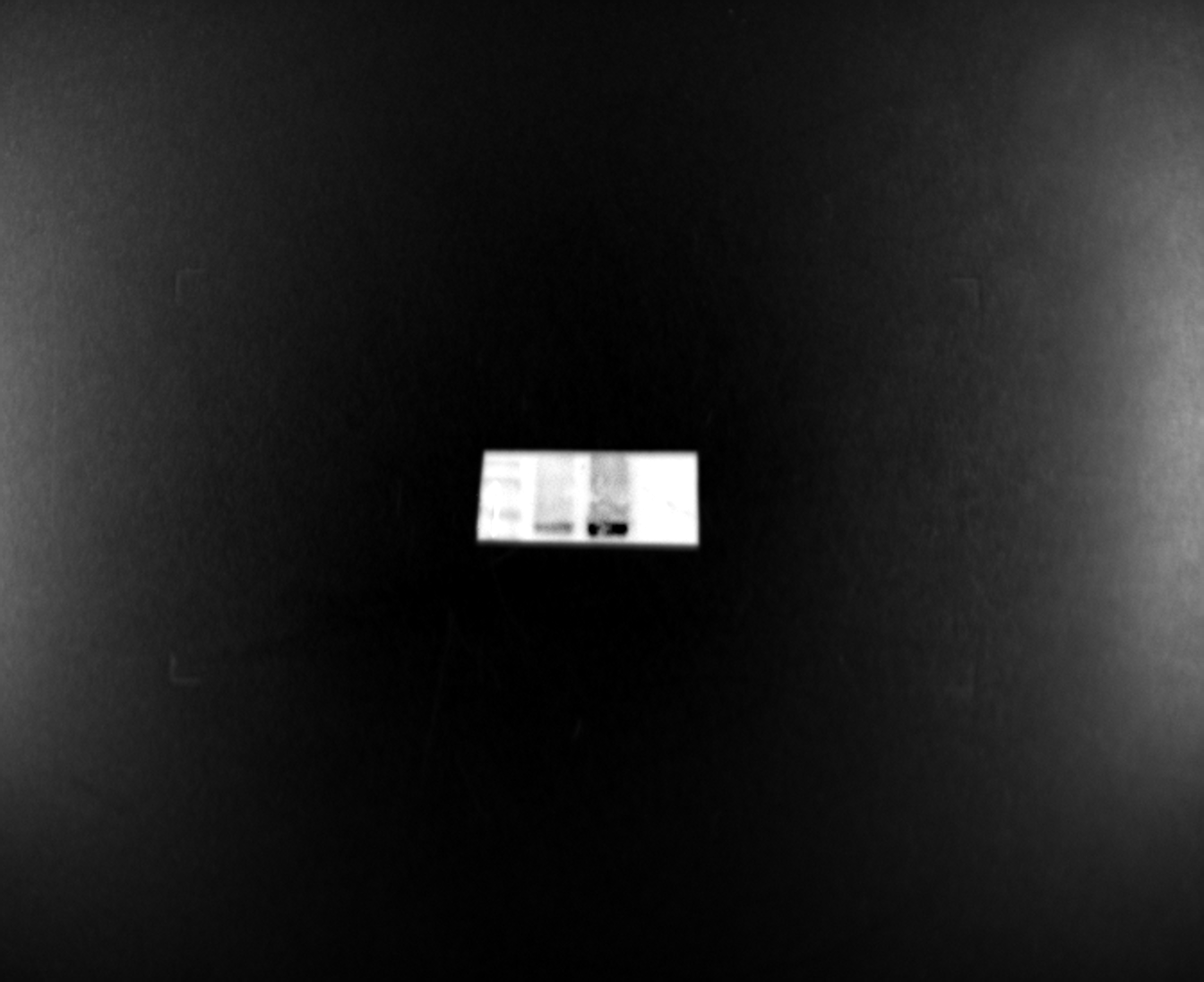

Supplement: Supplementary file 16 [file DataSheet10.ZIP › 9.WB OE-APOC1 NF-a╩B/P-NF-KB42-2.Tif]

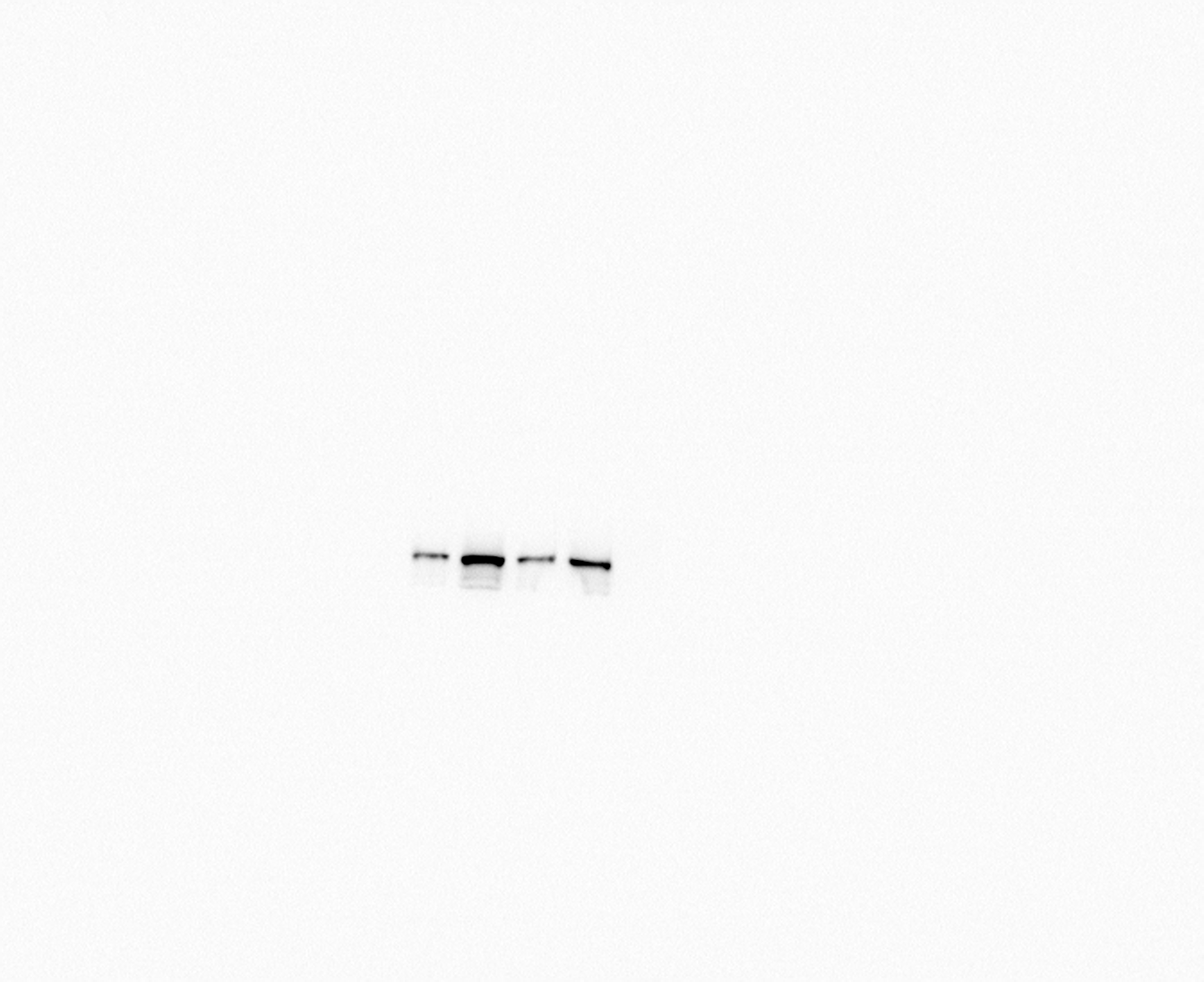

Supplement: Supplementary file 17 [file DataSheet6.ZIP › 5. WB-Fibrotic protein/APOC1 1-1.Tif]

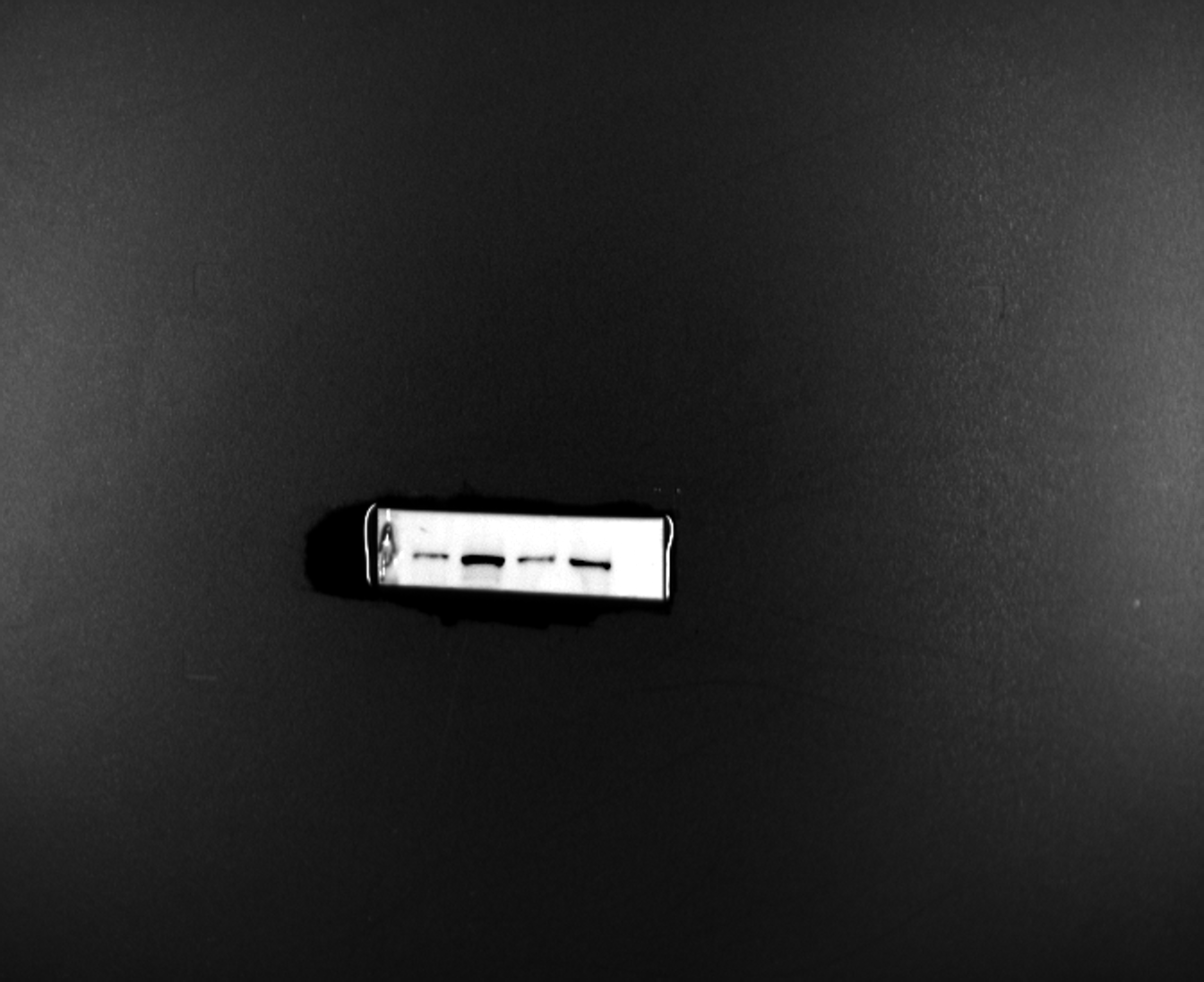

Supplement: Supplementary file 17 [file DataSheet6.ZIP › 5. WB-Fibrotic protein/APOC1 1-2.Tif]

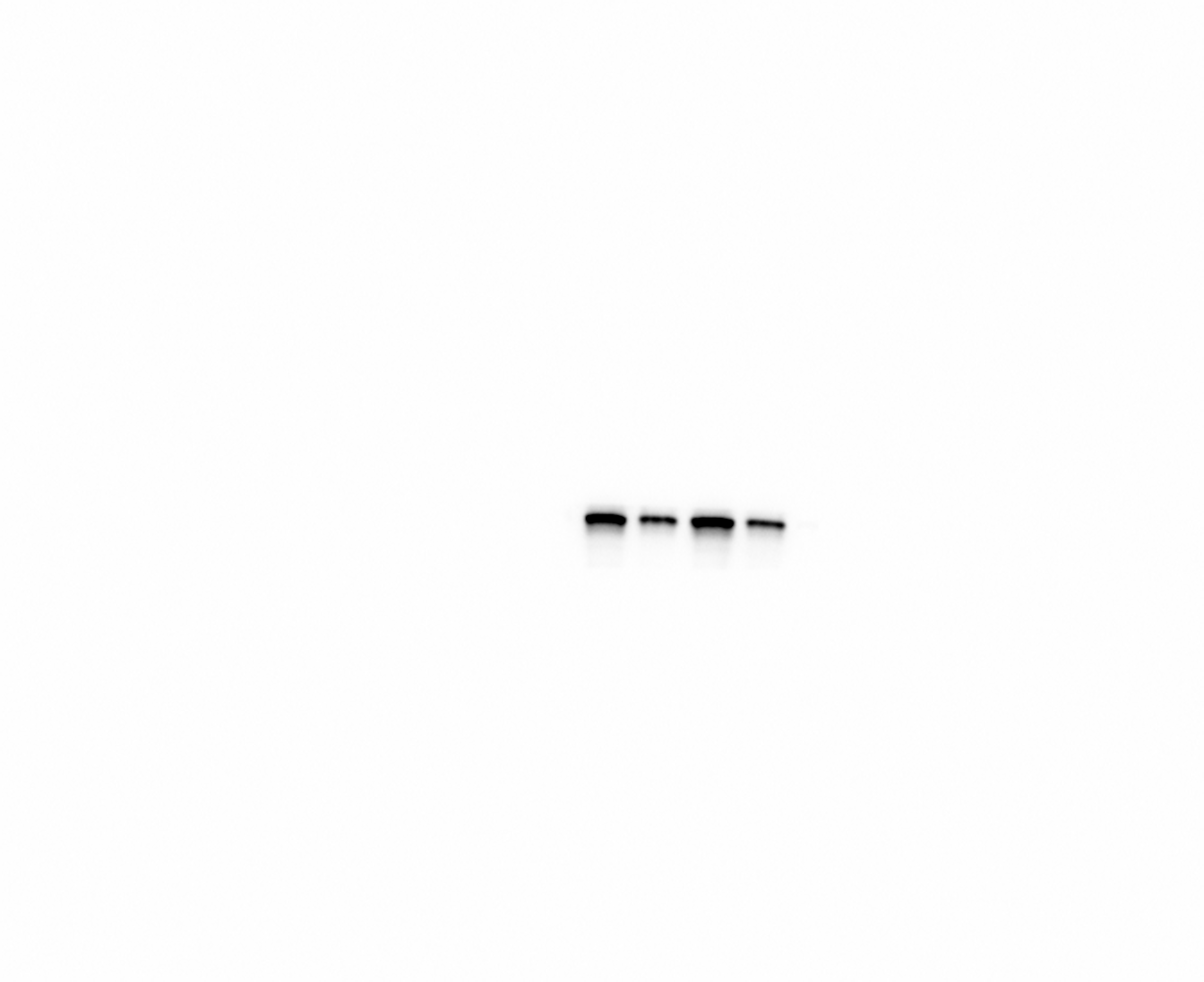

Supplement: Supplementary file 17 [file DataSheet6.ZIP › 5. WB-Fibrotic protein/E-cadherin 1-1.Tif]

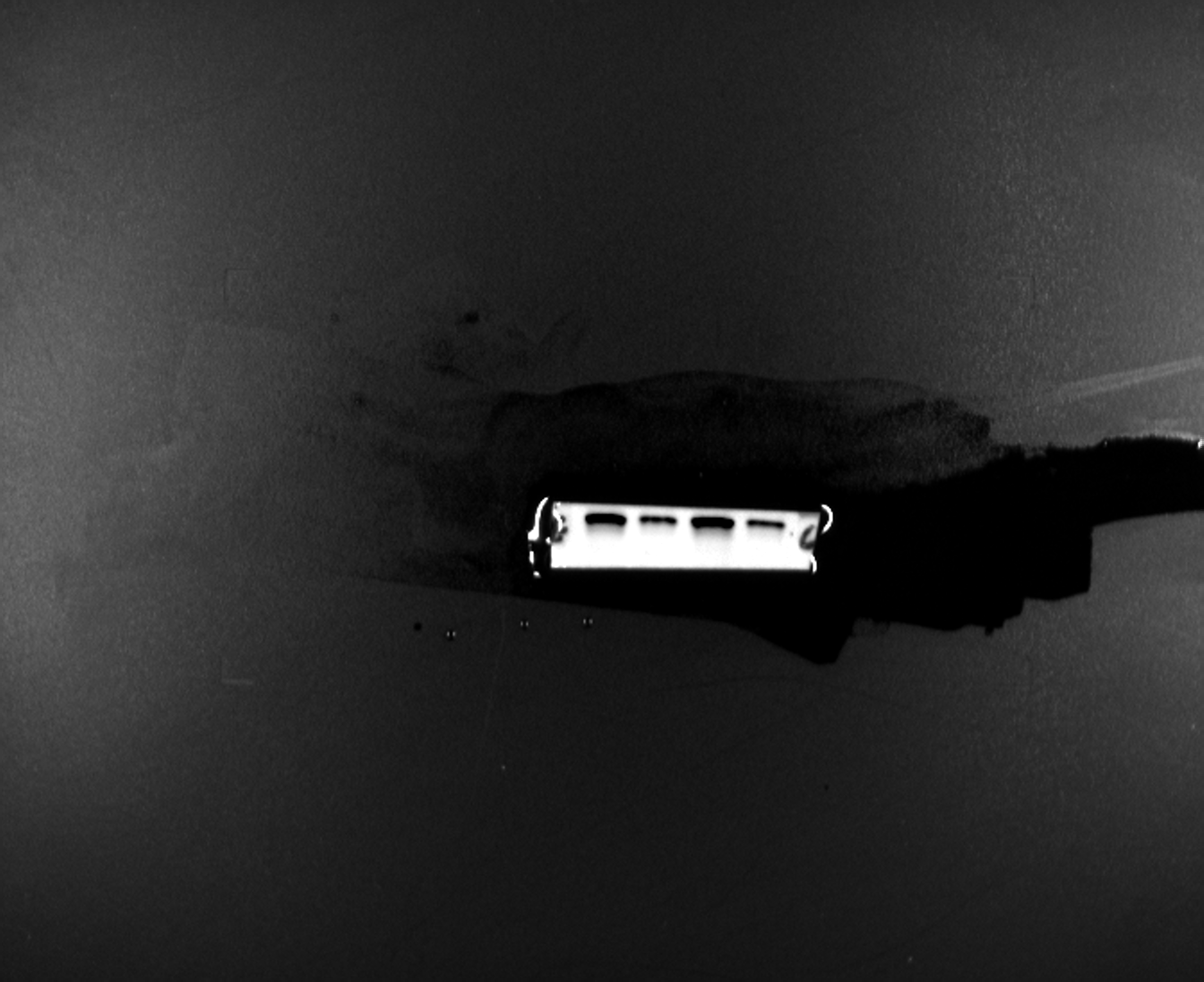

Supplement: Supplementary file 17 [file DataSheet6.ZIP › 5. WB-Fibrotic protein/E-cadherin 1-2.Tif]

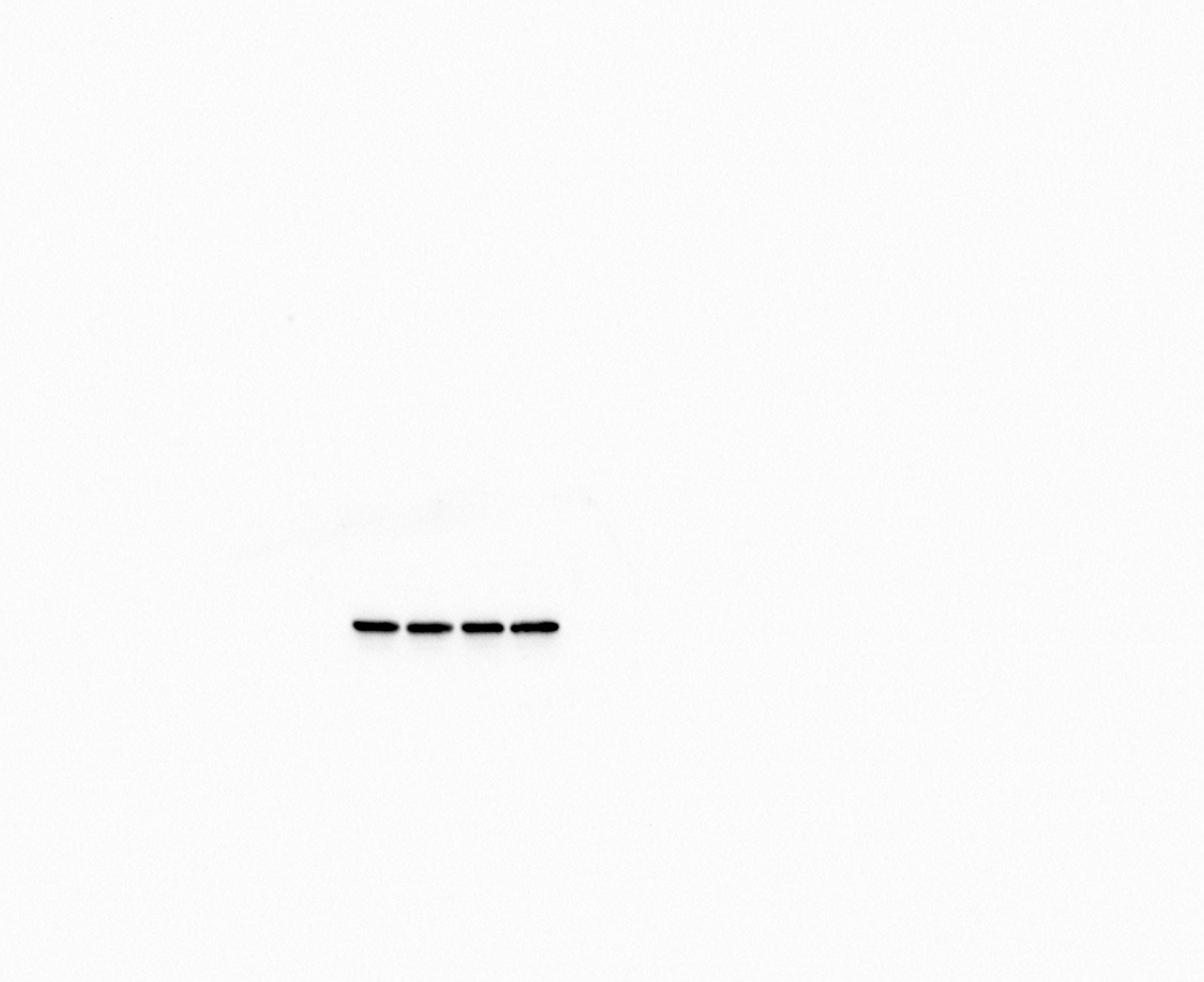

Supplement: Supplementary file 17 [file DataSheet6.ZIP › 5. WB-Fibrotic protein/GAPDH 2-1.Tif]

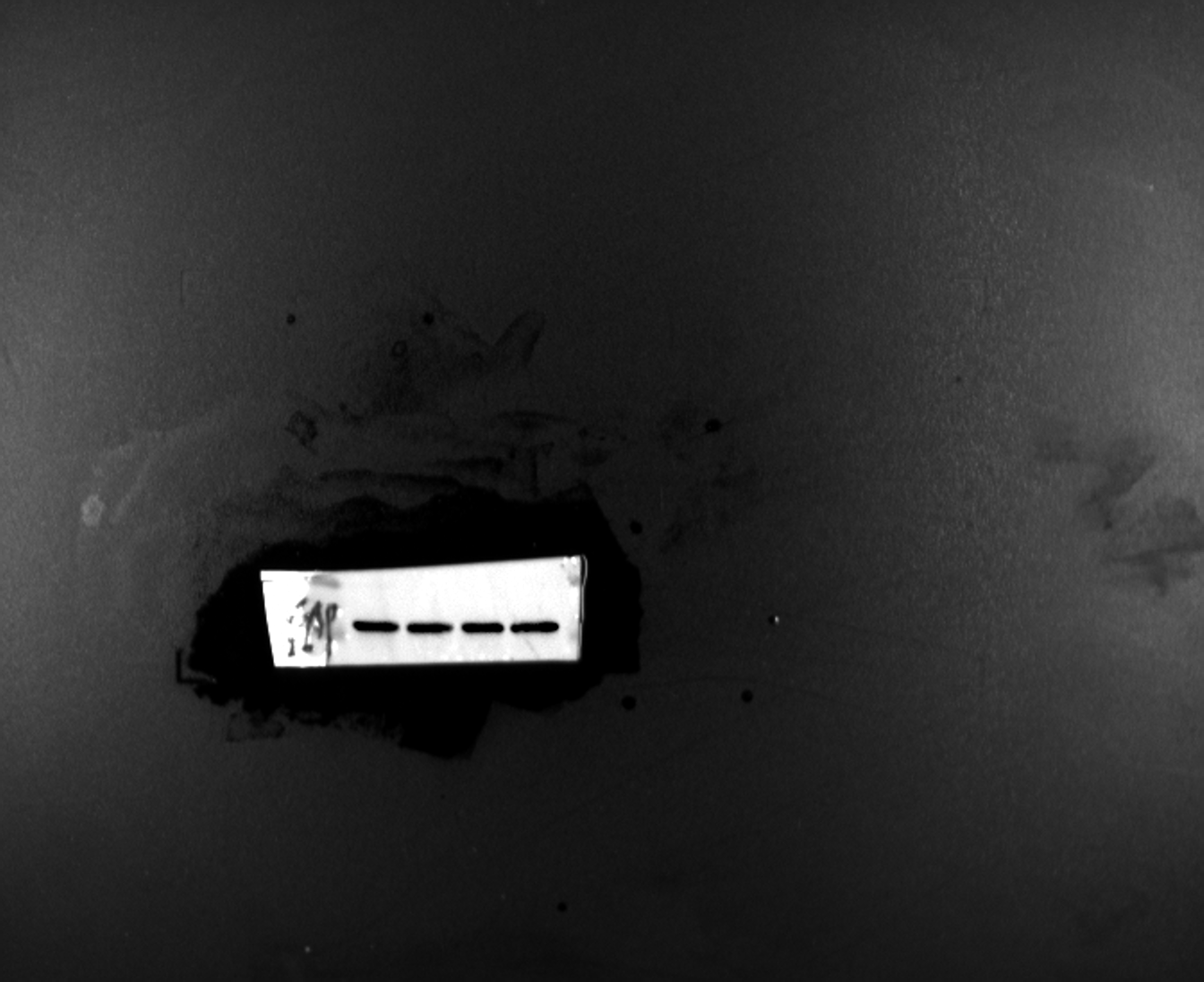

Supplement: Supplementary file 17 [file DataSheet6.ZIP › 5. WB-Fibrotic protein/GAPDH 2-2.Tif]

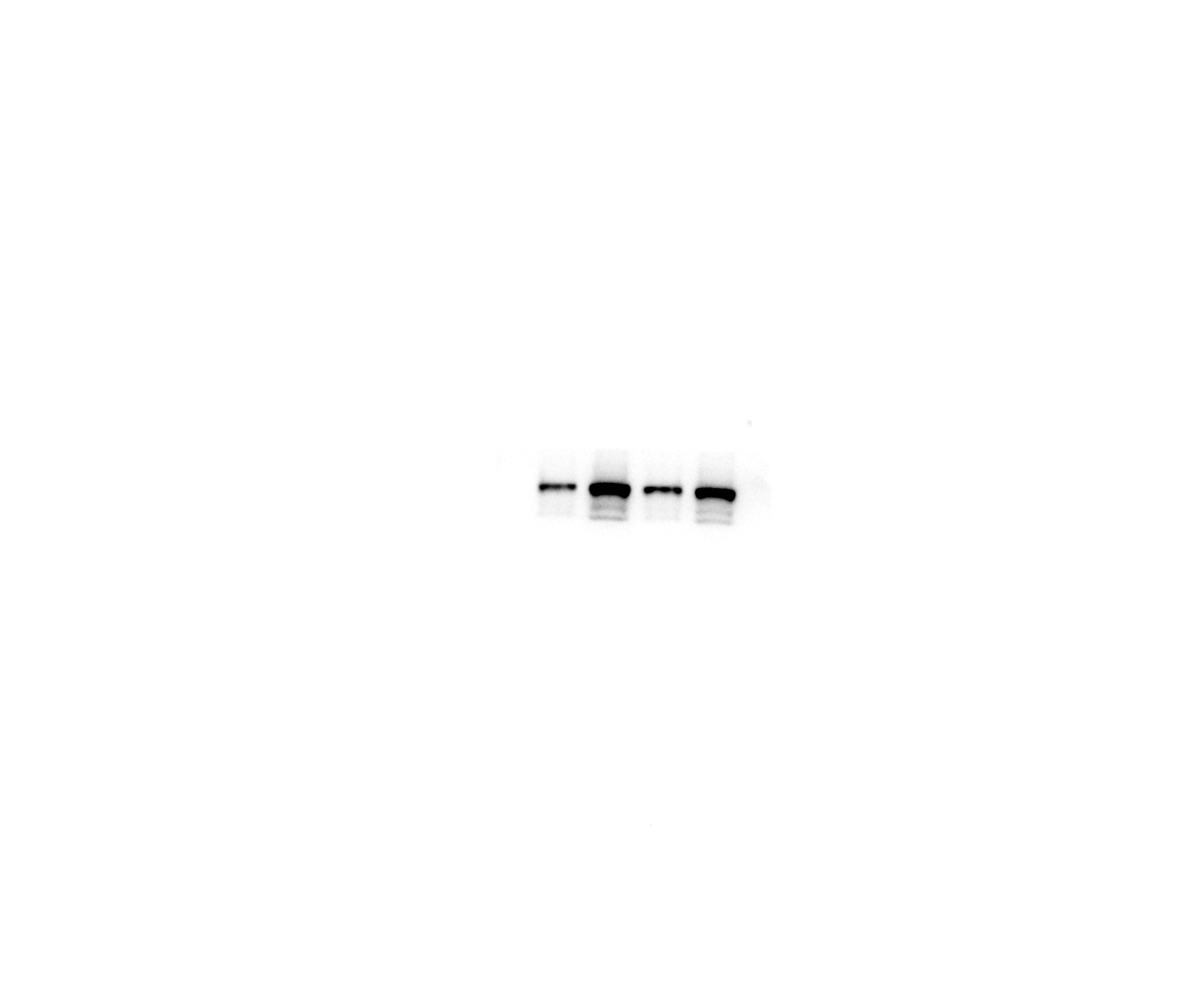

Supplement: Supplementary file 17 [file DataSheet6.ZIP › 5. WB-Fibrotic protein/N-cadherin 1-1.Tif]

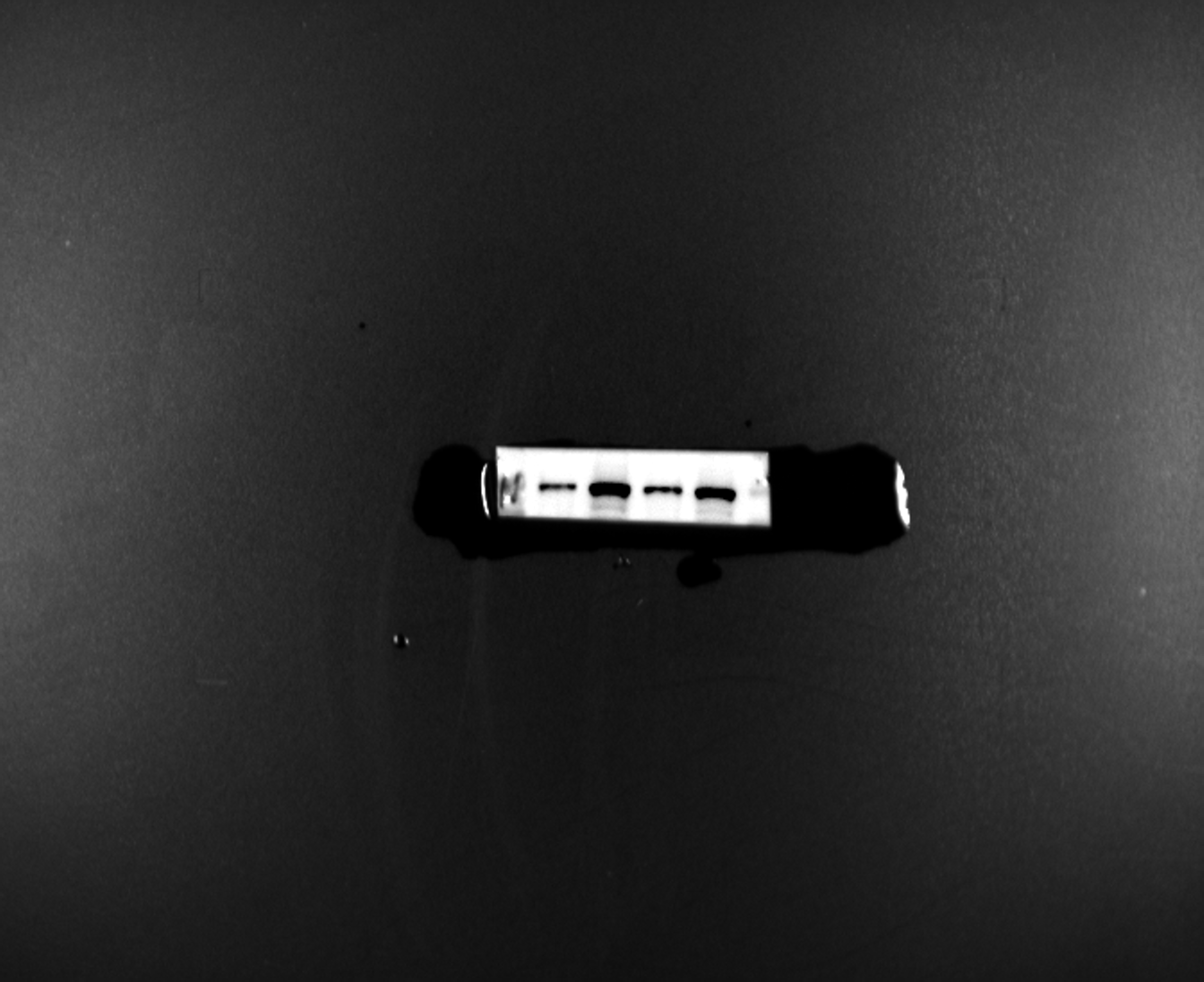

Supplement: Supplementary file 17 [file DataSheet6.ZIP › 5. WB-Fibrotic protein/N-cadherin 1-2.Tif]

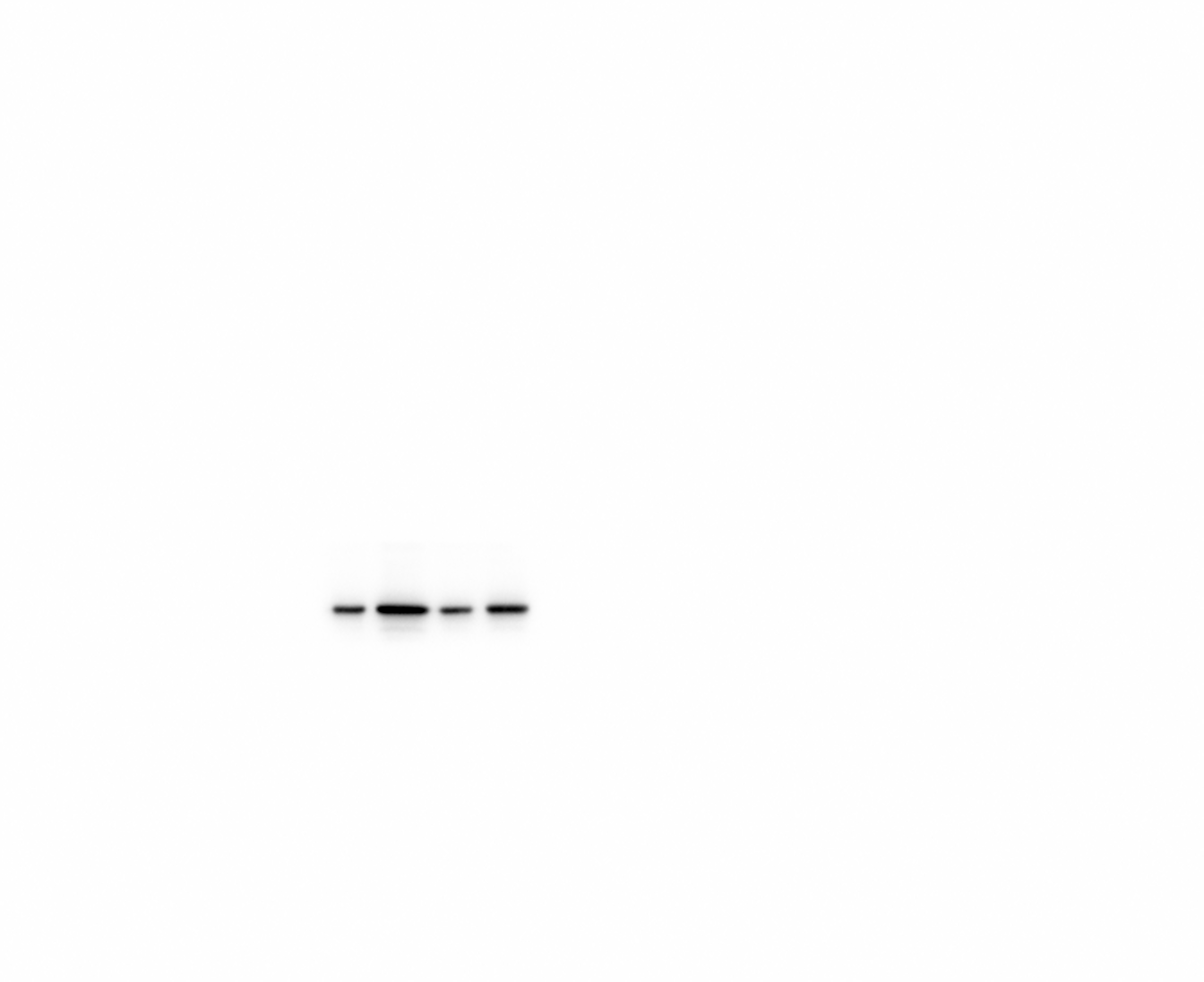

Supplement: Supplementary file 17 [file DataSheet6.ZIP › 5. WB-Fibrotic protein/Vimentin 2-1.Tif]

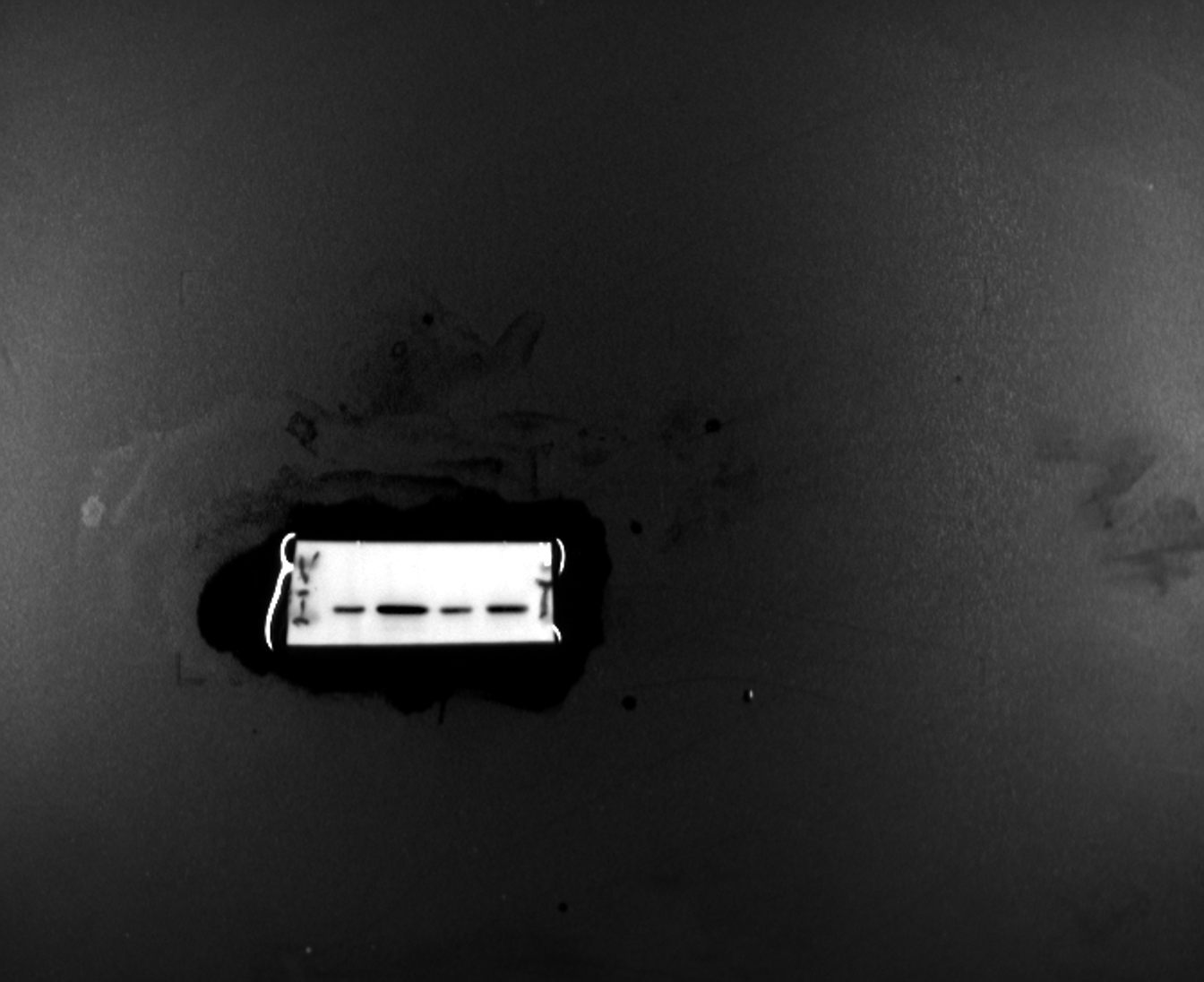

Supplement: Supplementary file 17 [file DataSheet6.ZIP › 5. WB-Fibrotic protein/Vimentin2-2.Tif]

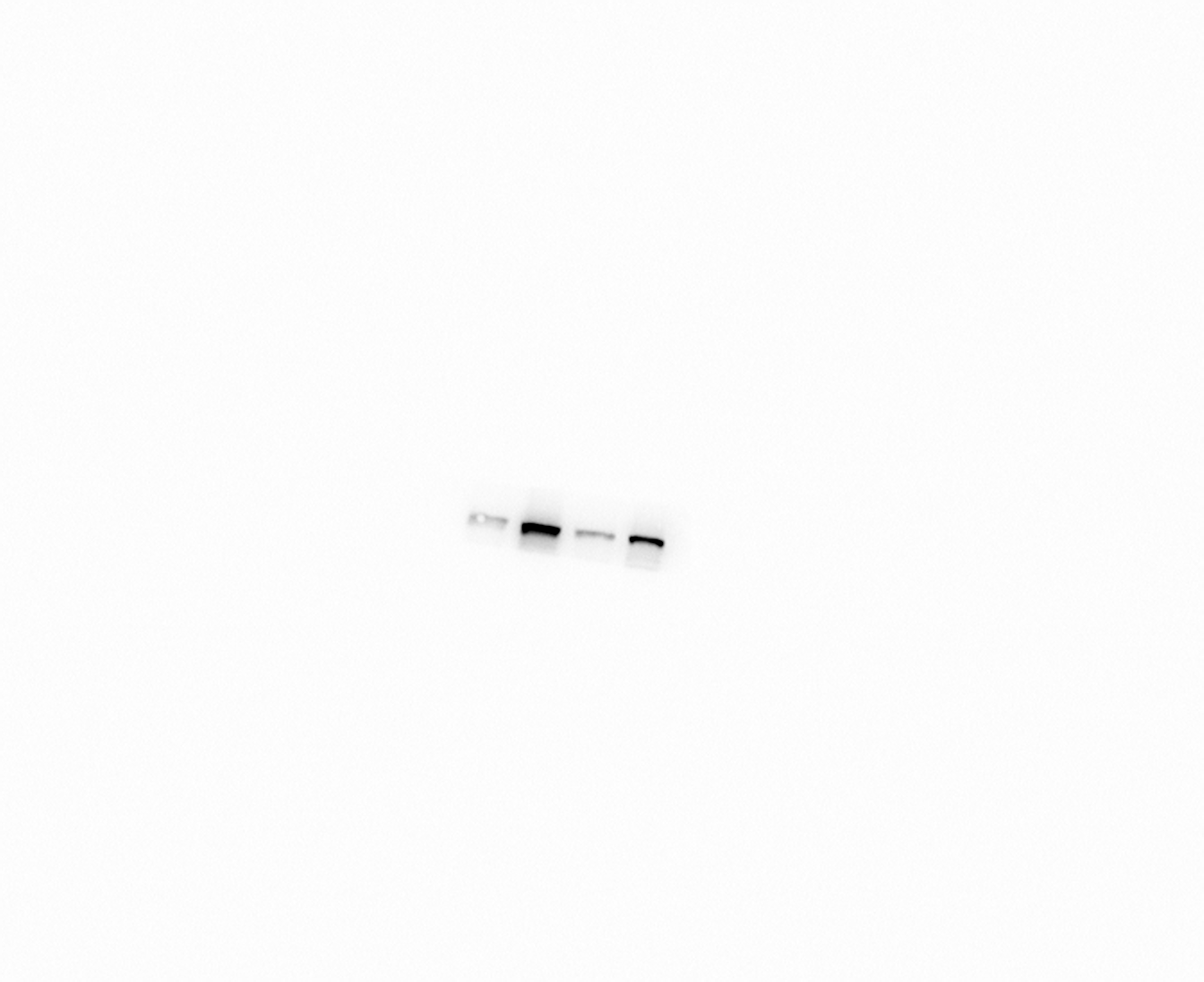

Supplement: Supplementary file 17 [file DataSheet6.ZIP › 5. WB-Fibrotic protein/sma 2-1.Tif]

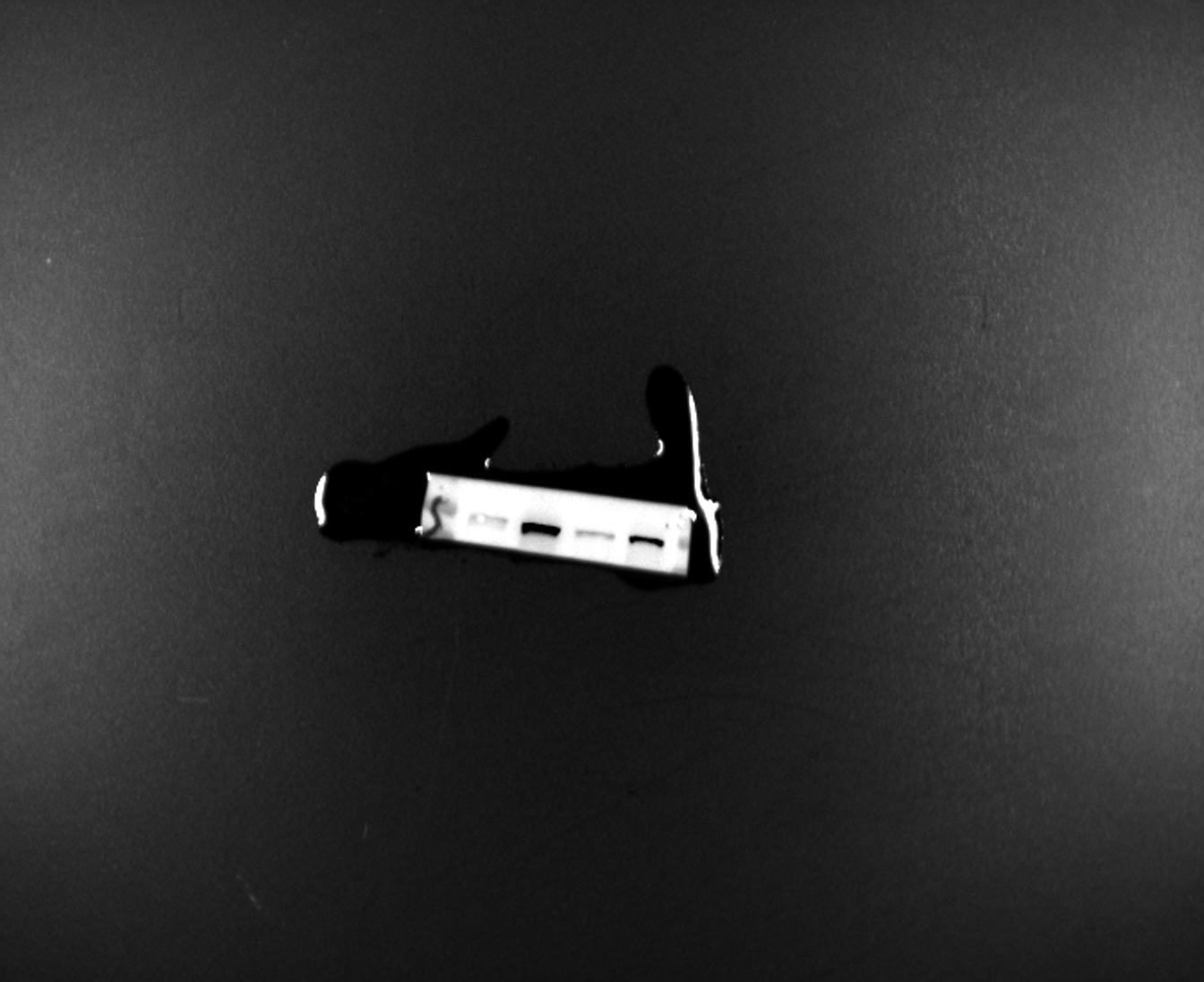

Supplement: Supplementary file 17 [file DataSheet6.ZIP › 5. WB-Fibrotic protein/sma 2-2.Tif]

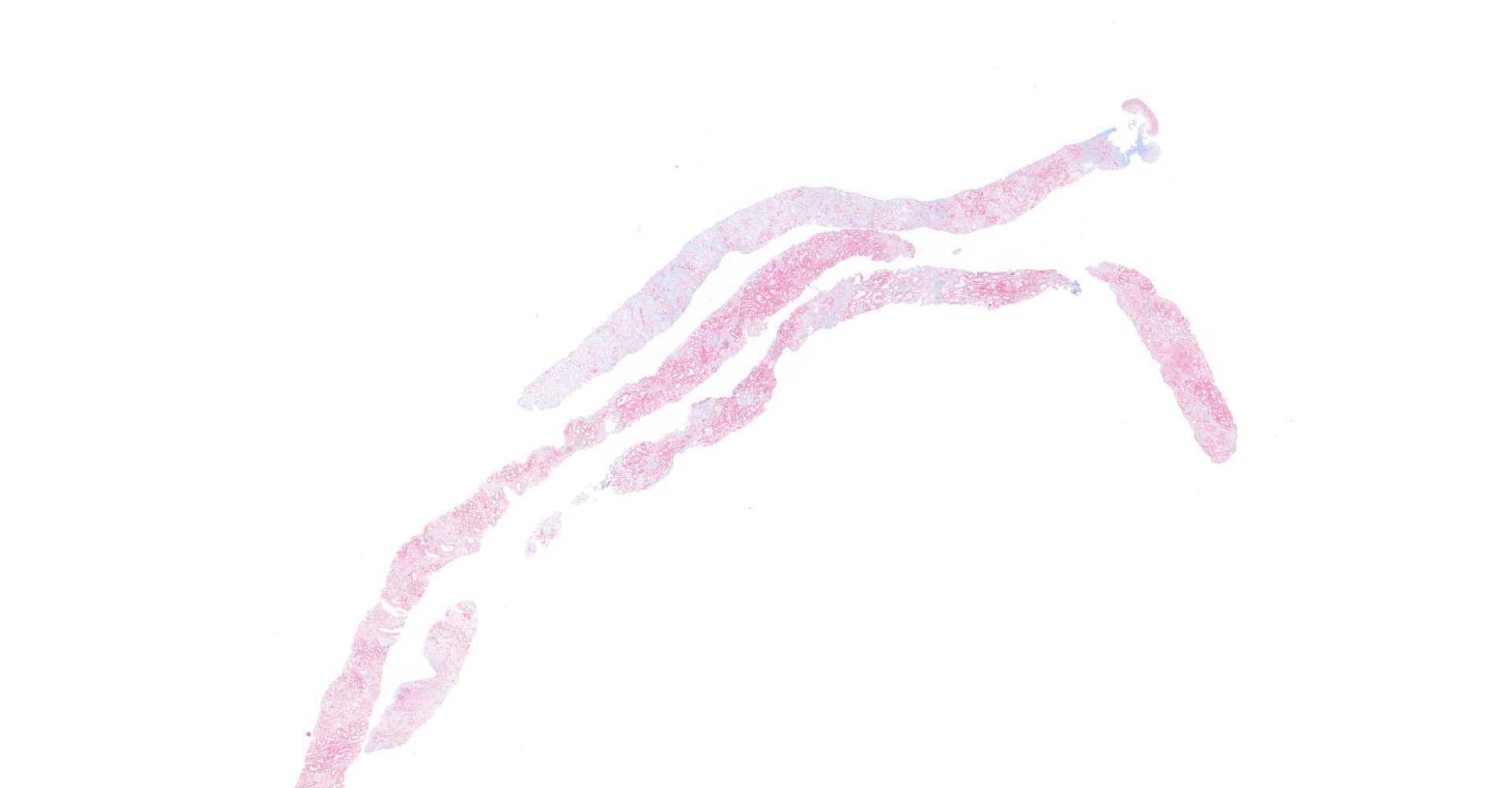

Supplement: Supplementary file 18 [file DataSheet12.ZIP › Masson staining of IgAN patients Full slice scan results/22-136_1.1x.tif]

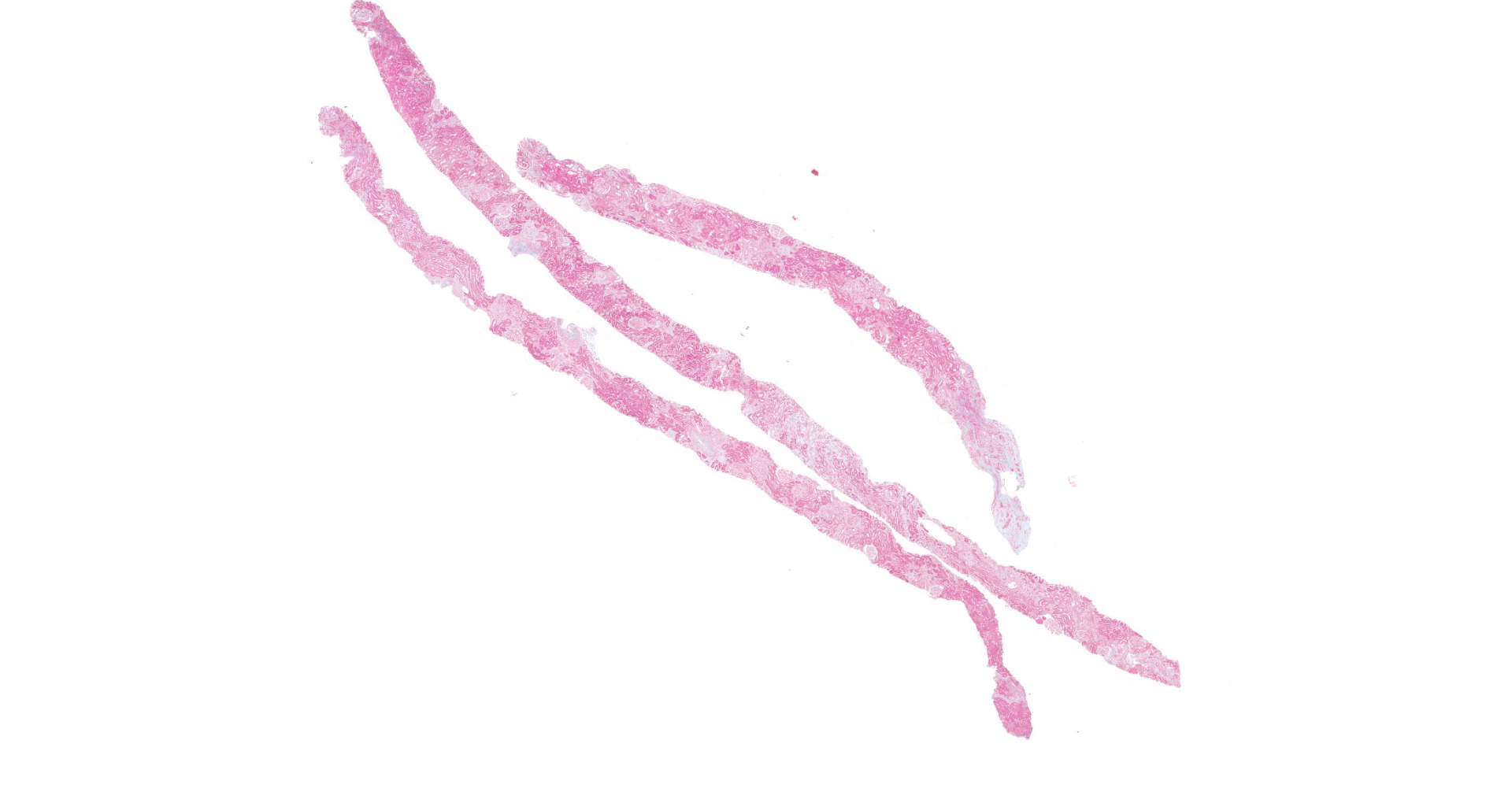

Supplement: Supplementary file 18 [file DataSheet12.ZIP › Masson staining of IgAN patients Full slice scan results/22-210_1.0x.tif]

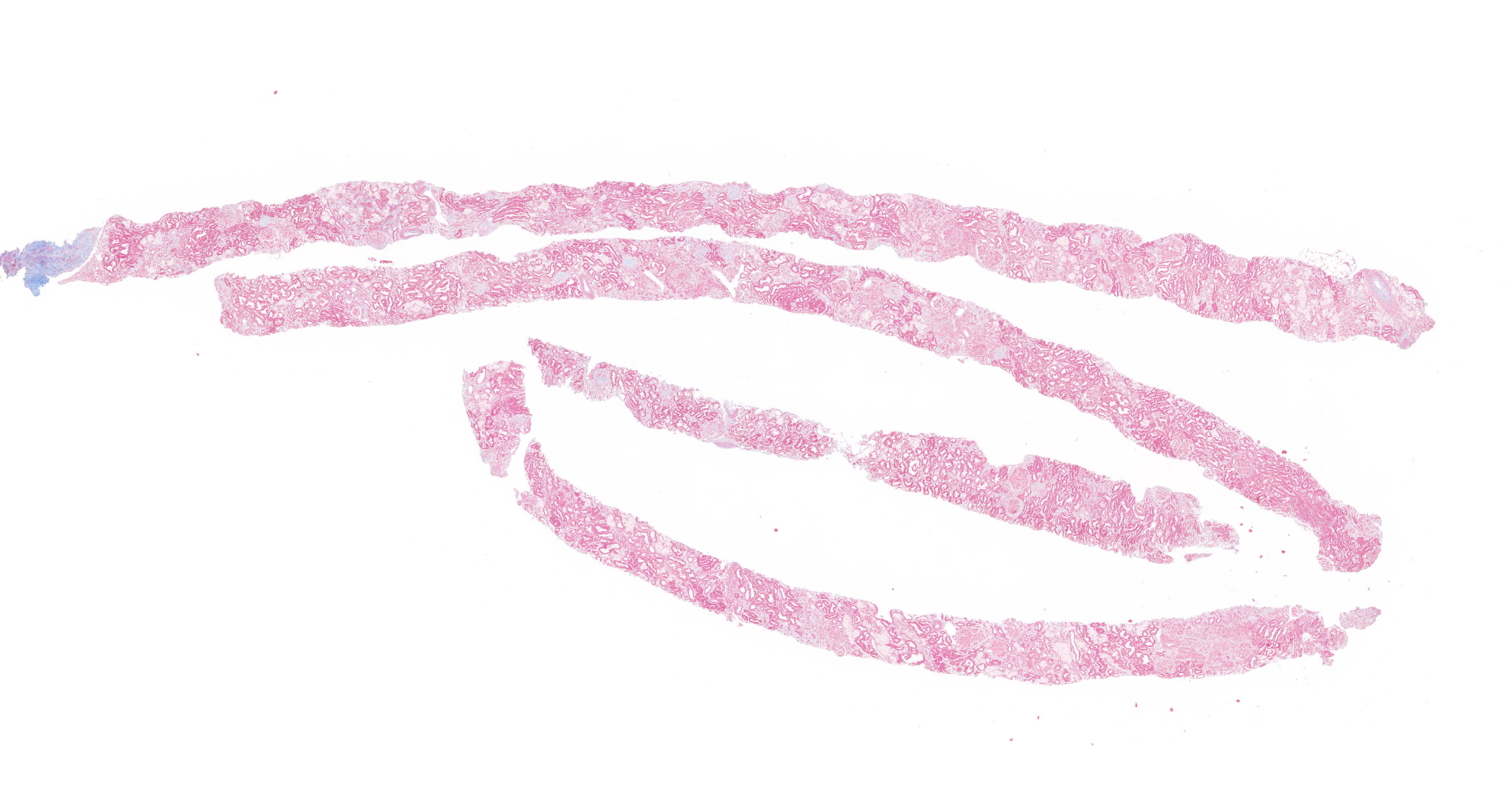

Supplement: Supplementary file 18 [file DataSheet12.ZIP › Masson staining of IgAN patients Full slice scan results/22-216_1.5x.jpg]

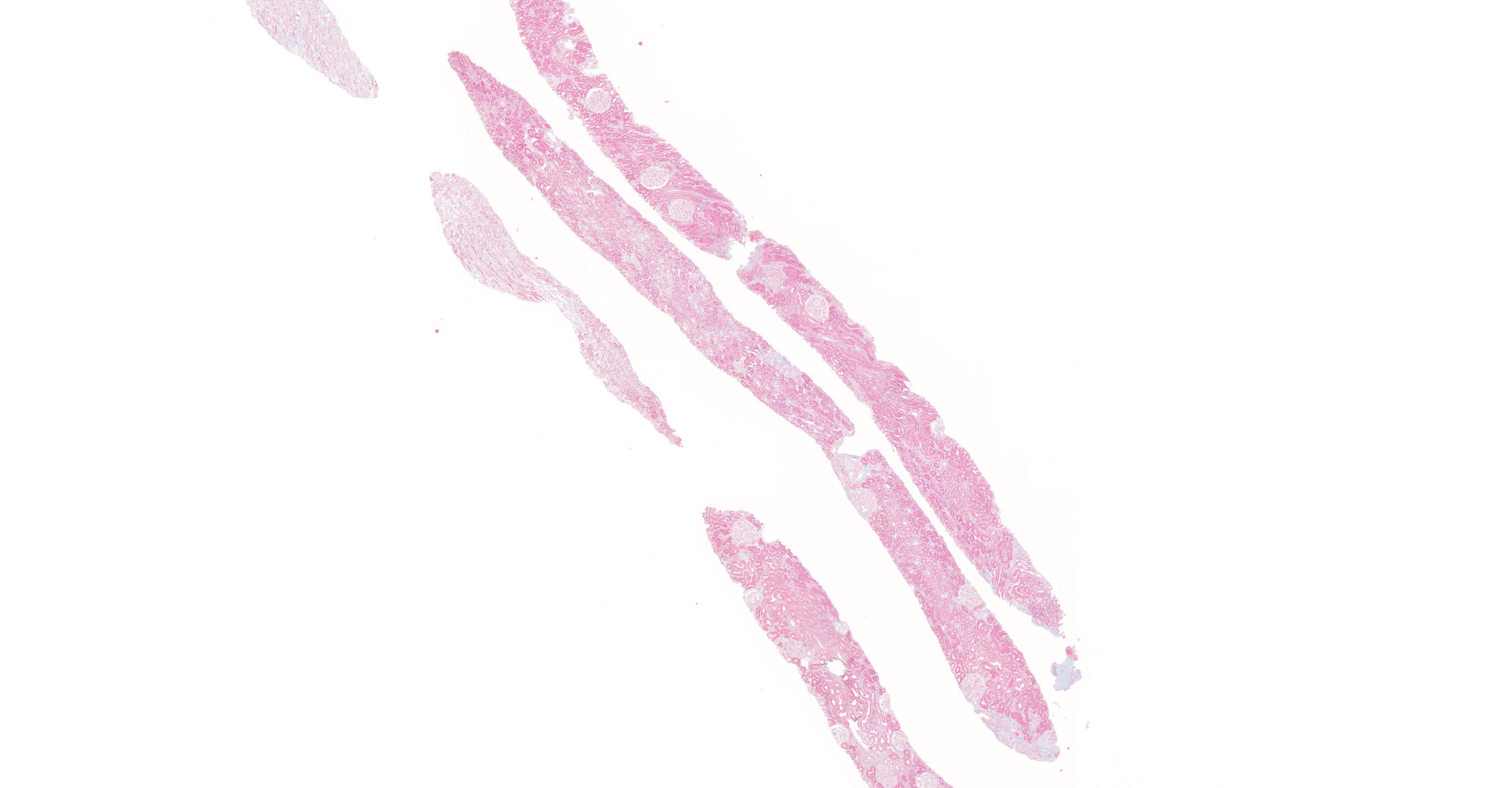

Supplement: Supplementary file 18 [file DataSheet12.ZIP › Masson staining of IgAN patients Full slice scan results/22-238_1.6x.tif]

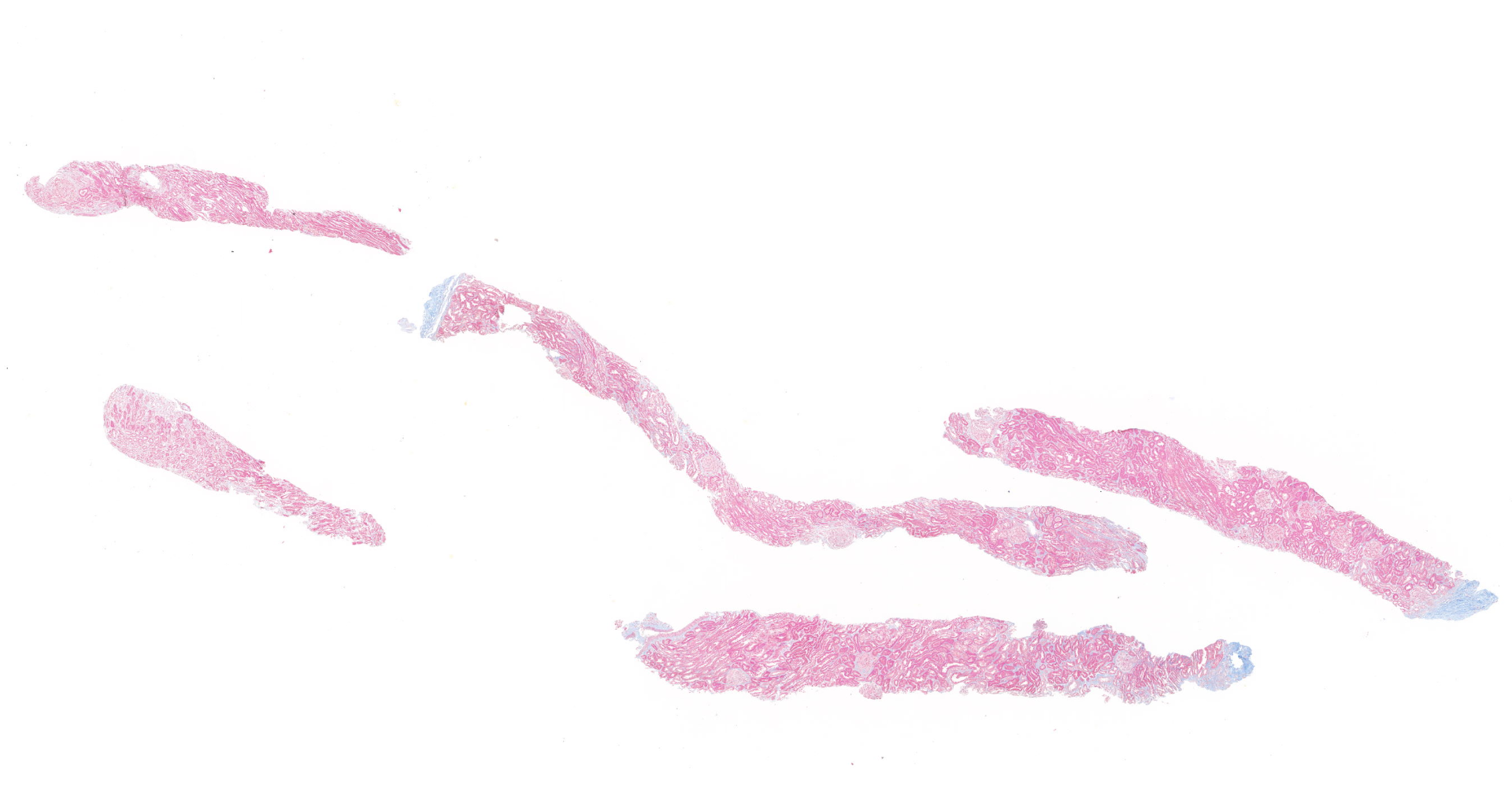

Supplement: Supplementary file 18 [file DataSheet12.ZIP › Masson staining of IgAN patients Full slice scan results/22-323_1.6x.tif]

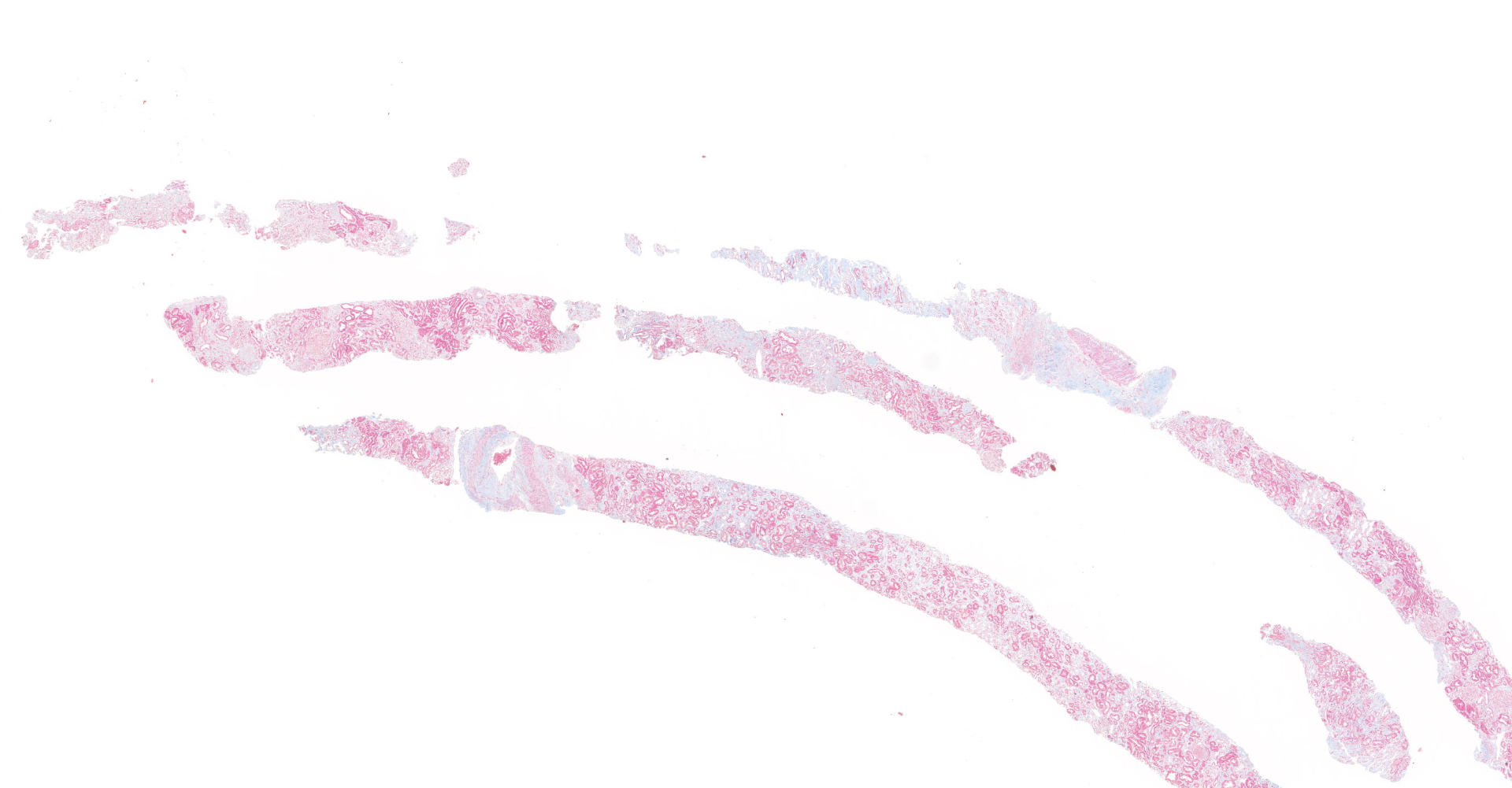

Supplement: Supplementary file 18 [file DataSheet12.ZIP › Masson staining of IgAN patients Full slice scan results/22-370_1.6x.tif]

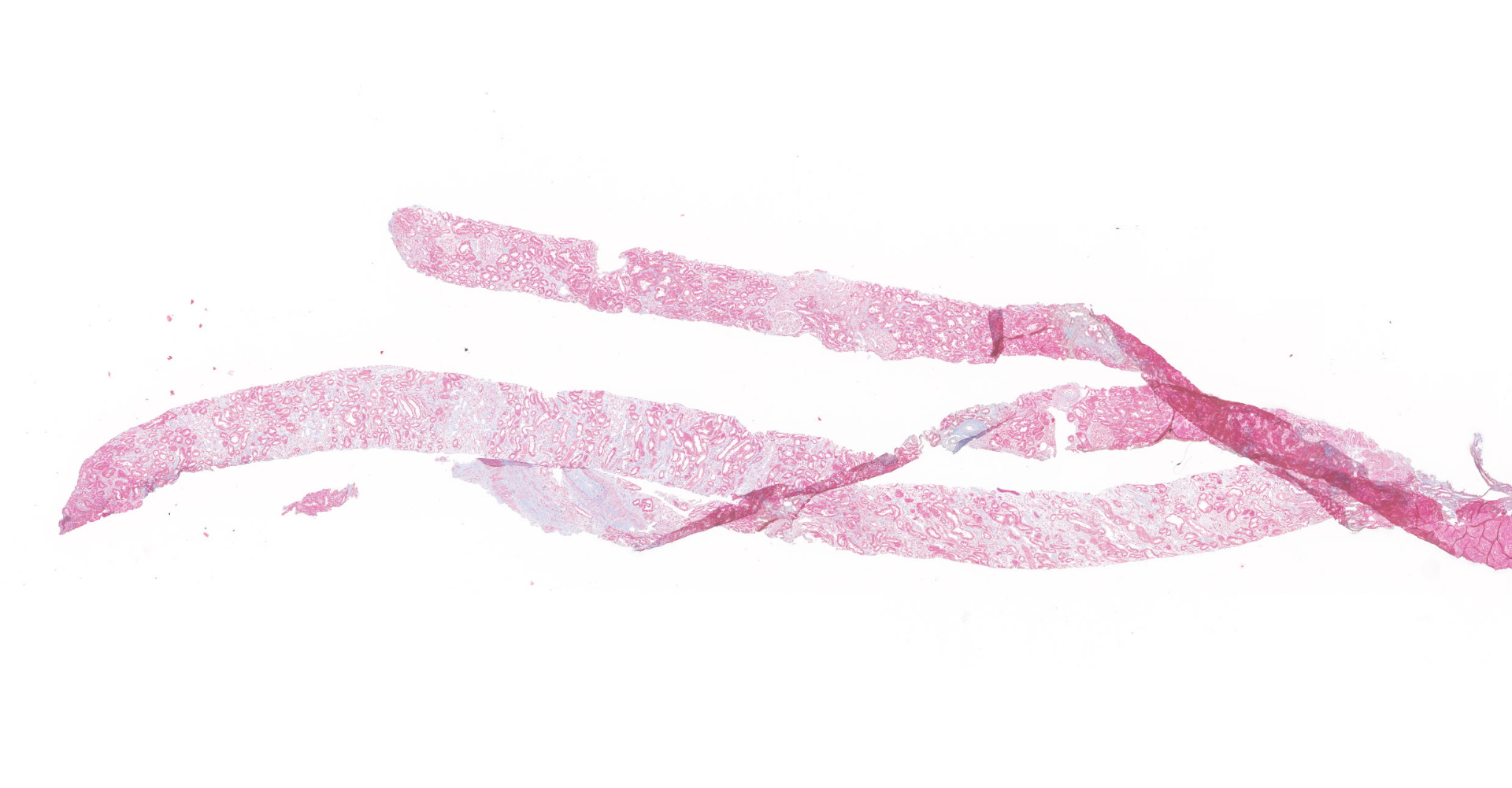

Supplement: Supplementary file 18 [file DataSheet12.ZIP › Masson staining of IgAN patients Full slice scan results/22-375_1.8x.tif]

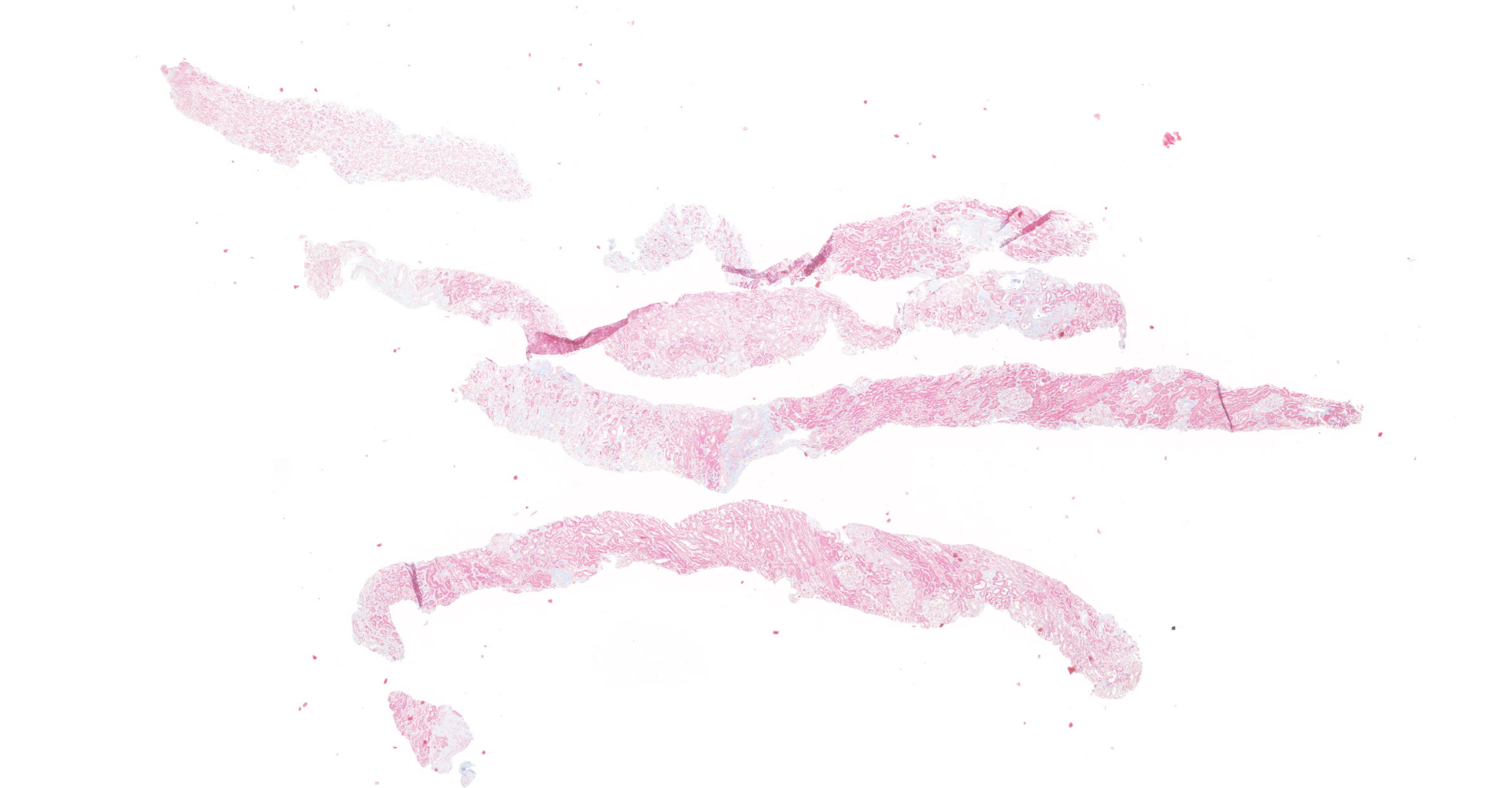

Supplement: Supplementary file 18 [file DataSheet12.ZIP › Masson staining of IgAN patients Full slice scan results/22-381_1.8x.tif]

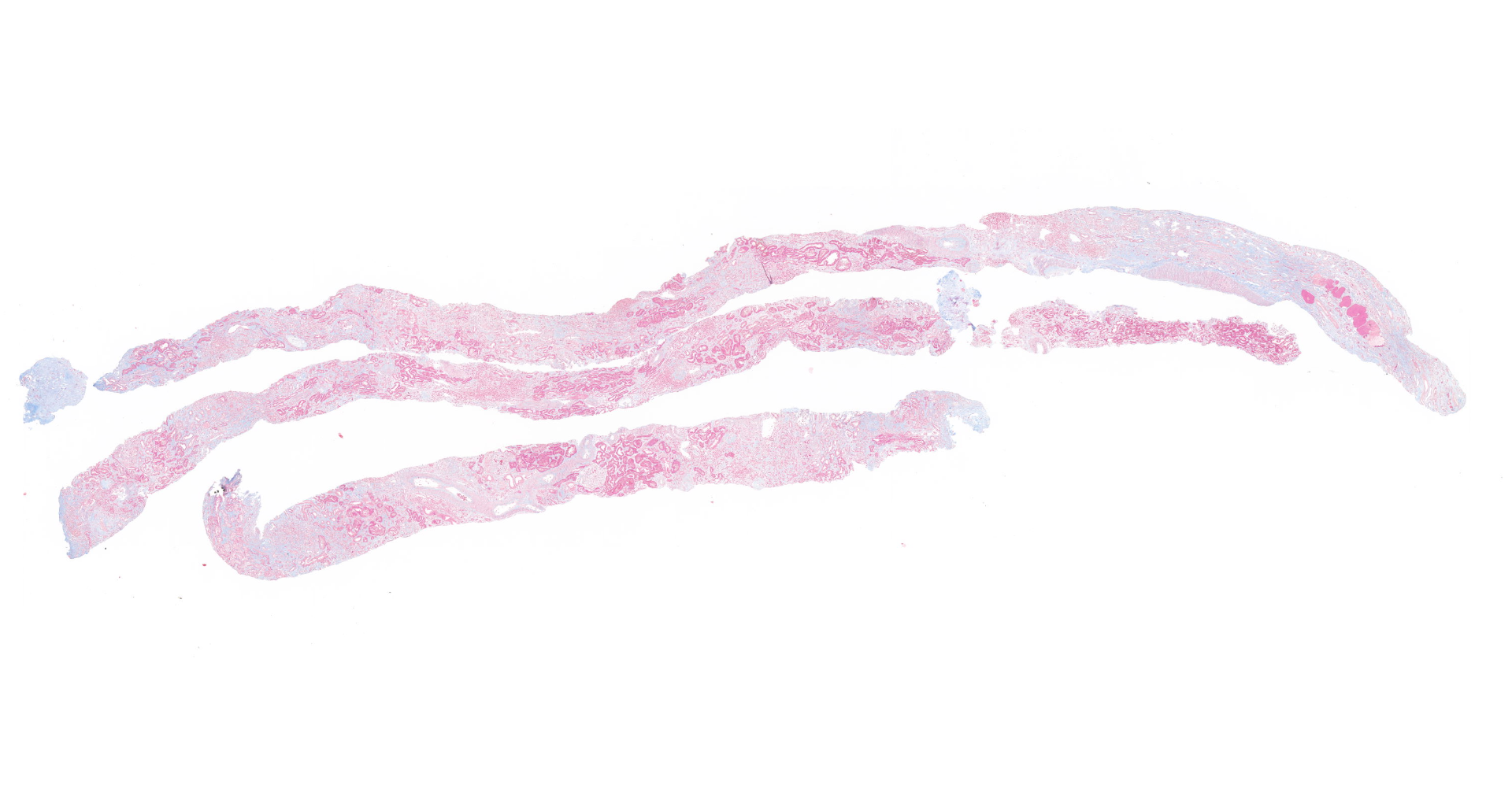

Supplement: Supplementary file 18 [file DataSheet12.ZIP › Masson staining of IgAN patients Full slice scan results/22-384_1.5x.tif]

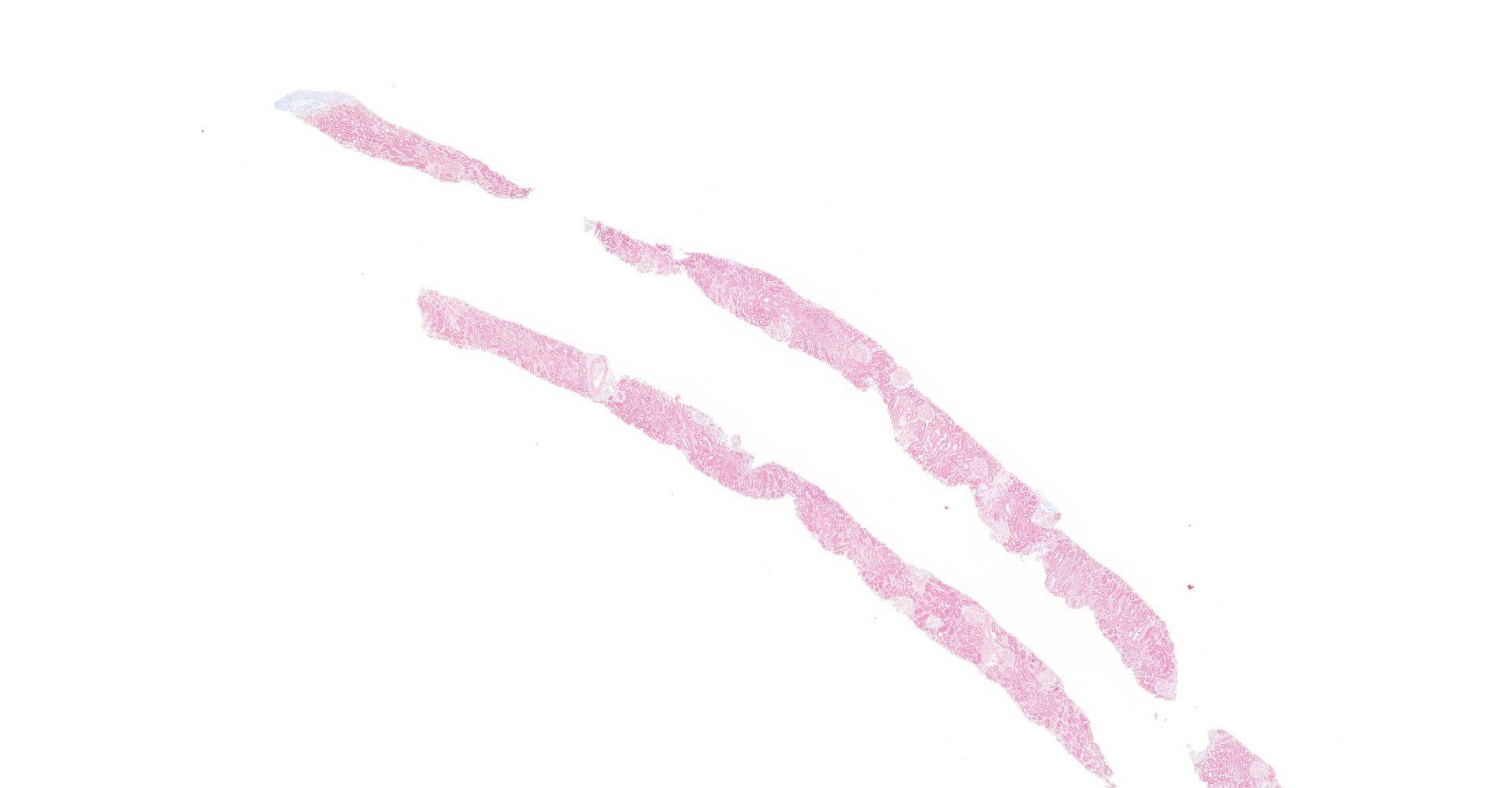

Supplement: Supplementary file 18 [file DataSheet12.ZIP › Masson staining of IgAN patients Full slice scan results/22-389_1.4x.tif]

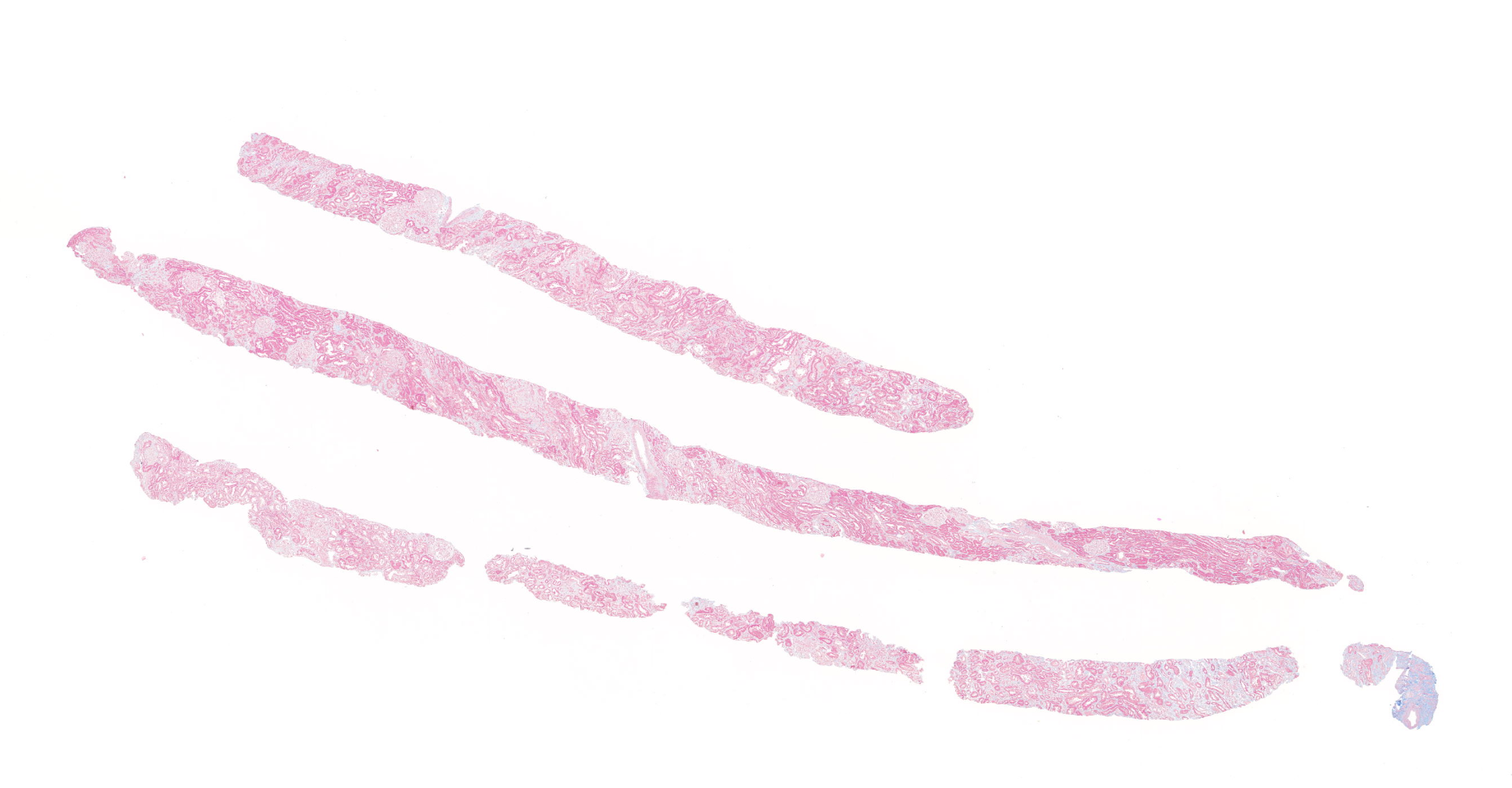

Supplement: Supplementary file 18 [file DataSheet12.ZIP › Masson staining of IgAN patients Full slice scan results/22-402_1.7x.bmp]

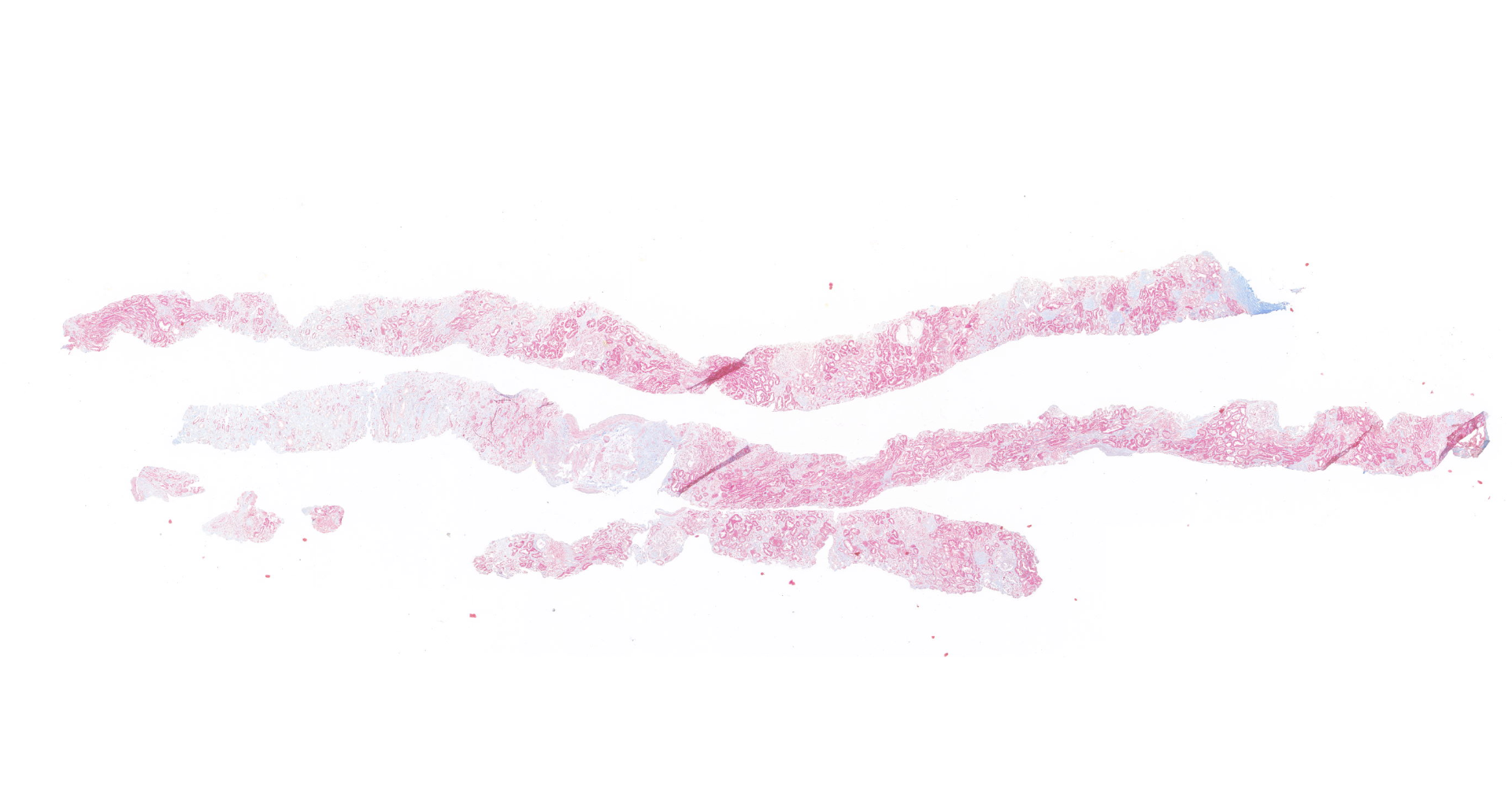

Supplement: Supplementary file 18 [file DataSheet12.ZIP › Masson staining of IgAN patients Full slice scan results/22-424_1.6x.tif]

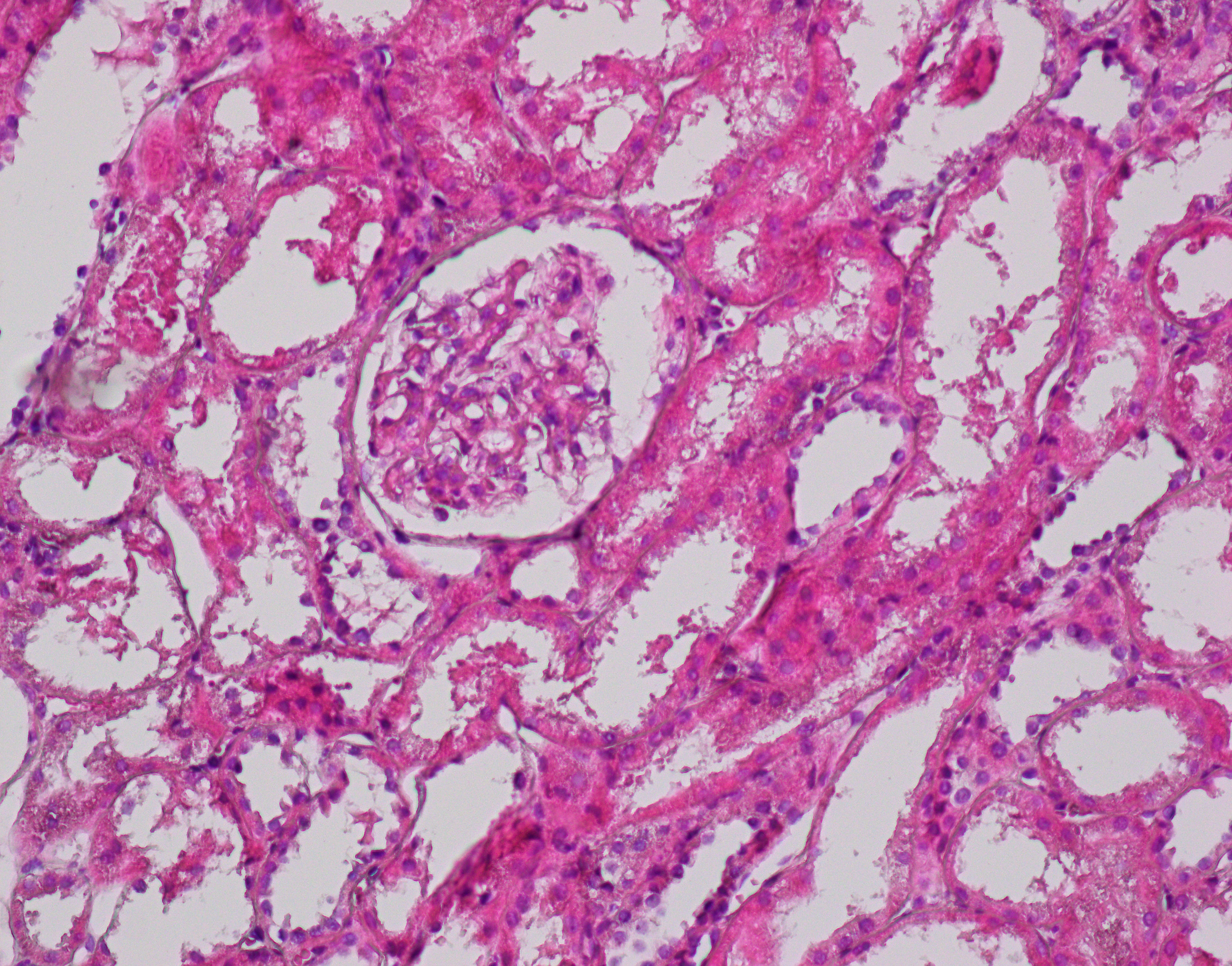

Supplement: Supplementary file 19 [file DataSheet2.ZIP › 1. IgAN patients HE stain/═╝╧±_30638.bmp]

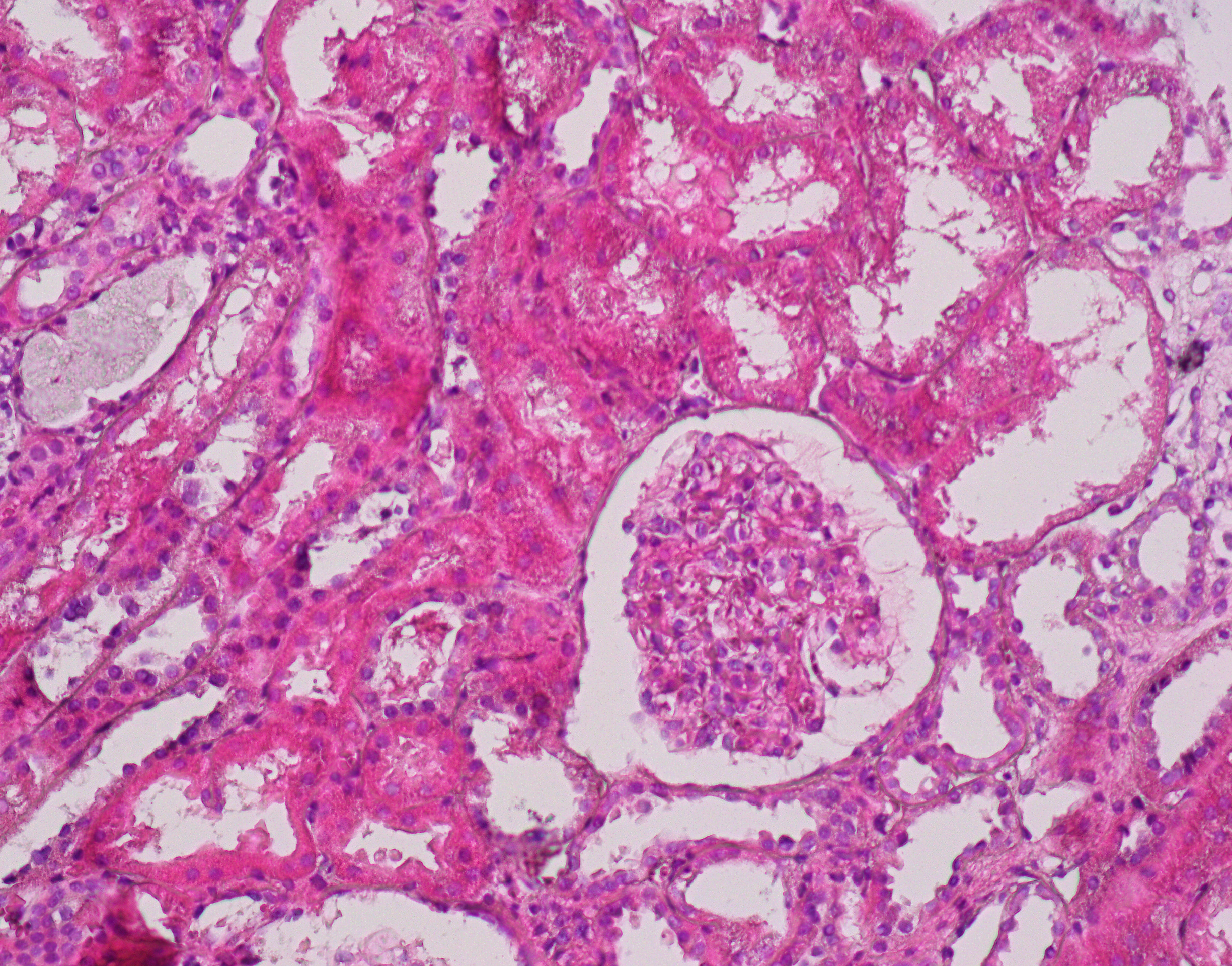

Supplement: Supplementary file 19 [file DataSheet2.ZIP › 1. IgAN patients HE stain/═╝╧±_30639.bmp]

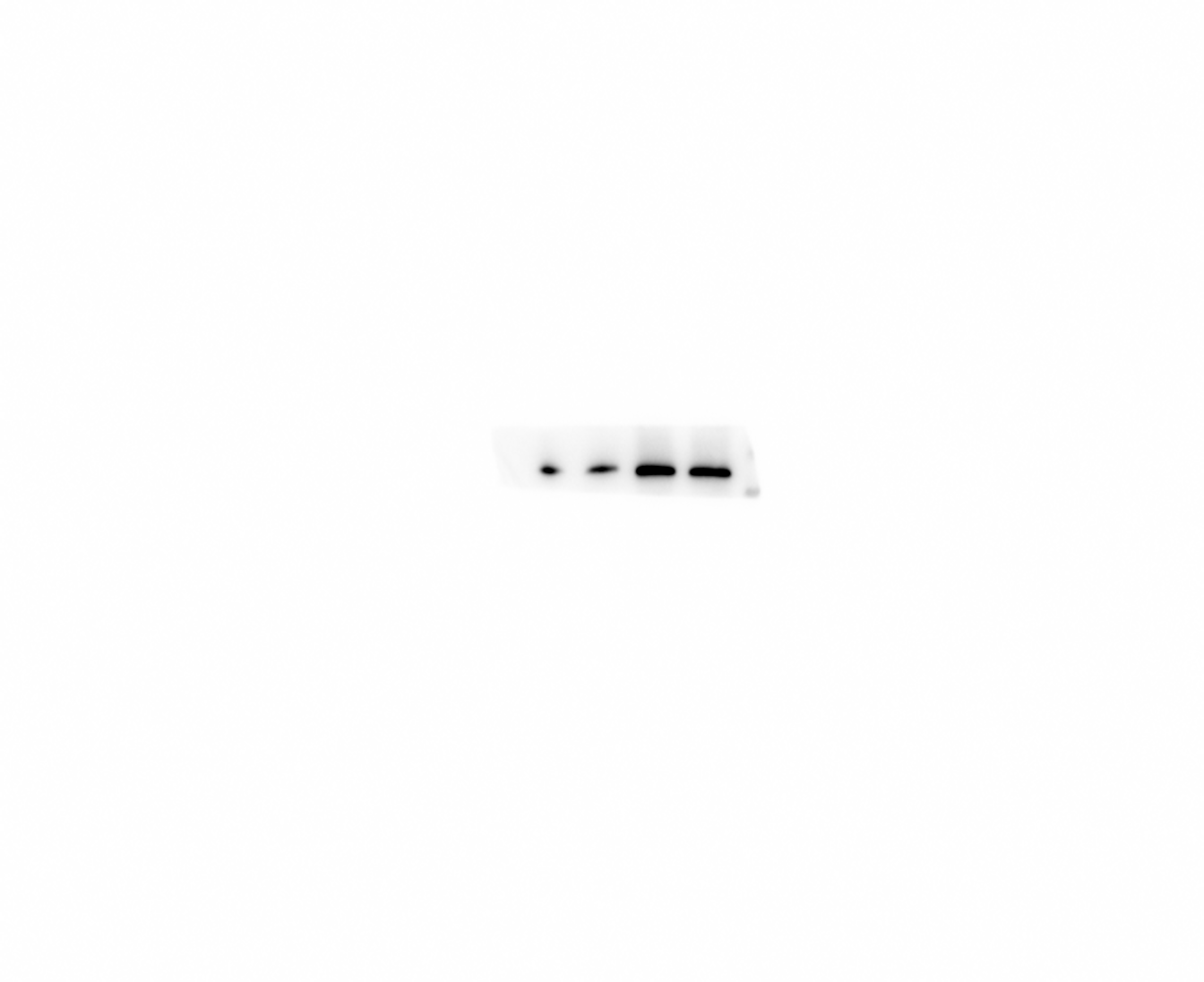

Supplement: Supplementary file 21 [file DataSheet5.ZIP › 4.WB APOC1 in HK-2 cell/APOC1.Tif]

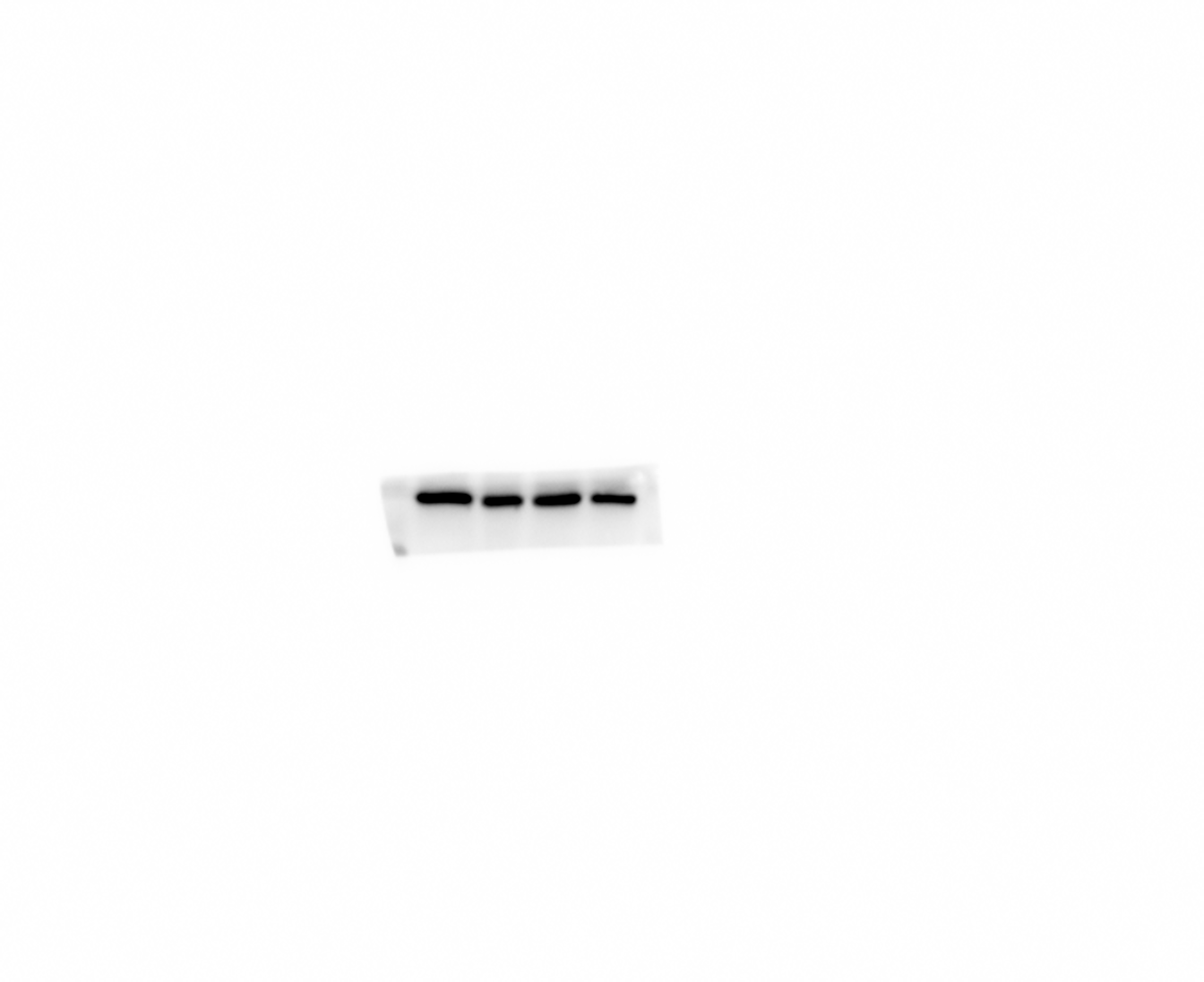

Supplement: Supplementary file 21 [file DataSheet5.ZIP › 4.WB APOC1 in HK-2 cell/GAPDH.Tif]

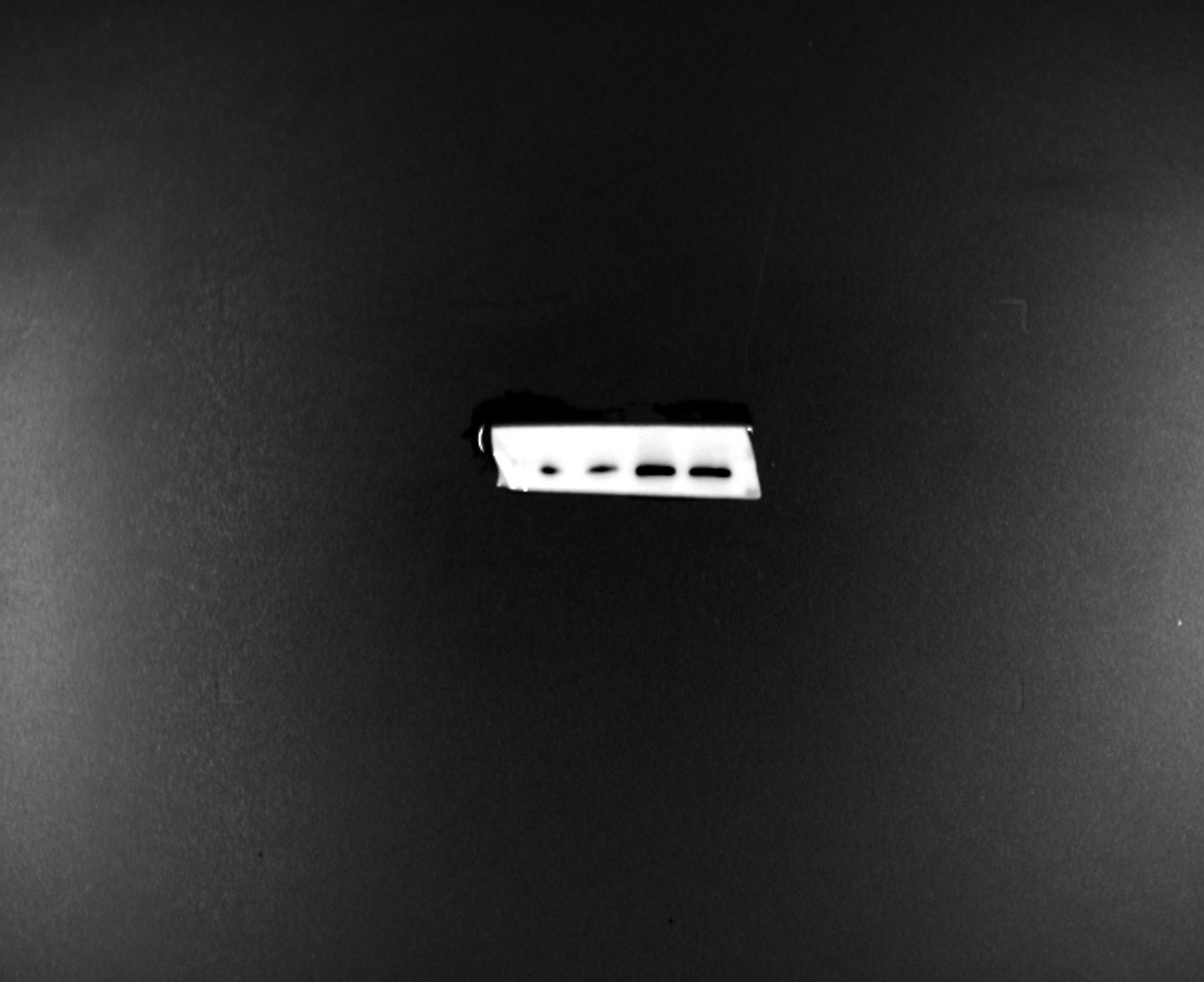

Supplement: Supplementary file 21 [file DataSheet5.ZIP › 4.WB APOC1 in HK-2 cell/original APOC1 .Tif]

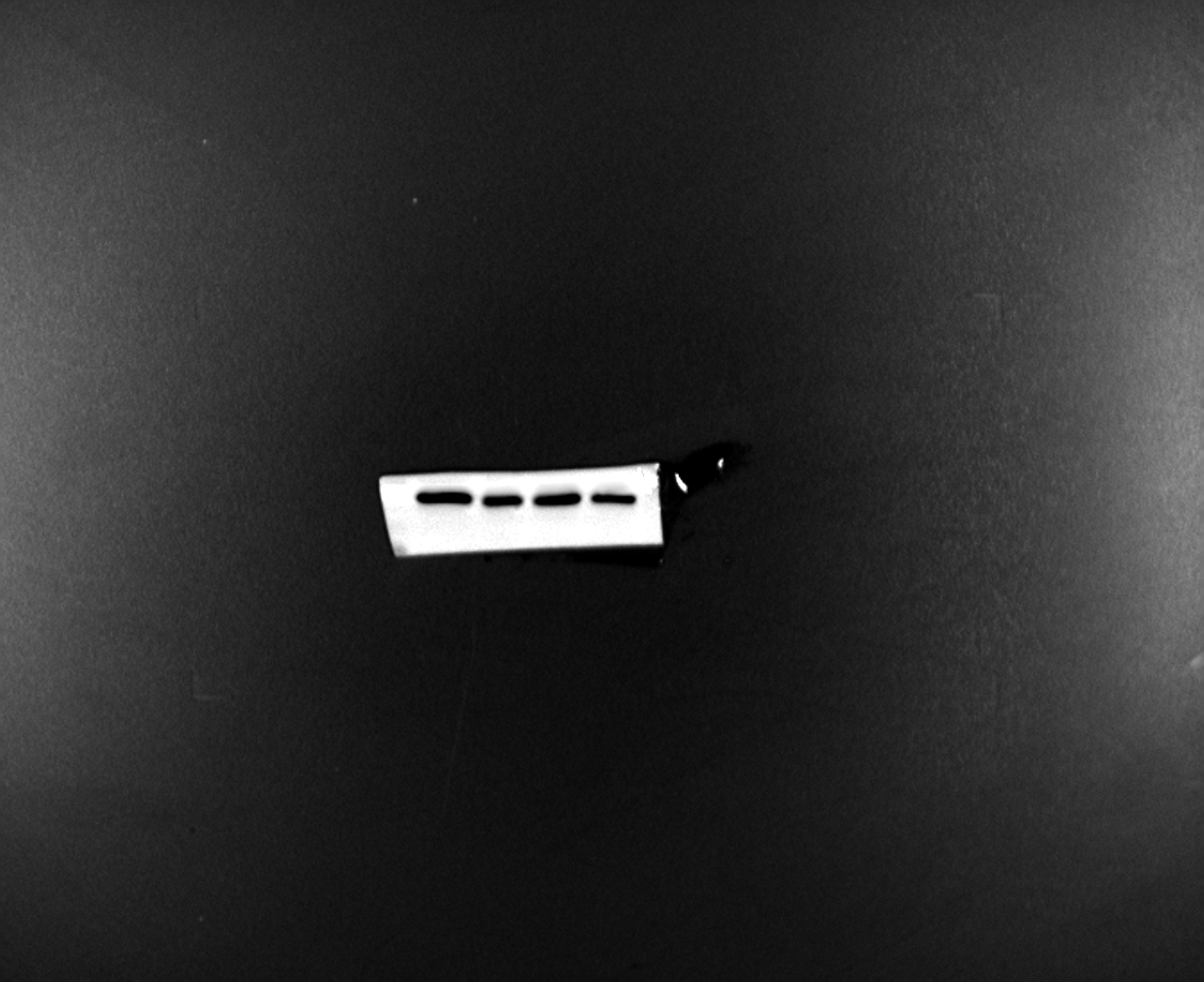

Supplement: Supplementary file 21 [file DataSheet5.ZIP › 4.WB APOC1 in HK-2 cell/original GAPDH.Tif]

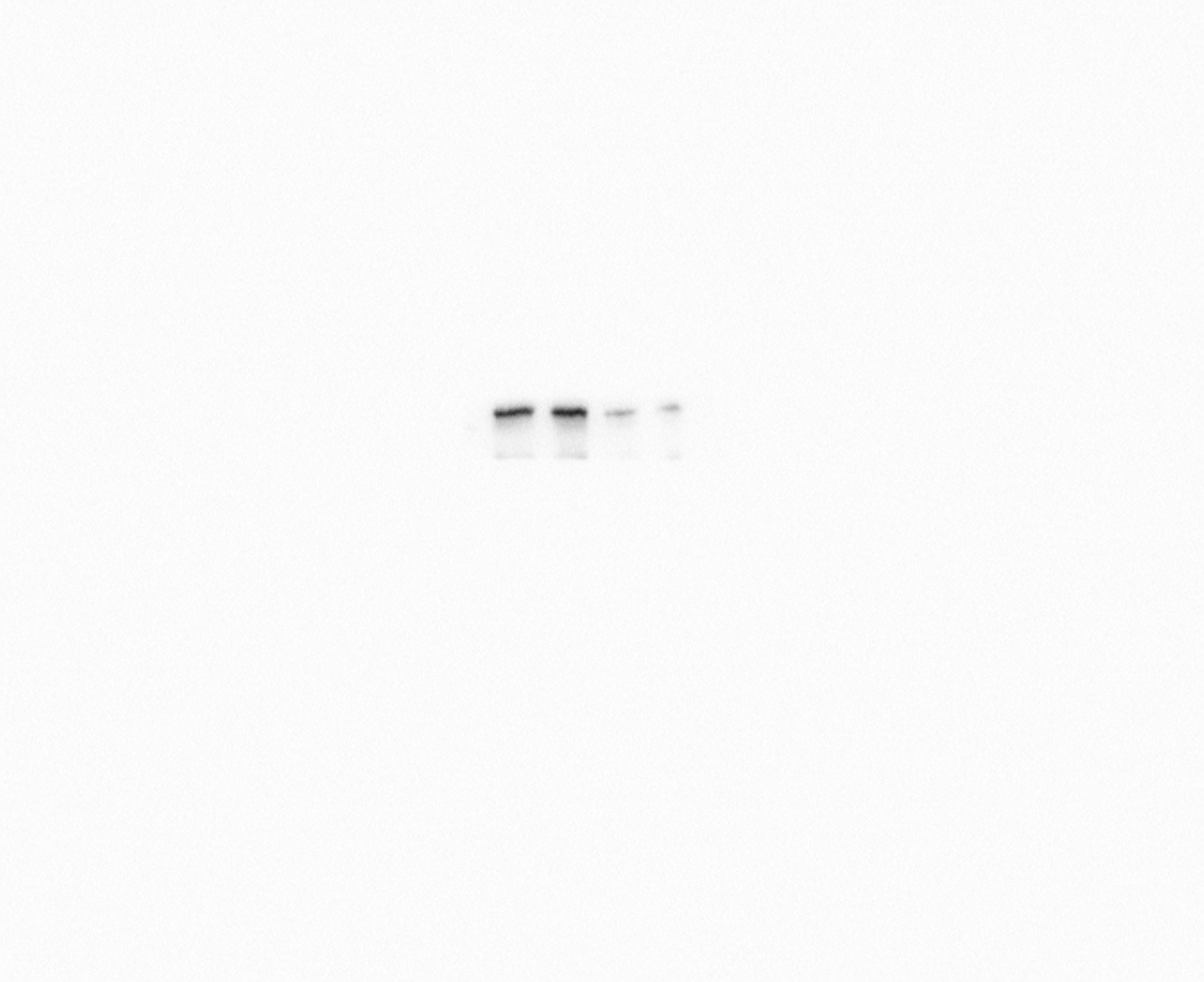

Supplement: Supplementary file 22 [file DataSheet7.ZIP › 6.WB Si-APOC1/APOC1 1-1.Tif]

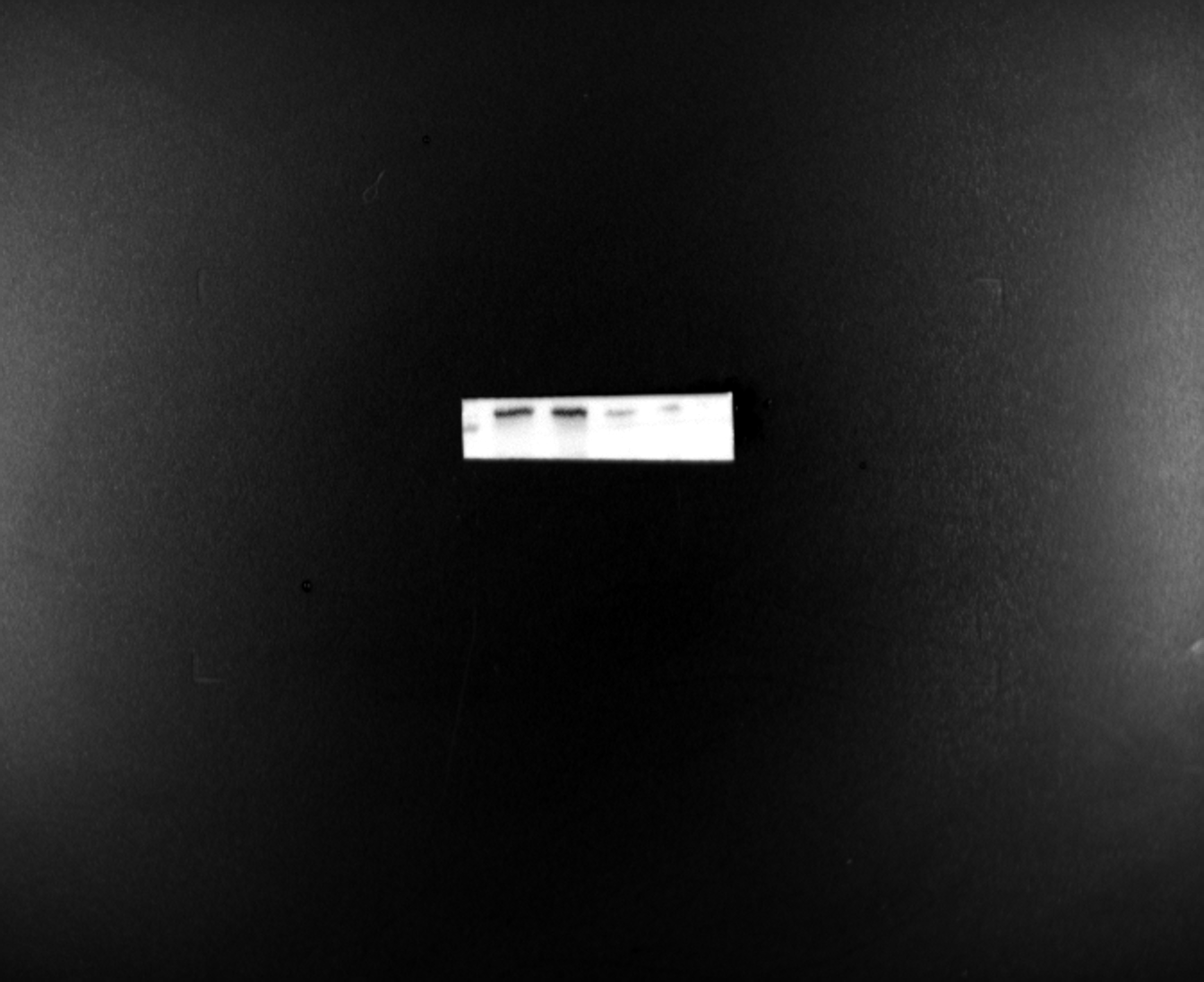

Supplement: Supplementary file 22 [file DataSheet7.ZIP › 6.WB Si-APOC1/APOC1 1-2.Tif]

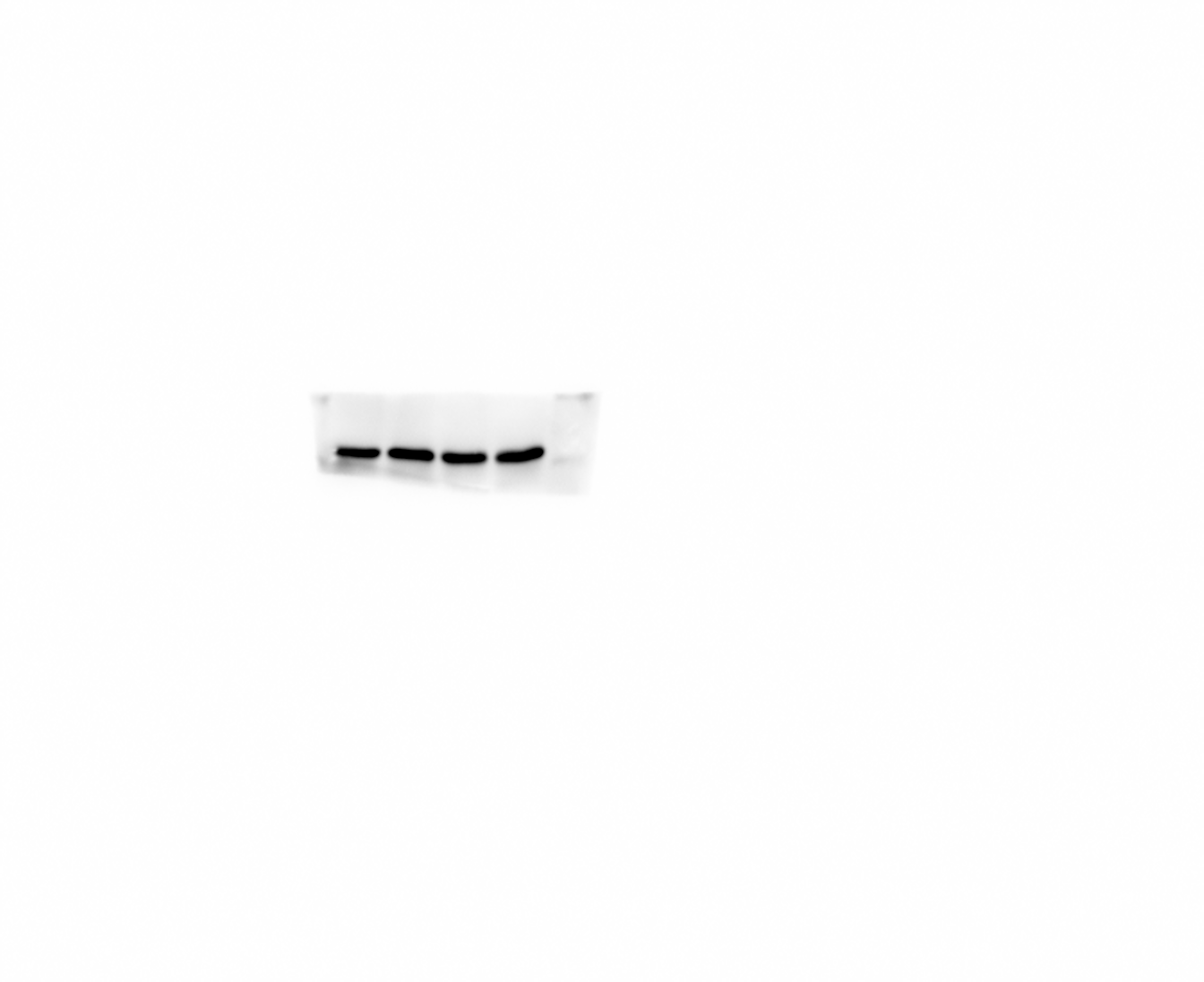

Supplement: Supplementary file 22 [file DataSheet7.ZIP › 6.WB Si-APOC1/GAPDH 5-1.Tif]

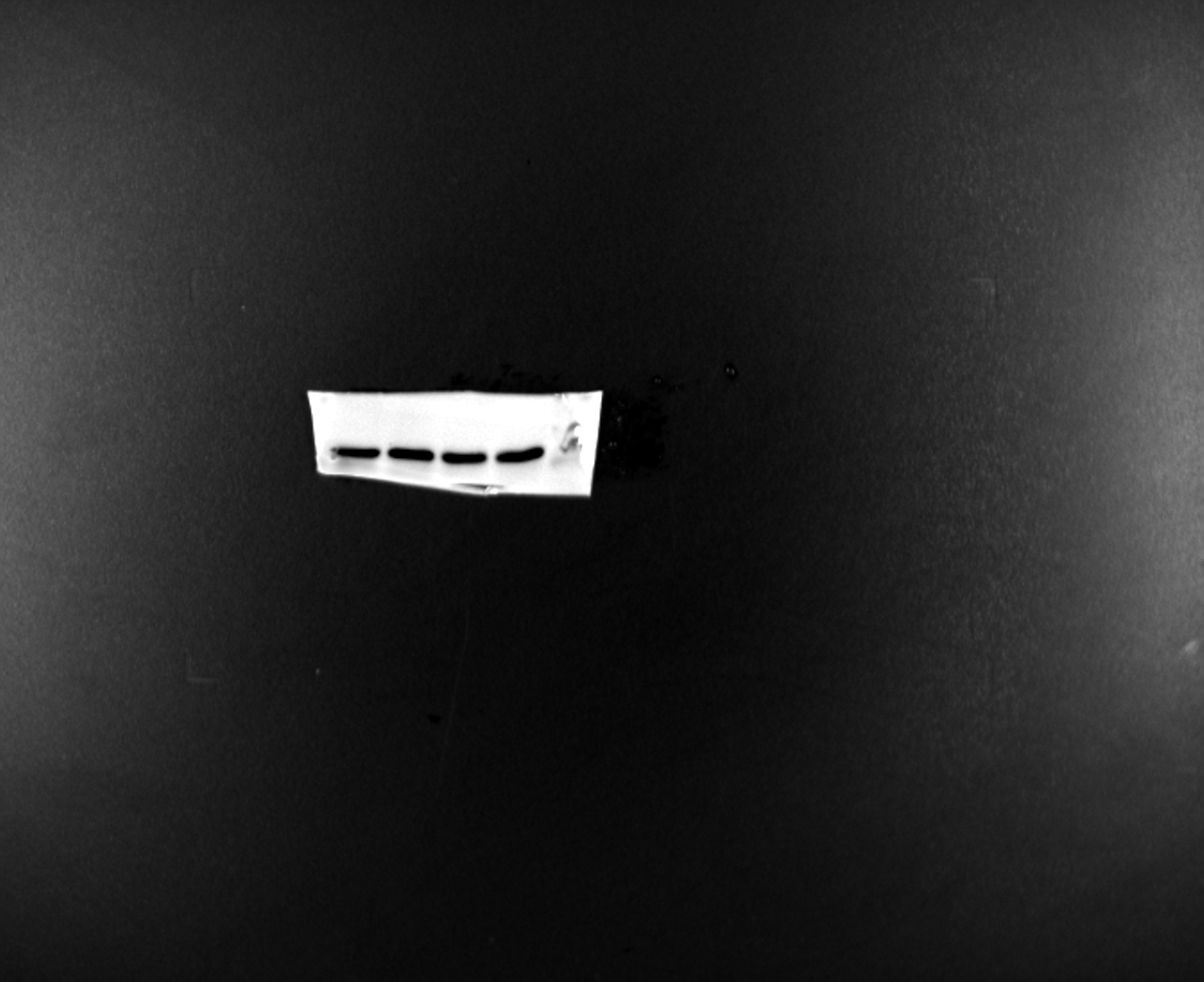

Supplement: Supplementary file 22 [file DataSheet7.ZIP › 6.WB Si-APOC1/GAPDH 5-2.Tif]
