# Supplementary material for: APOC1 exacerbates renal fibrosis through the activation of the NF-κB signaling pathway in IgAN
Source: Front Pharmacol. 2023 May 25;14:1181435. doi: 10.3389/fphar.2023.1181435 (PMC10248024; doi:10.3389/fphar.2023.1181435)

**Supplementary materials**


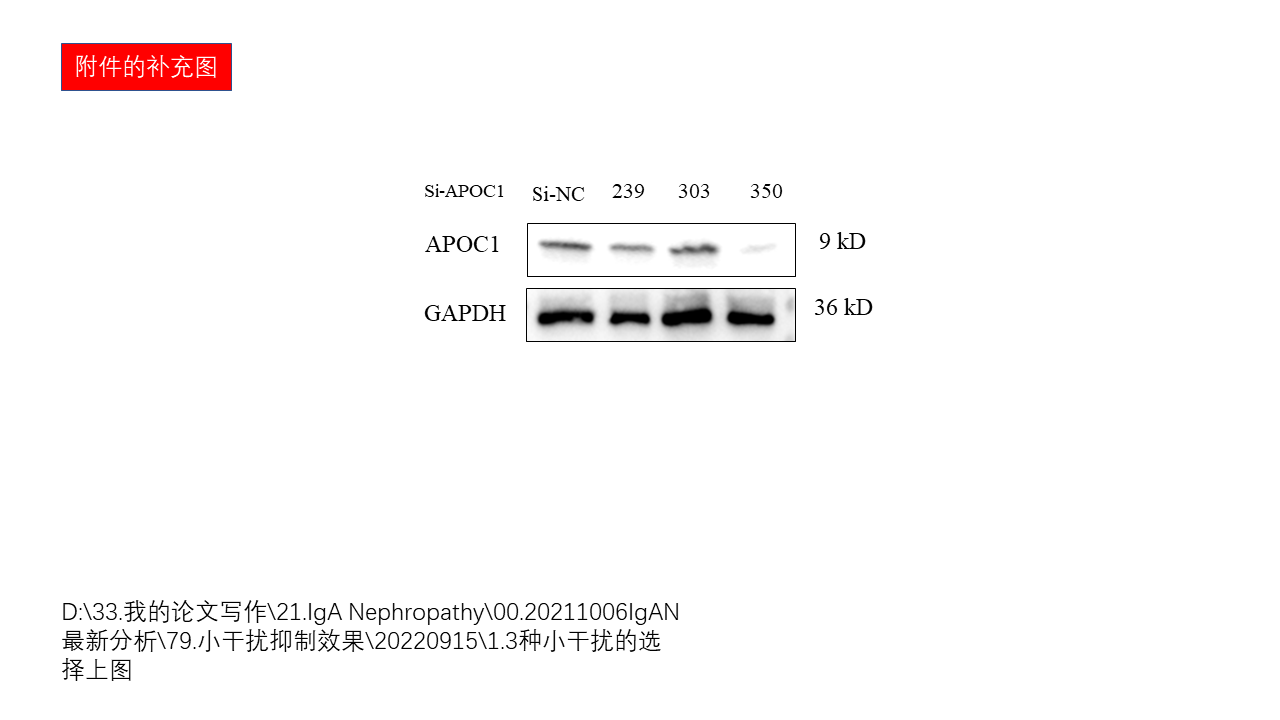


**Sequence of Si-APOC1**

**1.Si-APOC1-homo-239**

forward 5’- GUGCCUUGGAUAAGCUGAATT-3’,

reverse 5’-UUCAGCUUAUCCAAGGCACTT -3’;

**2.Si-APOC1-homo-303**

forward 5’- CCGCAUCAAACAGAGUGAATT-3’,

reverse 5’- UUCACUCUGUUUGAUGCGGTT-3’;

**3.Si-APOC1-homo-350**

forward 5’-CAGAGACAUUUCAGAAAGUTT -3’,

reverse 5’- ACUUUCUGAAAUGUCUCUGTT-3’;


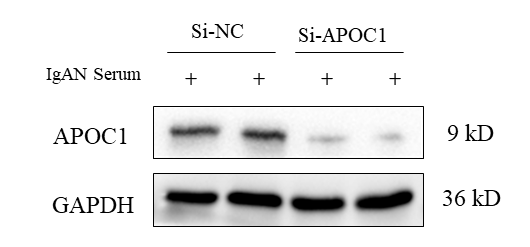

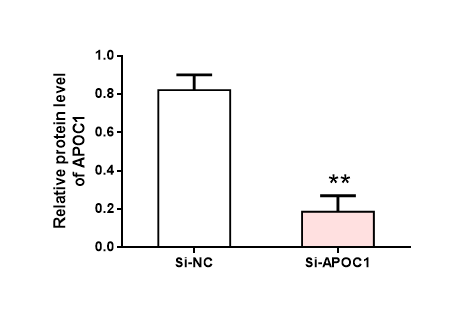


**Representative WB demonstrates APOC1 expression in HK-2 cells after Si-APOC1(Si-APOC1-homo-350)**

**2. Application of plasmids to overexpress APOC1 in HK-2 cells**

Homo sapiens apolipoprotein C1 (APOC1), transcript variant 4, mRNA

NCBI Reference Sequence: NM_001379687.1

GCCACCATGAGGCTCTTCCTGTCGCTCCCGGTCCTGGTGGTGGTTCTGTCGATCGTCTTGGAAGGCCCAGCCCCAGCCCAGGGGACCCCAGACGTCTCCAGTGCCTTGGATAAGCTGAAGGAGTTTGGAAACACACTGGAGGACAAGGCTCGGGAACTCATCAGCCGCATCAAACAGAGTGAACTTTCTGCCAAGATGCGGTTAGAACCCTTCCCAGGGCACGGGAGAGCTGGGGTGTGTTTTTGGGTGGAGCCCTGGCAGATGGTCCAAGATGAACAGATTGAAAAAAAAACAAGTCCTGGAGAGGCTGACAACATCCCTCTGGTCACACAGCTAGATCTCAAGGGAGTGGTTTTCAGAGACATTTCAGAAAGTGAAGGAGAAACTCAAGATGATTACAAGGACGACGATGACAAGtga

**Vector selection pCDNA3.1, gene APOC1, C-terminal fusion Flag tag and Carrier atlas**


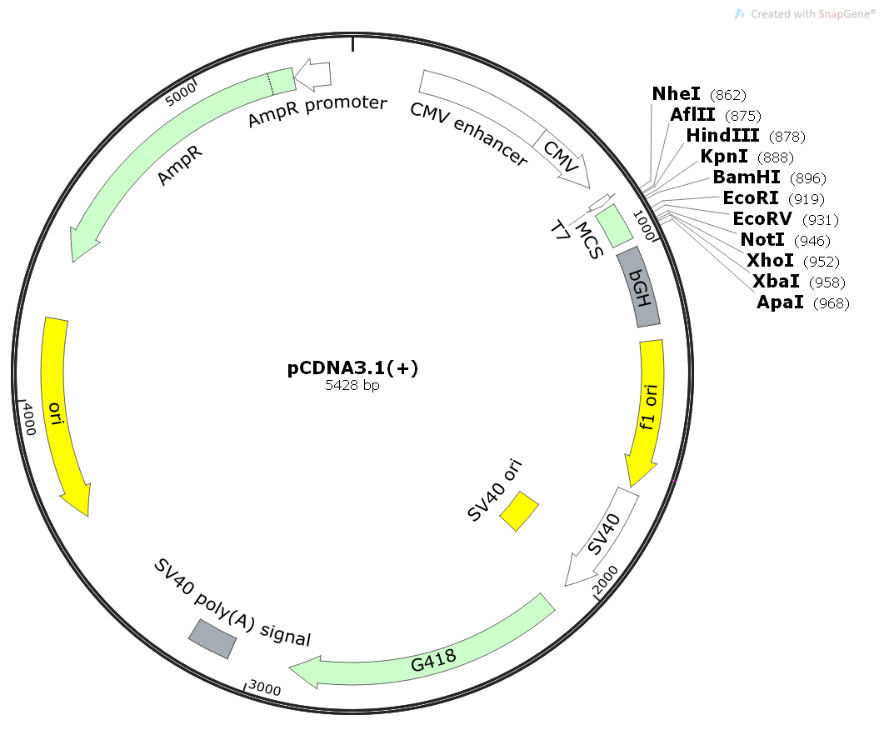

Supplement: Supplementary file 8 [file Table8.DOCX]
